# Catalyst‐Free Selective Reduction of Nitrogen Dioxide to Nitric Oxide

**DOI:** 10.1002/chem.71267

**Published:** 2026-06-18

**Authors:** Awais W. Seyyad, Agamemnon E. Crumpton, Reece Lister‐Roberts, Meera Mehta

**Affiliations:** ^1^ Department of Chemistry University of Oxford Oxford UK

**Keywords:** catalyst‐free, main group chemistry, nitric oxide, nitrogen dioxide, reduction

## Abstract

Nitrogen dioxide (NO_2_) is a highly toxic gas and a notorious respiratory irritant that contributes to the greenhouse effect and acid rain. Herein, we study the catalyst‐free reduction of NO_2_ with boranes, silanes, and the hydrogen surrogates 1,4‐cyclohexadiene and isopropyl alcohol, resulting in the selective formation of nitric oxide (NO). Selective NO generation was confirmed by electron paramagnetic resonance spectroscopy and low‐temperature ^15^N{^1^H} nuclear magnetic resonance spectroscopy. Additionally, density functional theory calculations have been employed to probe the mechanism of these reductions.

## Graphical abstract

Common main group reductants selectively convert nitrogen dioxide to nitric oxide in a catalyst‐free manner. Computational studies suggest divergent reaction pathways depending on the reductant employed. Where boranes and silanes proceed via a two‐electron process, and hydrogen surrogates undergo stepwise one‐electron processes.

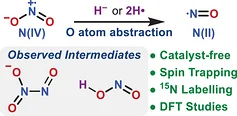

## 1 Introduction

Combustion engines are pervasive in industry and transportation, and at elevated temperatures, they generate nitrogen oxides, namely nitrogen dioxide (NO_2_) and nitric oxide (NO). The generation of NO_2_ is particularly undesirable due to its deleterious environmental and health effects [1]. Exposure to NO_2_ has been associated with increased mortality and heightened susceptibility to cardiovascular and respiratory disease [2]. Additionally, NO_2_ has been implicated in the formation of tropospheric ozone [3], photochemical smog [4], acid rain [5], and the indirect enhancement of the greenhouse effect [6]. Current technologies that degrade NO_x_ emissions require the use of noble metal catalytic converters [7]. These catalysts operate at elevated temperatures (> 200 °C) and rely on scarce, expensive, and depleting transition metals.

NO_2_ can be degraded by single‐electron reduction to nitrite salts or nitrous acid (HONO), or via a two‐electron reduction to NO (Figure 1). In contrast to NO_2_, NO is considerably less toxic and is stable *in vivo* [8]. NO plays a critical role as a biochemical signaling molecule, responsible for mediating a wide array of physiological responses. Mineral aerosol substrates [9, 10], polycyclic aromatic hydrocarbons [11], and hydrated bulk minerals [12] are all known to mediate single‐electron pathways for NO_2_ degradation, while two‐electron pathways are less widely explored. In 2012, Mullins reported the complete and selective reduction of NO_2_ to NO on hydrogen‐precovered Au at cryogenic temperatures [13]. Later, in 2021, Grützmacher reported a homogeneous Rh(I)–Pt(II) based heterobimetallic catalyst capable of hydrogenating NO_x_ gases, including NO_2_ [14]. Furthermore, a recent report by Chen and co‐workers illustrates how mixtures of oxalic acid and Fe_2_O_3_ can also facilitate this transformation [15]. There has been global interest in developing catalyst‐free protocols as sustainable alternatives to transition‐metal mediated processes. Herein, we study the reactivity of organo‐main group reductants, including boranes, silanes, cyclohexadiene, and isopropyl alcohol, with NO_2_ in the absence of a catalyst and demonstrate the selective production of NO.

**FIGURE 1:**
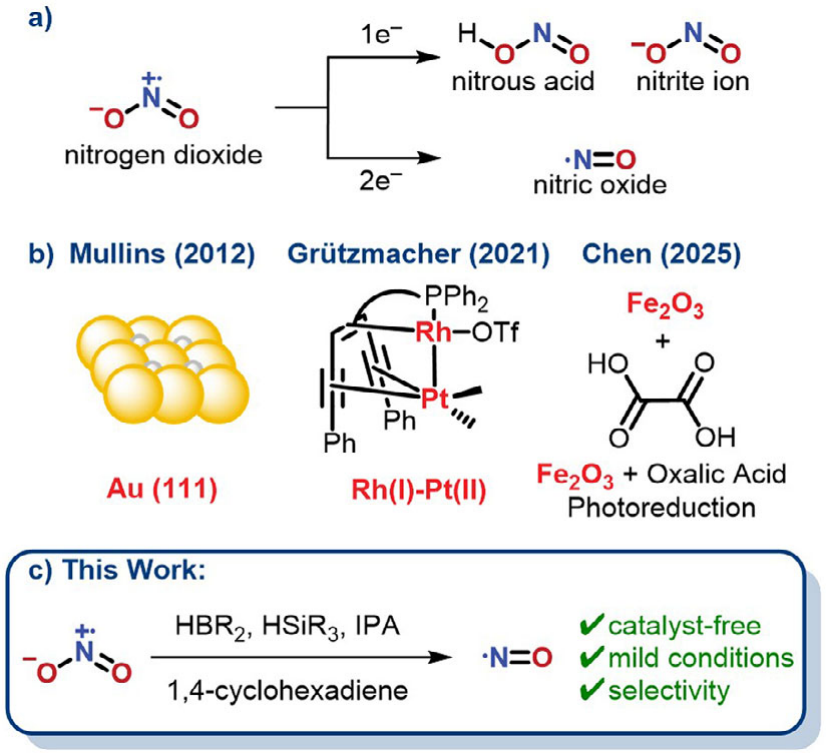
(a) Products from NO_2_ reduction. (b) Transition metal‐mediated reductions of NO_2_. (c) This work, catalyst‐free reduction of NO_2_ by main group reductants.

## 2 Results and Discussion

First, excess NO_2_ (∼1 atm, 25°C) was added to a degassed C_6_D_6_ solution of 4,4,5,5‐tetramethyl‐1,3,2‐dioxaborolane (HBpin), yielding an orange solution. Rapid gas evolution was observed, and the reaction mixture gradually turned green over the course of 5 min. Freezing the reaction mixture in liquid nitrogen (−196°C) after 5 min resulted in the formation of a bright blue solid. The observed thermochromism is consistent with the formation of dinitrogen trioxide (N_2_O_3_), a known product formed from NO and NO_2_ coupling [16, 17]. After 30 min, the reaction mixture was analyzed by ^1^H and ^11^B{^1^H} NMR spectroscopy, which revealed the complete consumption of HBpin and formation of HOBpin and pinBOBpin (88% and 12%, respectively, by ^1^H NMR using toluene as an internal standard) and H_2_ (Figure 2). The reaction mixture was then cooled to −20°C, to freeze any residual NO_2_ (which has a melting point of −11.2°C) [17], and the remaining volatile products were vacuum transferred to an electron paramagnetic resonance (EPR) tube. Initially, no resonances could be detected by EPR spectroscopy at room temperature or −123°C (150 K). A separate control experiment of only NO_2_ in C_6_D_6_ confirmed that NO_2_ gives rise to an EPR resonance as a broad singlet at g = 2.001. Following the addition of excess HBpin to a solution of NO_2_, no resonances could be detected by EPR spectroscopy, indicating the complete consumption of NO_2_. It has previously been reported that it can be challenging to directly observe NO by EPR spectroscopy, due to rapid spin relaxation arising from large spin‐orbit coupling and collisional broadening effects associated with gaseous radicals [18, 19]. Thus, the reaction was repeated as initially described and, after freezing the solution, the headspace was transferred to an EPR tube containing a solution of [Fe(detc)_3_] (detc = diethyldithiocarbamate), a known NO spin trap [20]. Analysis of this reaction mixture by continuous wave (CW) EPR spectroscopy successfully detected the EPR active complex [Fe(NO)(detc)_2_]. Additionally, known NO trapping reagent [T(O‐Me)PP(Co)] was employed in a similar manner to further confirm the generation of NO. Analysis of the reaction mixture by ^1^H NMR spectroscopy confirmed the formation of [T(O‐Me)PP(Co)(NO)], as expected (see Section S6.2). These experiments confirm the formation of NO in the headspace when HBpin is allowed to react with NO_2_.

**FIGURE 2:**
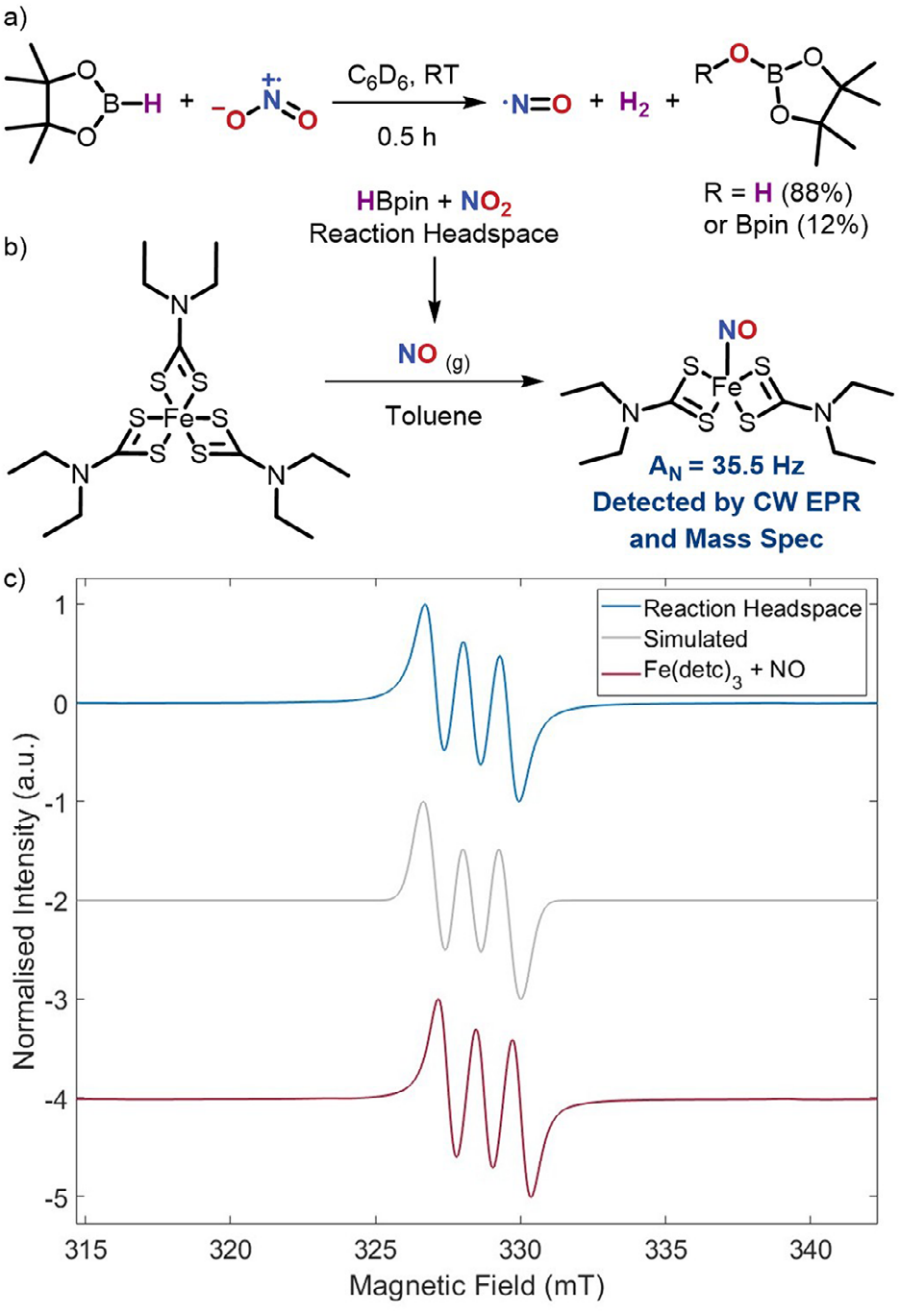
(a) Reduction of NO_2_ with HBpin. (b) Spin trapping of NO by [Fe(detc)_3_] to generate the EPR active species [Fe(NO)(detc)_2_]. (c) Experimental CW EPR spectrum of spin‐trapped NO by [Fe(detc)_3_] at room temperature (experimental parameters: solvent: toluene, frequency: 9.367292 GHz, receiver gain: 30 dB, g = 2.038, blue). Simulation of experimental CW EPR spectrum (1 x A_N_ = 35.5 Hz) (grey). CW EPR spectrum of independently synthesized [Fe(NO)(detc)_2_] from the reaction [Fe(detc)_3_] with NO at room temperature (experimental parameters: solvent: toluene, frequency: 9.372613 GHz, receiver gain: 30 dB, g = 2.038, red).

Isotopically labelled ^15^NO_2_ was generated by reacting ^15^N‐labelled nitric acid with elemental copper [21]. The gaseous product was then vacuum transferred to a separate ampoule and repeatedly degassed to remove any traces of ^15^NO (see Sections S5 and S8). A degassed solution of HBpin in d_8_‐toluene was then pressurized with ^15^NO_2_, and was allowed to react at room temperature for 10 min. The reaction mixture was then cooled to −50°C, at which point a distinct blue color was observed. The reaction mixture was subsequently analyzed by ^15^N{^1^H} NMR spectroscopy at −50°C. At this temperature, NO_2_ is known to dimerize to form the diamagnetic product N_2_O_4_. Additionally, the diamagnetic adduct of NO and NO_2,_ N_2_O_3_, is also known to form. It is worth noting that the dimer of NO, N_2_O_2_, is significantly weaker and more unstable [22], and thus was not anticipated to be detectable by NMR spectroscopy in these experiments. Low‐temperature ^15^N{^1^H} NMR spectroscopy confirmed the presence of N_2_O_3_ with two broad resonances at 671 and 439 ppm, and N_2_O_4_ at 360 ppm (Figure 3). Upon warming to room temperature, no resonances could be detected by ^15^N{^1^H} NMR spectroscopy, in line with the formation of diamagnetic adducts at low temperature as opposed to persistent diamagnetic species. These experiments also show that NO_2_ and NO, along with their corresponding dimers at low temperature, are the only nitrogen‐containing products in the reaction mixture. The reaction mixture was allowed to react for a further 15 min at room temperature, after which it was cooled to −50°C and reanalyzed by ^15^N{^1^H} NMR spectroscopy. No resonances could be detected, suggesting the complete hydroborative reduction of ^15^NO_2_ to ^15^NO. The reaction headspace was vacuum transferred to a J‐Young EPR tube charged with a solution of [Fe(detc)_3_] in toluene, and EPR spectroscopy again confirmed the formation of [Fe(^15^NO)(detc)_2_] (with a distinct N hyperfine coupling x1 A_N_ = 45.5 Hz), the ^15^NO spin trapped product. The larger hyperfine coupling is in agreement with ^15^N's larger gyromagnetic ratio [23].

**FIGURE 3:**
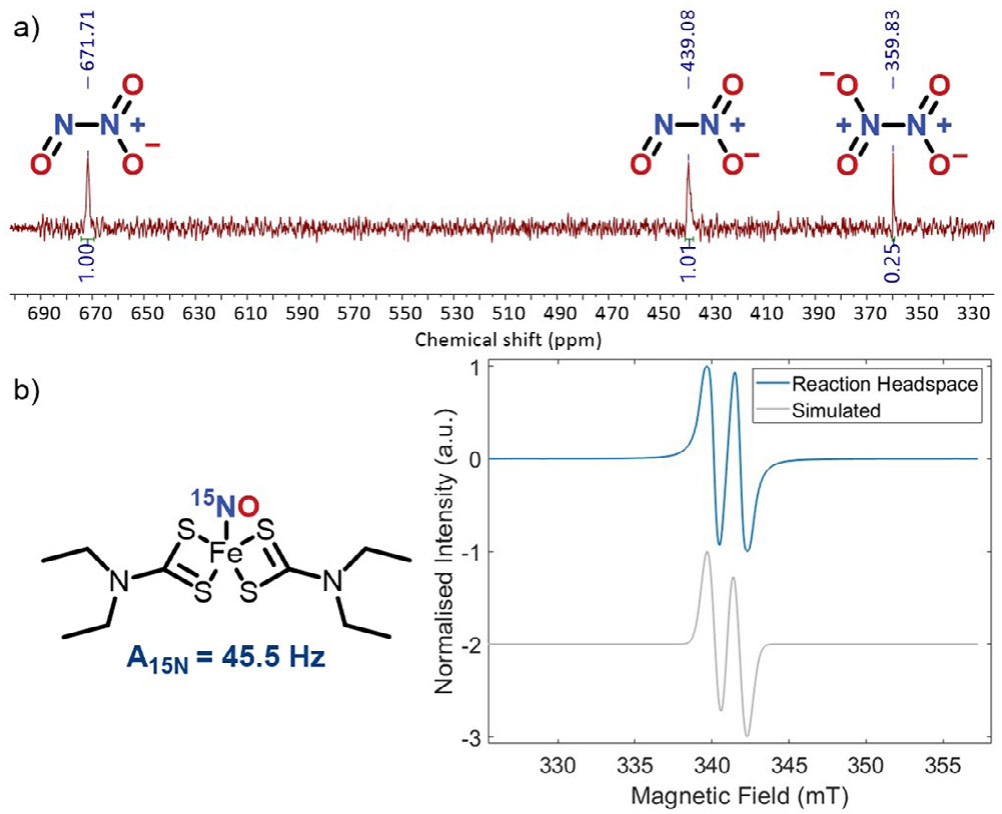
(a) Low‐temperature (−50°C) ^15^N{^1^H} NMR spectrum showing N_2_O_3_ and N_2_O_4_. (b) Spin trapping of ^15^NO and CW EPR spectrum at room temperature (experimental parameters: solvent: toluene, frequency: 9.739740 GHz, receiver gain: 30 dB, g = 2.038, blue) and simulation of [Fe(^15^NO)(detc)_2_] (1 x A_N_ = 45.5 Hz, grey).

In order to gain insight into the mechanism, several pathways were postulated and computationally assessed using r^2^SCAN‐3c composite method with conductor‐like polarizable continuum model (CPCM) benzene solvation. Given the radical nature of NO_2_, single‐electron mechanisms were considered. However, mechanisms involving H atom abstraction or the homolytic cleavage of HBpin were found to be energetically inaccessible (see Section S9.1). Furthermore, experimental observations indicate that pathways involving ^•^Bpin are unlikely, as evidenced by B_2_pin_2_ showing no reaction with NO_2_. Thus, two‐electron hydride mechanisms were explored computationally. Figure 4 summarizes a computationally viable mechanism, with adduct formation between NO_2_ and HBpin being rate‐determining to yield **Int‐1**. Next, **Int‐1** can rearrange into **TS‐2** in a near‐barrierless fashion, followed by hydride migration to give HOBpin and NO via **TS‐3**. Following on from **Int‐1**, both three‐ and five‐membered transition states were also considered and discounted due to their higher energetic barriers (See Sections S9.2 and S9.3).

**FIGURE 4:**
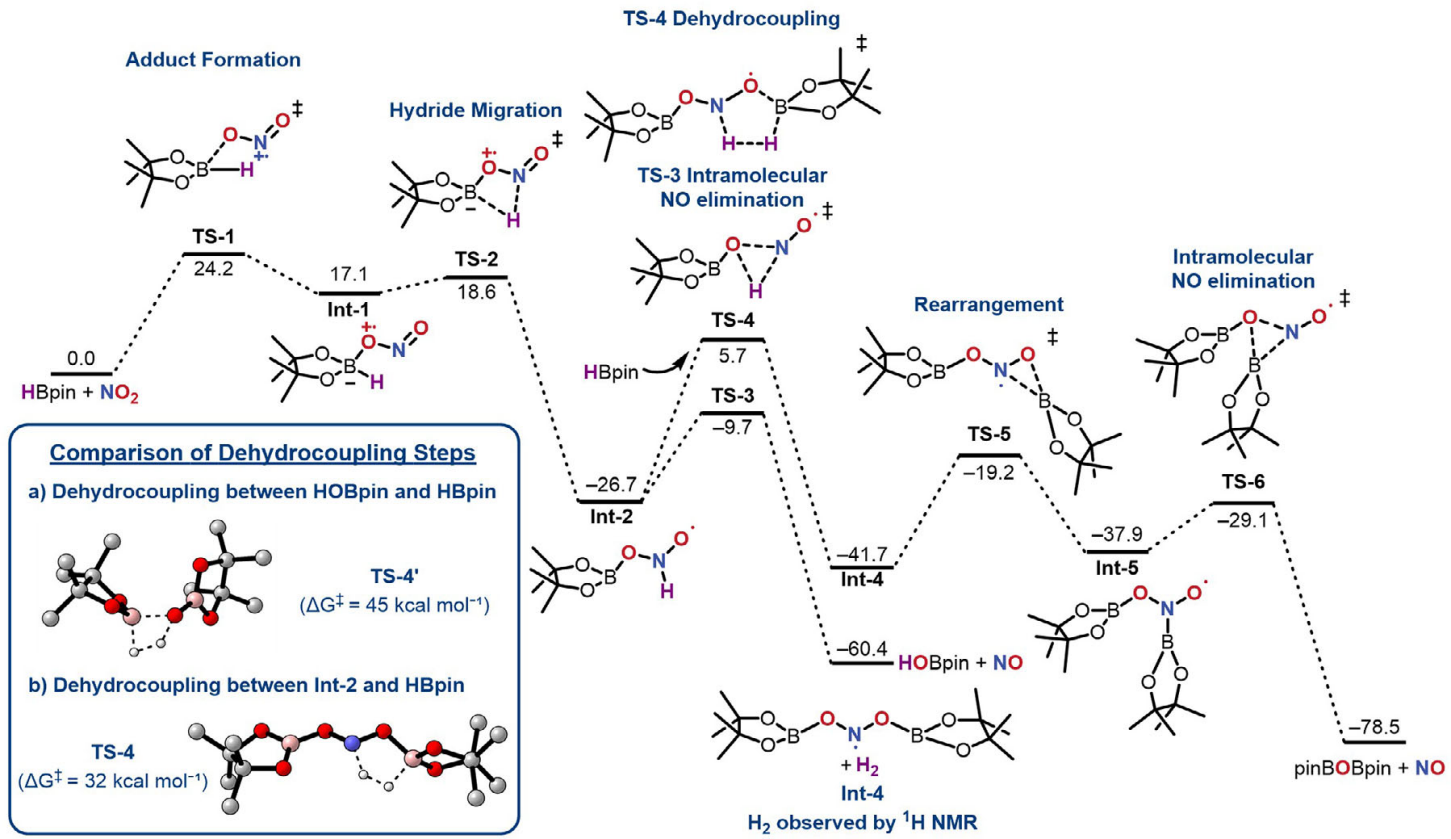
Calculated reaction pathway for the formation of HOBpin, pinBOBpin, and H_2_ gas from the reduction of NO_2_ with HBpin. Gibbs energies in kcal mol^−1^ (r^2^SCAN‐3c composite method with CPCM benzene solvation). Intermediates have been drawn as Lewis structures, with spin density calculations provided in Section S9.5.

Since both HOBpin, pinBOBpin, and H_2_ are observed in the reaction mixture, we postulated that HOBpin could react with excess HBpin in the reaction mixture to give pinBOBpin and H_2_. However, when independently synthesized HOBpin was reacted with one equivalent of HBpin in C_6_D_6_, no reaction was observed at room temperature over the course of a day. Furthermore, the direct dehydrocoupling between HOBpin and HBpin was computed to be prohibitively high (**TS‐4’** ΔG^‡^ = 45.3 kcal mol^−1^). Although, it should be noted that analogous dehydrocoupling reactions can be mediated by Brønsted‐Lowry bases or Lewis acids [24, 25, 26, 27, 28], and thus these pathways cannot be completely ruled out. Nonetheless, we considered a separate mechanism which could lead to the formation of pinBOBpin and H_2_ (shown in Figure 4). Instead, **Int‐2** can react with a second equivalent of HBpin via **TS‐4** (ΔG^‡^ = 32 kcal mol^−1^) to give **Int‐4**, a diborylated aminyl‐type radical, together with H_2_. The relatively high barrier is consistent with the observed selective formation of HOBpin over pinBOBpin in this reaction (88% vs. 12%).

Next, various main group reducing agents were screened to probe the generality of this method. HB(C_6_F_5_)_2_, catecholborane, and ammonia‐borane were tested and found to yield a complex mixture of products consistent with decomposition products. While triethylsilane (Et_3_SiH) was found to react in an analogous fashion to HBpin, albeit not to completion, affording Et_3_SiOH (74%) and Et_3_SiOSiEt_3_ (10%) over the course of 4 h (Table 1). Triphenylsilane (Ph_3_SiH) displayed similar reactivity, forming only Ph_3_SiOH (74%) after 12 h. Whereas reaction of hexamethyldisilane (Si_2_Me_6_) with NO_2_ showed no reaction even after 12 h. Hydrogen‐transfer reagents were also investigated; these reagents are attractive reductants as they bypass the safety hazards associated with using flammable H_2(g)_ [29, 30]. A degassed solution of 1,4‐cyclohexadiene (CHD) in C_6_D_6_ was reacted with excess NO_2_ (∼1 atm, 25°C), resulting in the immediate formation of a persistent bright green solution. ^1^H and ^13^C{^1^H} NMR spectroscopy confirmed the clean and quantitative conversion of 1,4‐cyclohexadiene to benzene. No other organic products were detected by ^1^H or ^13^C{^1^H} NMR spectroscopy. Water was not observed in the ^1^H NMR spectrum; however, a broad signal at ∼6 ppm was detected, in line with the expected resonance of nitrous acid (HONO) [31], possibly formed by water reacting with N_2_O_3_ [16]. Spin trapping experiments using [Fe(detc)_3_] again confirmed the generation of NO from this reaction mixture. EPR spectroscopy was employed to assess whether the final reduction product was NO or HONO. HONO is known to be unstable and decomposes into NO_2_, NO, and water. If HONO were the final reduction product, we postulated that the regeneration of NO_2_ would be observable by CW EPR spectroscopy. Thus, an EPR tube charged with a solution of excess 1,4‐cyclohexadiene in C_6_D_6_ was degassed and pressurized with NO_2_. The reaction was monitored daily by EPR spectroscopy, and regenerated NO_2_ was not detected over the course of one week. Therefore, it is likely that 1,4‐cyclohexadiene reacts with NO_2_ to give HONO as an intermediate, which is then further reduced to NO. Both radical and proton‐hydride mechanisms were probed computationally, and proton‐hydride step‐wise transfer mechanisms were found to be energetically infeasible (see Section S9.6).

**TABLE 1:** Scope of main group reductants tested in the catalyst‐free reduction of NO_2_.

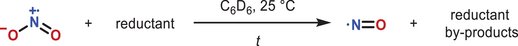		
Reductant	*t*	Reductant consumption (%)^a^
HBpin	30 min	100
B_2_pin_2_	12 h	0
Et_3_SiH	4 h	84
Ph_3_SiH	12 h	74
Si_2_Me_6_	12 h	0
CHD	5 min	100
IPA	12 h	100

^a^ Determined by ^1^H NMR spectroscopy using toluene as an internal standard. CHD = 1,4‐cyclohexadiene; IPA = isopropyl alcohol.

Figure 5 presents a plausible single‐electron transfer mechanism for how 1,4‐cyclohexadiene reduces NO_2_. Significant radical spin density in NO_2_ is located on the N atom [32, 33], which can abstract a H^•^ from 1,4‐cyclohexadiene to give O(H)NO (**Int‐7**) via **TS‐7**, which is slightly more energetically favorable than **TS‐7’**. Intramolecular isomerization of ON(H)O to HONO was found to be kinetically disfavored (see Section SS9.7, **TS‐d**: ΔG^‡^ = 47.2 kcal mol^−1^). However, isomerization via a six‐membered intermolecular transition state, **TS‐8**, is energetically feasible and converts **Int‐7** to HONO (**Int‐8**). Finally, HONO reacts with cyclohexadienyl radical to afford NO, benzene, and water. This computed mechanism is in agreement with Mullins’ gold‐mediated hydrogenation mechanism, where hydrogen atoms first reduce NO_2_ to HONO, which is then further reduced to NO [13].

**FIGURE 5:**
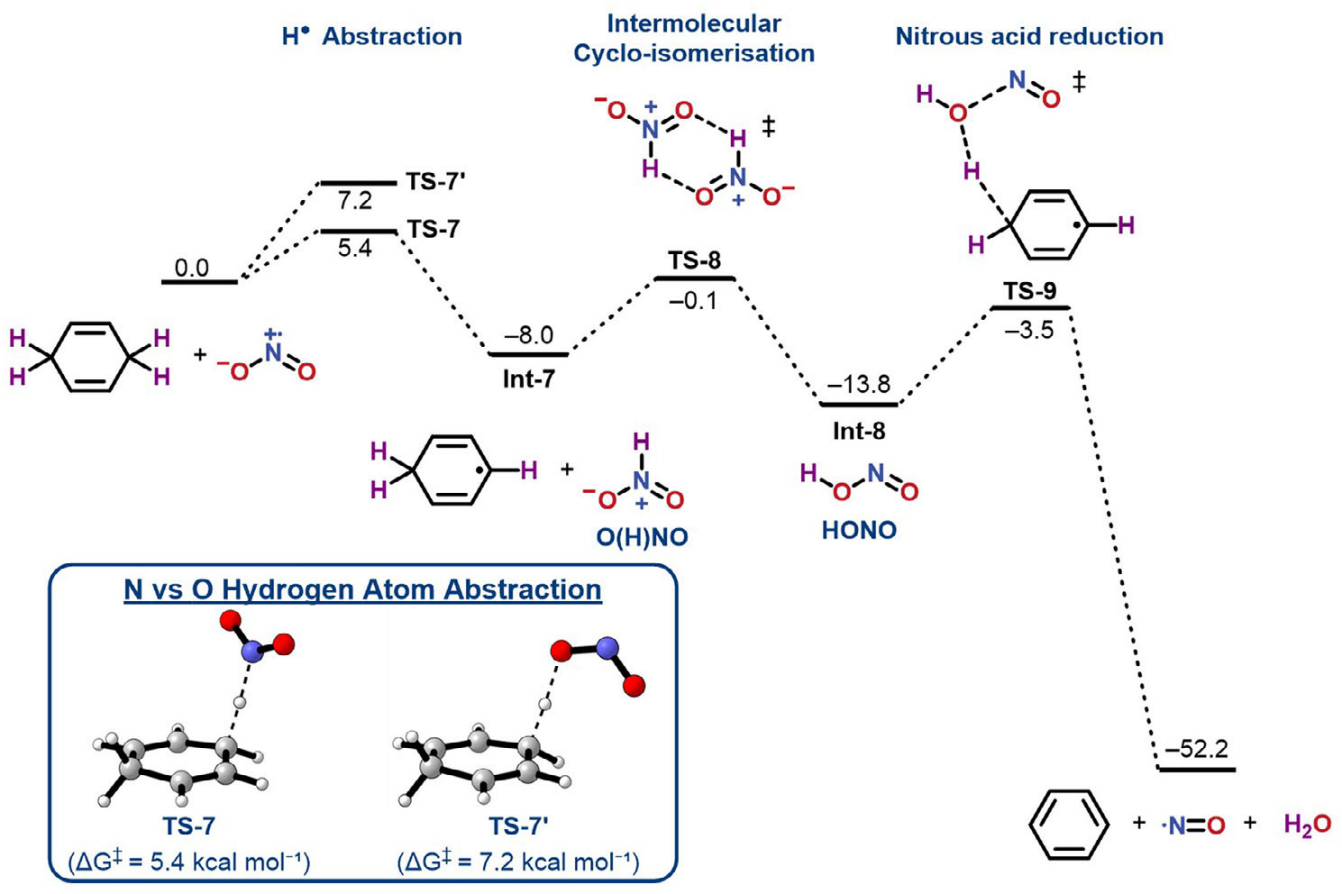
Calculated reaction pathway for the reduction of NO_2_ by 1,4‐cyclohexadiene. Gibbs energies in kcal mol^−1^ (r^2^SCAN‐3c composite method with CPCM benzene solvation).

Isopropyl alcohol (IPA) was also tested as a hydrogen‐transfer reductant. When reacted with excess NO_2_, IPA was fully consumed within 12 h, forming only acetone (27%) and isopropyl nitrite (73%), confirmed by ^1^H and ^13^C{^1^H} NMR spectroscopy. A broad resonance at ∼6 ppm was again observed in the ^1^H NMR spectrum, suggesting HONO as an intermediate. Furthermore, the formation of isopropyl nitrite is consistent with the presence of HONO in the reaction mixture [34]. Additionally, N_2_O_3_ (the NO_2_ and NO adduct) is also known to be an active nitrosating agent for alkyl alcohols [35]. Spin trapping experiments using Fe(detc)_3_confirmed the formation of NO when IPA was employed as a reductant.

## 3 Conclusion

In summary, we report the selective reduction of NO_2_ to NO using common main group reducing agents, including boranes, silanes, and hydrogen‐transfer reagents, in a catalyst‐free manner. Selective NO formation was confirmed by EPR studies, including the use of NO‐trapping reagents, and labelled ^15^N NMR experiments. The mechanisms for these reductions were investigated computationally. The borane and silane mediated pathways are believed to differ from those involving hydrogen‐transfer reagents such as 1,4‐cyclohexadiene and isopropyl alcohol, with the latter possibly proceeding via HONO as an intermediate.

## Conflicts of Interest

The authors declare no conflicts of interest.

## Supporting information




**Supporting File 1**: The data supporting this article have been included as part of the Supplementary Information (.pdf).

## Data Availability

The data that supports the findings of this study are available in the supplementary material of this article.
