# Supplementary material for: Catalyst‐Free Selective Reduction of Nitrogen Dioxide to Nitric Oxide
Source: Chemistry. 2026 Jun 18;32(37):e71267. doi: 10.1002/chem.71267 (PMC13628534; doi:10.1002/chem.71267)
Supplement: Supplementary file 1 [file CHEM-32-e71267-s001.pdf]

Catalyst-Free Selective Reduction of Nitrogen Dioxide to Nitric Oxide

Supplementary Information

Awais Seyyad,[a] Agamemnon E. Crumpton,[a] Reece Lister-Roberts,[a] and Meera Mehta[a]*

[a] Department of Chemistry, University of Oxford, 12 Mansfield Road, Oxford OX1 3TA, U.K.

*meera.mehta@chem.ox.ac.uk

Contents

1. Methods and Materials ... 3
1.1 Experimental Considerations ... 3
1.2 Analytical Considerations ... 3
1.3 General Computation Considerations ... 4
2. Synthesis of literature known compounds ... 4
2.1 Synthesis of Fe(detc)$_3$ ... 4
2.2 Independent synthesis of Fe(NO)detc$_2$ ... 4
3 Control Experiments ... 5
3.1 Benzene solvent compatibility control experiment ... 5
3.2 Toluene internal standard compatibility control experiment ... 6
3.3 [Fe(detc)$_3$] control reaction with nitrogen dioxide ... 7
3.4 [T(O-Me)PP(Co)] control reaction with nitrogen dioxide ... 8
4 NO_2 cylinder contamination ... 8
4.1 Test for NO contamination in commercially purchased NO_2 cylinder ... 8
5. Synthesis and purification of NO_2 ... 9
5.1 Experimental setup to synthesise NO_2 ... 9
5.2 EPR of NO_2 ... 10
5.3 Purification of NO_2 ... 11
6 General Procedure for NO_2 (from independent synthesis, section 5) reduction with main group reducing agents ... 11
6.1 Reduction of NO_2 to NO and subsequent spin trapping by Fe(detc)$_3$ ... 13
6.2 Reduction of NO_2 to NO and subsequent trapping by [T(OMe)PP]Co(II) ... 14
6.3 Characterisation data when $HSiEt_3$ was employed as a reductant ... 16
6.4 Characterisation data when B_2pin_2 was employed as a reductant ... 17
6.5 Characterisation data when Si_2Me_6 was employed as a reductant ... 18

6.6 Characterisation data when $HSiPh_3$ was employed as a reductant 18
6.7 Characterisation data when 1,4-cyclohexadiene was employed as a reductant .. 20
6.8 Characterisation data when isopropyl alcohol was employed as a reductant 21
7 ^{15}N-labelled Experiments 23
7.1 Synthesis and purification of ^{15}N-labelled NO_2 23
7.2 Reduction of 15NO_2 by HBpin 23
8 Mechanistic Investigations 25
8.1 Reaction of NO_2 with Excess HBpin 25
8.2 Attempted synthesis of pinBOBpin from dehydrocoupling with HBpin with HOBpin in C_6D_6 26
8.3 Reaction of NO_2 with Excess 1,4-cyclohexadiene 28
9 Density Functional Theory 28
9.1 Discounted radical HBpin mechanisms 28
9.2 Discounted three-membered HBpin pathway 29
9.3 Discounted five-membered HBpin pathway 29
9.4 Discounted dehydrocoupling mechanism between HOBpin and HBpin 30
9.5 Mulliken Atomic Charges and Spin Populations for HBpin Reduction Intermediates 31
9.6 Discounted proton-hydride mechanisms for transfer hydrogenation 35
9.7 Discounted intramolecular isomerisation of O(H)NO to HONO 36
9.8 DFT Coordinates 37
10 References 59

1. Methods and Materials

1.1 Experimental Considerations

All manipulations were performed under an inert atmosphere using standard Schlenk-line and glovebox (MBraun Unilab) techniques, except for spin trapping and NO quantification experiments described in section 6. Glassware was flame-dried prior to use.

Dry toluene and dichloromethane (DCM) were obtained using an Innovative Technologies' anhydrous engineering solvent purification system and subsequently degassed. C_6D_6 and $CDCl_3$ were purchased from Sigma-Aldrich and degassed by freeze-pump-thaw cycles. All solvents were stored over activated 3 Å molecular sieves.

Elemental copper (Cu), 68% nitric acid (HNO_3), Nitric-^{15}N acid ($H^{15}NO_3$), sodium diethyldithiocarbamate trihydrate ($NaS_2CNEt_2.3H_2O$), iron (III) trichloride ($FeCl_3$), pinacol borane (HBpin), bis(pinacolato)diboron (B_2Pin_2), triethylsilane (Et_3SiH), triphenylsilane (Ph_3SiH), hexamethyldisilane (Si_2Me_6), 1,4-cyclohexadiene (CHD), isopropyl alcohol (IPA) and 5,10,15,20-tetrakis(4-methoxyphenyl)-21H,23H-porphine cobalt(II) ([T(O-Me)PP(Co)]) were purchased from commercial sources (Sigma-Aldrich, Alfa Aesar, and Tokyo Chemical Industry). NO_2 and NO were purchased from CK Gas Products Limited. Fe(detc)$_3$ and Fe(NO)(detc)$_2$ (detc = S_2CNEt_2) were synthesised in accordance with literature procedures.[1]

1.2 Analytical Considerations

NMR Spectroscopy. ^{1}H, ^{11}B{^{1}H}, ^{13}C{^{1}H}, ^{15}N{^{1}H}, ^{29}Si{^{1}H} NMR spectra were recorded on a Bruker AVIII 400 spectrometer (operating frequencies: 400.17 MHz, 128.36 MHz, 100.53 MHz, 79.48 MHz and for ^{1}H, ^{11}B, ^{13}C, and ^{29}Si respectively) or Bruker AVIII 500 spectrometer (operating frequencies: 499.94 MHz, 160.40 MHz, 125.72 MHz, and 50.67 MHz for ^{1}H, ^{11}B, ^{13}C, and ^{15}N, respectively). ^{1}H and ^{13}C{^{1}H} NMR chemical shifts were internally referenced to the residual solvent resonances C_6D_6 (benzene-d$_6$): ^{1}H δ = 7.34 ppm, ^{13}C{^{1}H} δ = 128.02 ppm. ^{11}B, ^{15}N, ^{29}Si NMR chemical shifts were externally referenced to $BF_3 \cdot Et_2O$, NH_3, Me_4Si, respectively. Solution phase NMR samples were prepared under an inert atmosphere in 5 mm J. Young NMR tubes. NMR data was analysed using MestReNova v15.0.0 software.

Electron Paramagnetic Resonance. Continuous wave electron paramagnetic resonance (CW EPR) was acquired in the Centre for Advanced ESR (CAESR) of the Department of Chemistry, University of Oxford. EPR spectra were recorded at X-band (9.4 GHz) with a Bruker EMXmicro with a Premium bridge and an ER4122SHQE-W1 resonator at either 298 K. All EPR spectra were plotted using MATLAB R2024a and simulations conducted using EasySpin 6.0.2 plugin.[2]

Mass spectrometry. Mass spectrometry samples were analysed using an electrospray ionisation-equipped Waters RDa benchtop time-of-flight mass

spectrometer. Samples were prepared and directly injected into the ionisation source of the mass spectrometer.

Ultraviolet–visible spectroscopy. Ultraviolet-visible (UV-Vis) electronic absorption spectra were recorded on a Mettler Toledo UV5Bio spectrophotometer using 10 mm path-length quartz J. Young cuvettes.

1.3 General Computation Considerations

All computational work reported herein was performed using ORCA (revision 6.1.0).[3] All geometries were optimised using the meta–generalised gradient approximation (mGGA) functional r²SCAN,[4] in conjunction with the def2-TZVPPm basis set,[5] including the D4 dispersion correction[6] and the geometrical counterpoise correction gCP[7] (collectively referred to as the r²SCAN-3c method).[5] Solvent effects were approximated using the conductor-like continuum polarisation model (CPCM) with benzene as the solvent.[8] The nature of the stationary points was confirmed by full vibrational frequency calculations: minima are characterised by the absence of imaginary frequencies, while transition states exhibit a single imaginary frequency corresponding to the reaction coordinate.

2. Synthesis of literature known compounds

2.1 Synthesis of $Fe(detc)_3$

To a round-bottom flask charged with a stirrer bar, $FeCl_3$ (162 mg, 1 mmol, 1 equiv) and absolute ethanol (10 mL) were added, forming an orange solution. An ethanolic solution (10 mL) of $NaS_2CNEt_2.3H_2O$ (676 mg, 3 mmol, 3 equiv) was slowly added to the stirring $FeCl_3$ solution, resulting in the immediate formation of a black precipitate. The reaction was stirred for a further 15 minutes, after which the precipitate was isolated by filtration and washed several times with distilled water and absolute ethanol, and then dried under reduced pressure. Characterisation data was consistent with literature reports.[1a]

Isolated Yield: 435 mg, 87%

UV-Vis: 332 nm, 523 nm, 596 nm

Mass Spectrometry (ESI): $[Fe(S_2CN(CH_2CH_3)_2]^+$: calcd: 500.0108, found: 500.0117

2.2 Independent synthesis of $Fe(NO)detc_2$

$Fe(detc)_3$ (5 mg, 0.01 mmol) was dissolved in toluene (200 µL) and added to a J. Young EPR tube. The tube was successively degassed and then pressurised with NO (~1 atm, 25 °C), resulting in a colour change from black to dark brown. The reaction mixture was analysed by EPR spectroscopy and mass spectrometry. Both confirmed the formation of $Fe(NO)(detc)_2$. The EPR g value and hyperfine coupling constant were in agreement with the literature values.[1b]

Mass Spectrometry (ESI): $[Fe(NO)(S_2CN(CH_2CH_3)_2]Na^+$ calcd: 404.9731, found: 404.9743

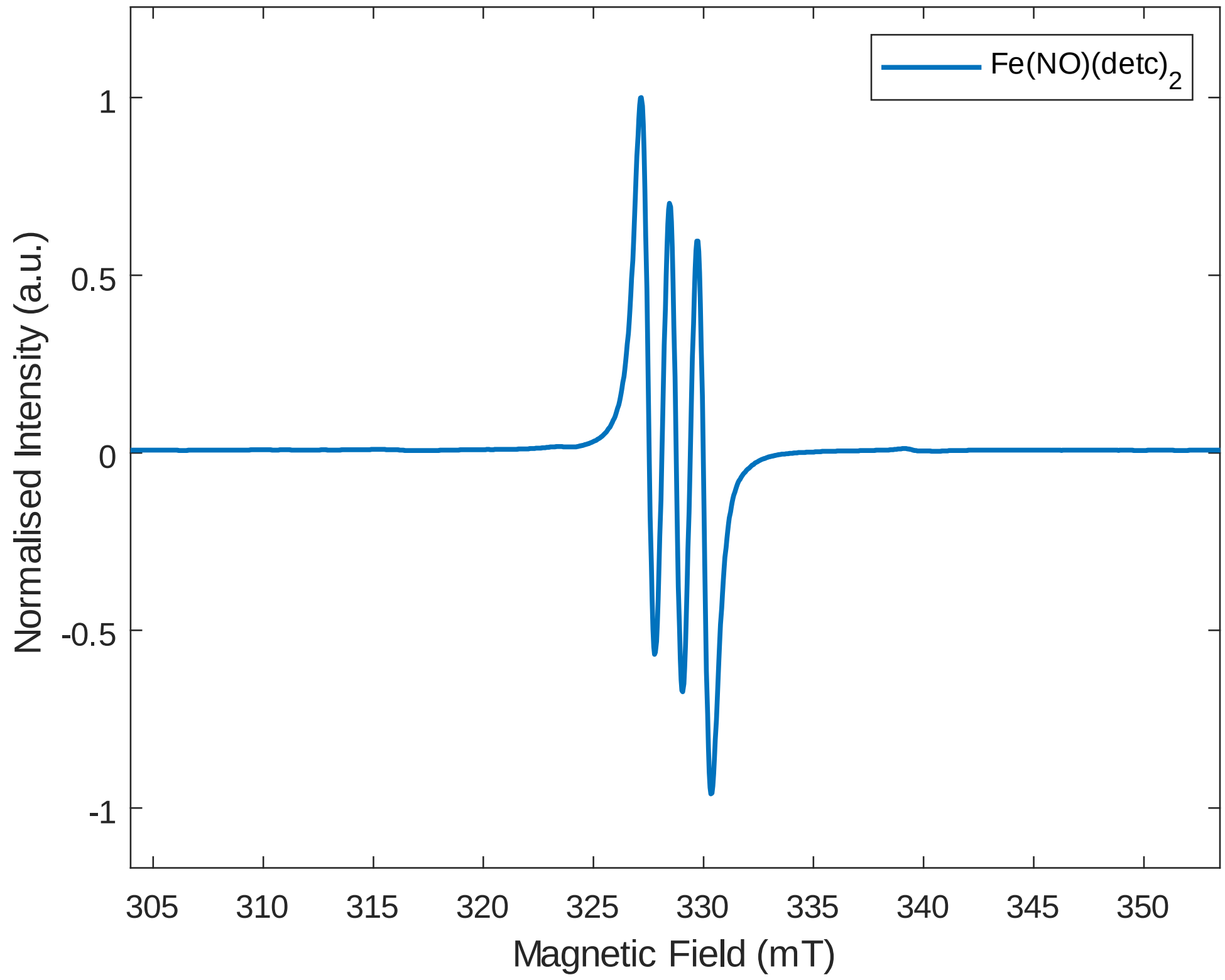

Figure S1. Continuous wave EPR spectra of Fe(NO)(detc)$_2$. Experimental parameters: solvent: toluene, frequency: 9.372613 GHz, temperature: 298 K, modulation amplitude 4, 1 scan, receiver gain 30 dB, g = 2.038, A_N = 35.5 Hz

3 Control Experiments

3.1 Benzene solvent compatibility control experiment

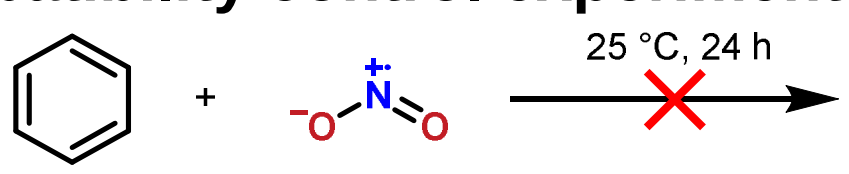

To a J. Young NMR tube, a C_6D_6 capillary and C_6H_6 (0.6 mL, 6.7 mmol) were added and degassed by successive freeze-pump-thaw cycles. The NMR tube was then pressurised with NO_2 (~1 atm, 25 °C). The reaction mixture was monitored by ^{1}H and ^{13}C{^{1}H} NMR spectroscopy. No evidence of nitration was observed at room temperature. Therefore, benzene was selected as an appropriate solvent for this chemistry.

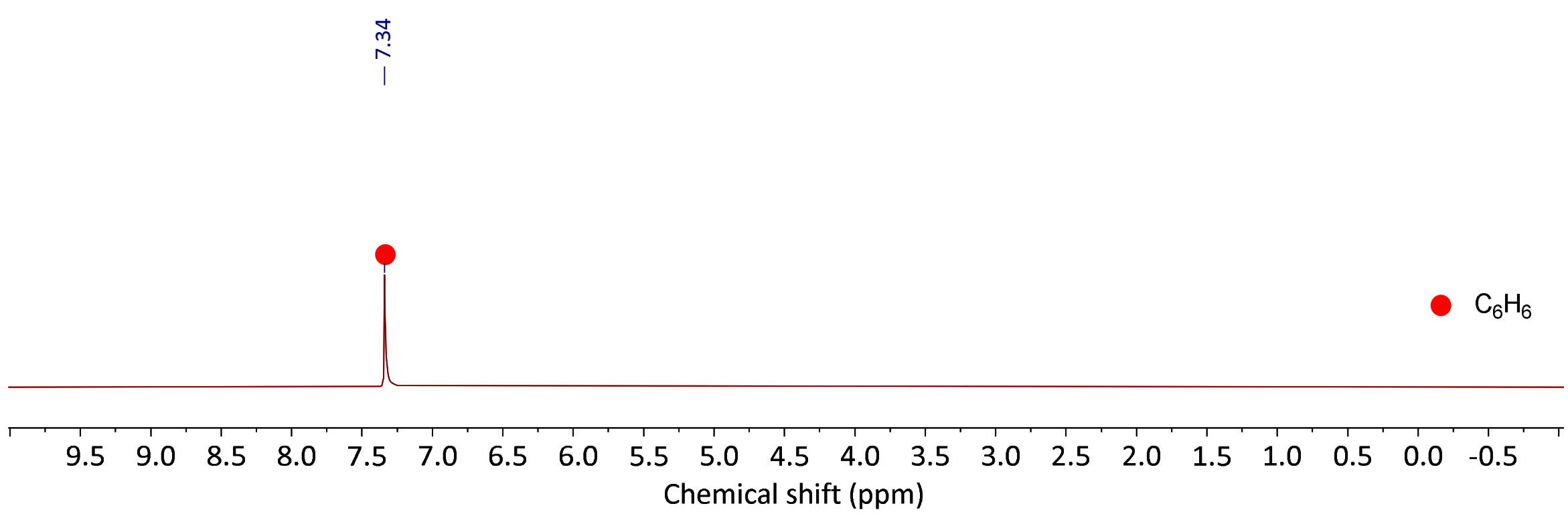

Figure S2. ¹H NMR spectrum (400 MHz, C_6D_6) of NO_2 dissolved in benzene, showing no reactivity after 24 hours at room temperature

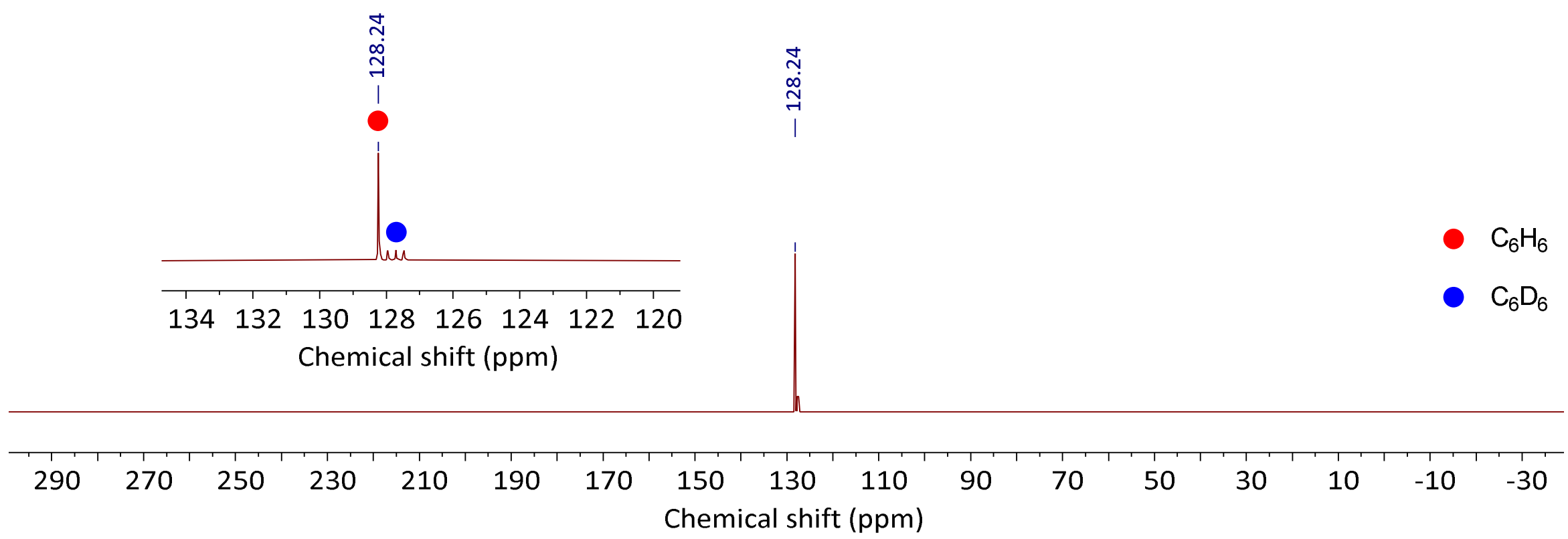

Figure S3. ¹³C{¹H} NMR spectrum (101 MHz, C_6D_6) of NO_2 dissolved in benzene, showing no reactivity after 24 hours at room temperature

3.2 Toluene internal standard compatibility control experiment

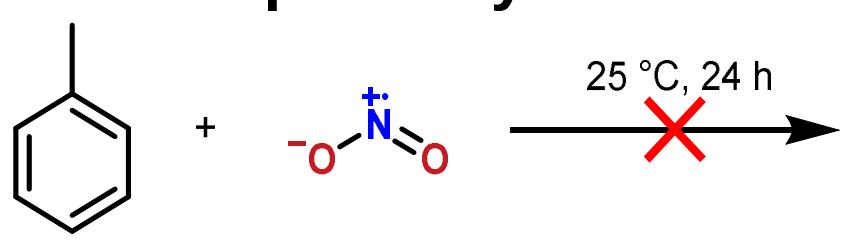

To a J. Young NMR tube, C_7H_8 (10 µL, 0.094 mmol) was added to C_6D_6 (0.6 mL) and successively degassed by freeze-pump-thaw. The NMR tube was then pressurised with NO_2 (~1 atm, 25 °C). The reaction mixture was monitored by ¹H and ¹³C{¹H} NMR spectroscopy. No evidence of nitration of toluene at room temperature was detected, and therefore toluene was selected as an appropriate internal standard for this chemistry.

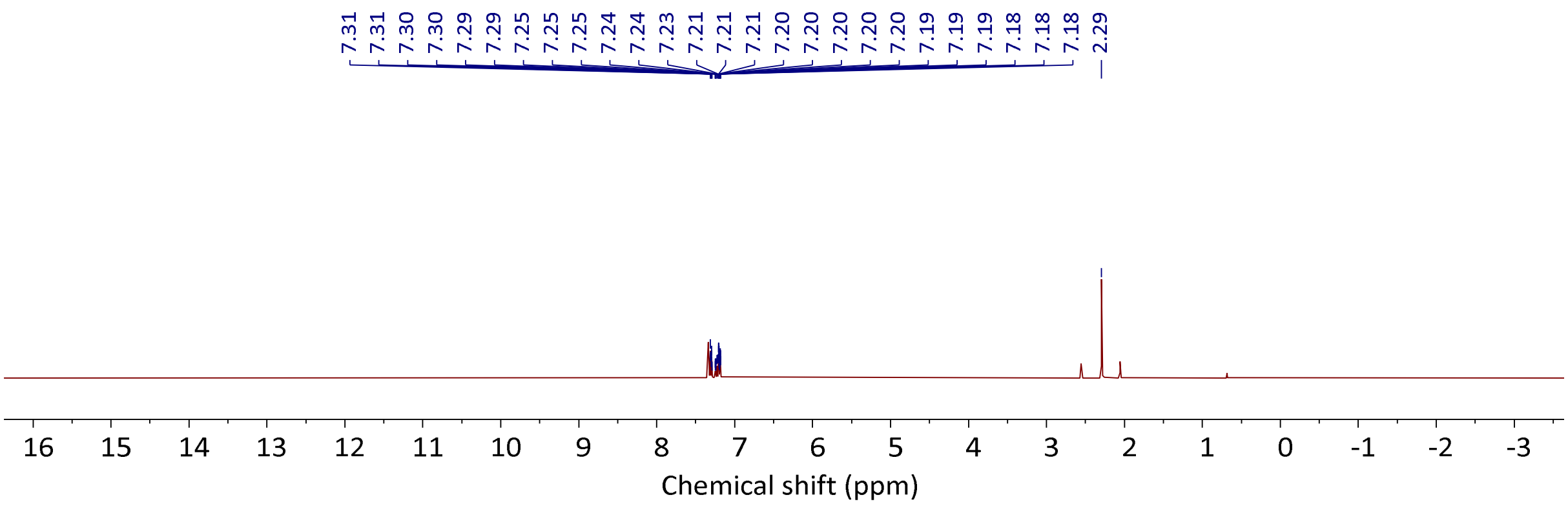

Figure S4. ¹H NMR spectrum (400 MHz, C_6D_6) of a mixture of NO_2 and toluene, showing no reactivity after 24 hours at room temperature

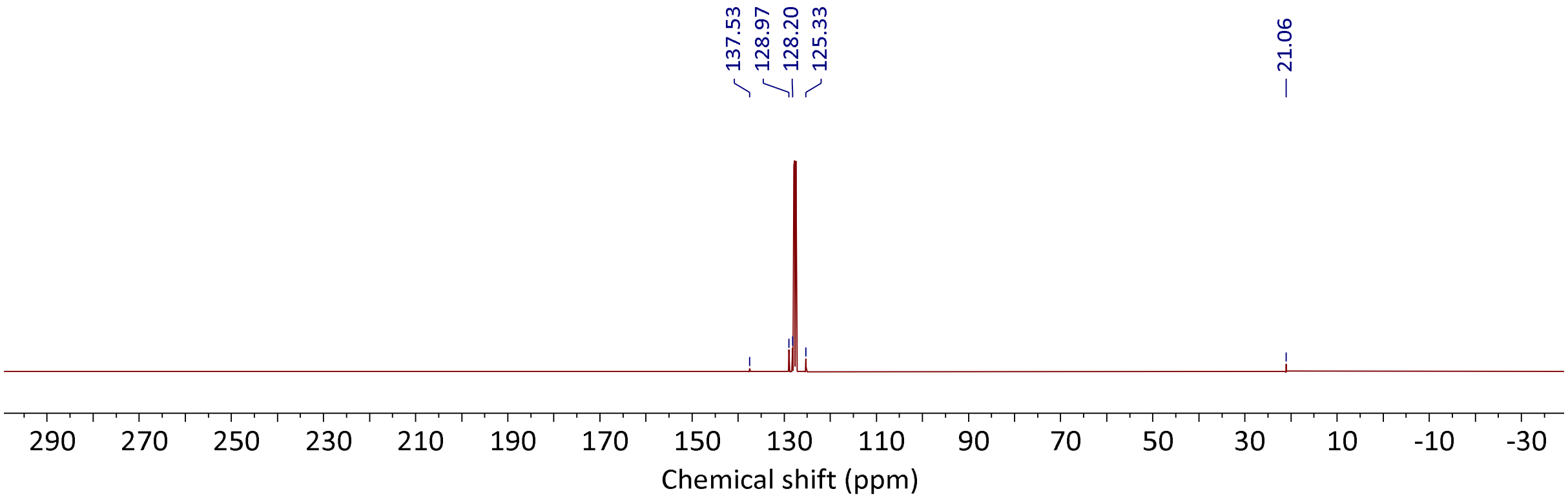

Figure S5. $^{13}C\{^{1}H\}$ NMR spectrum (101 MHz, C_6D_6) of a mixture of NO_2 and toluene, showing no reactivity after 24 hours at room temperature

3.3 [Fe(detc)$_3$] control reaction with nitrogen dioxide

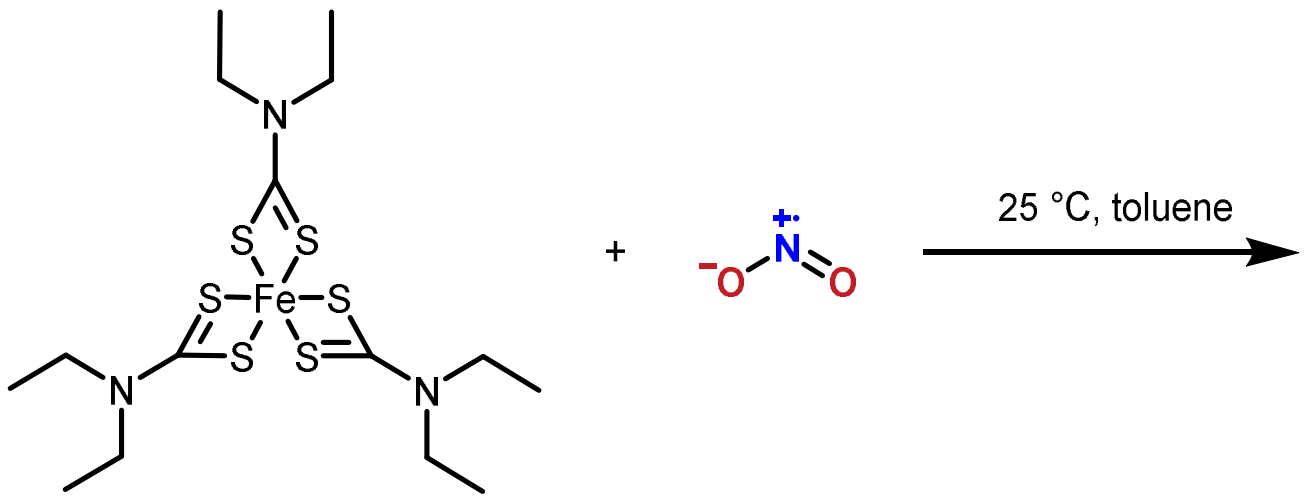

A solution of Fe(detc)$_3$ dissolved in toluene (200 µL, 10 mM) was added to a J. Young EPR tube and successively degassed. The EPR tube was then pressurised with NO_2 (~1 atm, 25 °C), and inverted several times, resulting in a noticeable colour change from black to green. Vigorous gas evolution and the precipitation of light brown solids were observed. Excess NO_2 was removed by evacuating the EPR tube's headspace. The reaction mixture was analysed by EPR spectroscopy and did not yield a resonance at room temperature. This is common for certain transition metal complexes due to inefficient spin relaxation.[9] This confirms that trace NO_2 is not responsible for the EPR signals detected from the reduction products of NO_2.

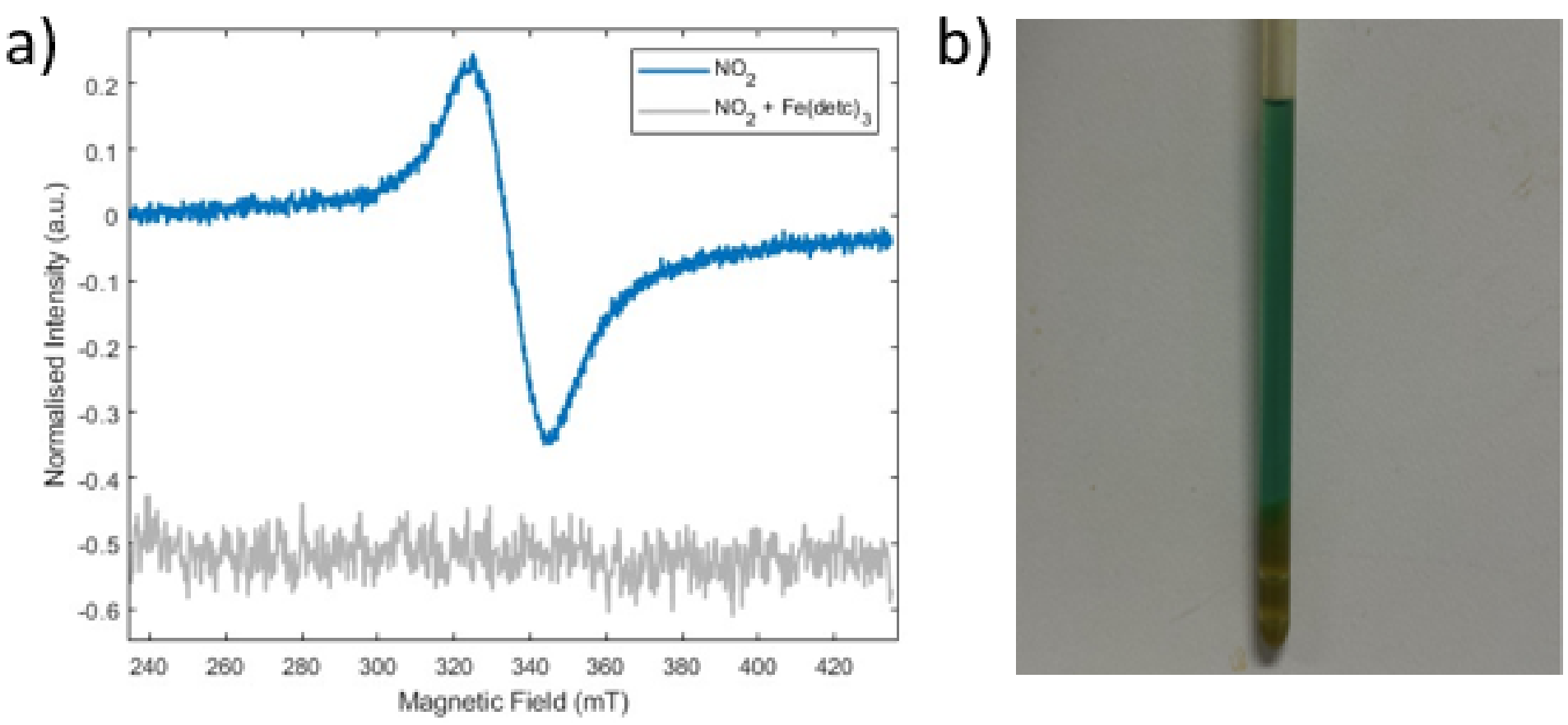

Figure S6. a) Continuous wave EPR spectra of NO_2 (blue) and NO_2 reacting with excess Fe(detc)$_3$ (grey). Experimental parameters: solvent: toluene, frequency:

9.372515 GHz, temperature: 298 K, modulation amplitude 4, 1 scan, receiver gain 30 dB. b) Picture of NO_2 reacting with excess Fe(detc)$_3$.

3.4 [T(O-Me)PP(Co)] control reaction with nitrogen dioxide

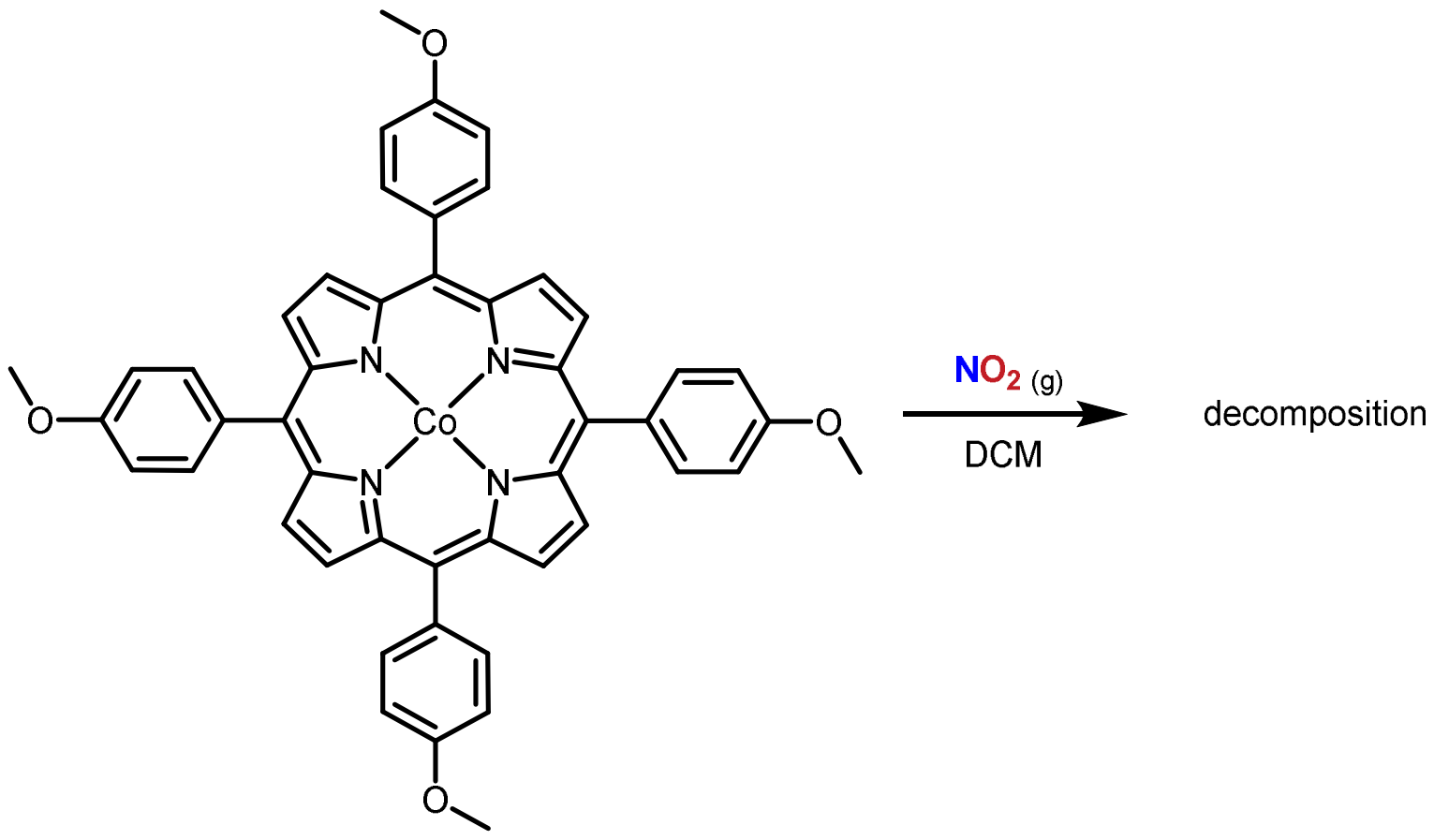

[T(O-Me)PP(Co)] (7.9 mg, 0.01 mmol) was dissolved in toluene (200 µL) and added to a J. Young tube. The NMR tube was successively degassed and then pressurised with NO_2 (~1 atm, 25 °C), resulting in a colour change from purple to black-red. The reaction mixture was analysed by ^1H NMR spectroscopy which indicated the likely decomposition of [T(O-Me)PP(Co)]. A large resonance at δ 3.91 was observed, which does not align with the resonances associated with [T(O-Me)PP(Co)(NO)]. This confirms that trace NO_2 is not responsible for the characteristic ^1H NMR resonance of [T(O-Me)PP(Co)(NO)] detected from the reduction products of NO_2.

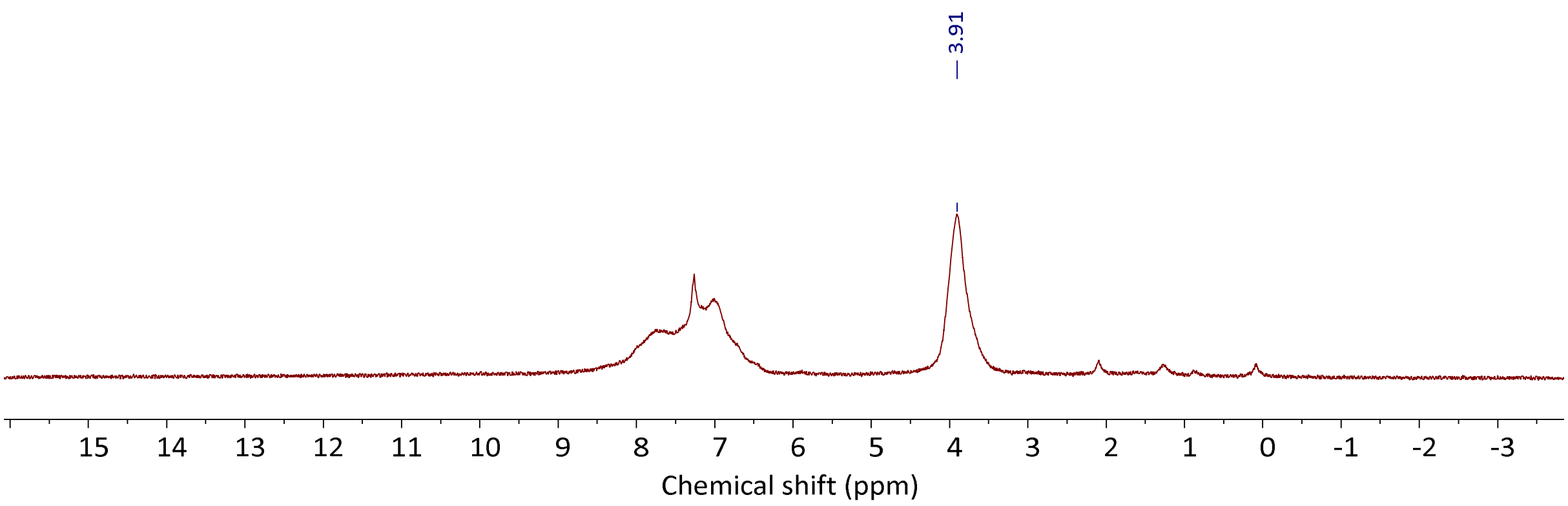

Figure S7. ^1H NMR spectrum (400 MHz, C_6D_6) of a mixture of NO_2 and [T(O-Me)PP(Co)].

4 NO_2 cylinder contamination

4.1 Test for NO contamination in commercially purchased NO_2 cylinder

To a J. Young NMR tube, C_6H_6 (0.6 mL) was added and successively degassed by freeze-pump-thaw. The NMR tube was then pressurised with NO_2 (~1 atm, 25 °C). The NMR tube was then frozen in liquid nitrogen. A faint blue colour was observed, indicating NO contamination. At temperatures under –11 °C, NO_2 dimerises and forms N_2O_4 which is a white solid.[10]

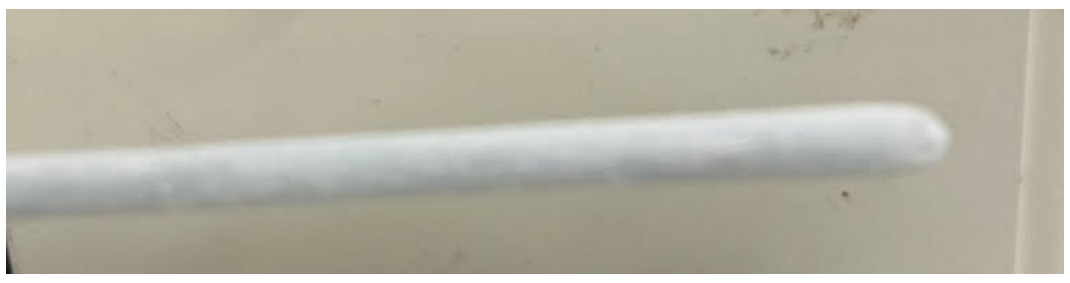

Figure S8. Solution of NO_2 in C_6D_6 at 77 K. A faint blue colour was observed indicative of NO contamination.

A J. Young NMR tube was evacuated and then pressurised with NO_2 (~1 atm, 25 °C). The reaction mixture was cooled to –20 °C to selectively freeze the NO_2. The NMR tube's headspace was then vacuum transferred to an EPR tube charged with a toluene solution of $Fe(detc)_3$ (200 µL, 10 mM). The reaction mixture was analysed by EPR spectroscopy.

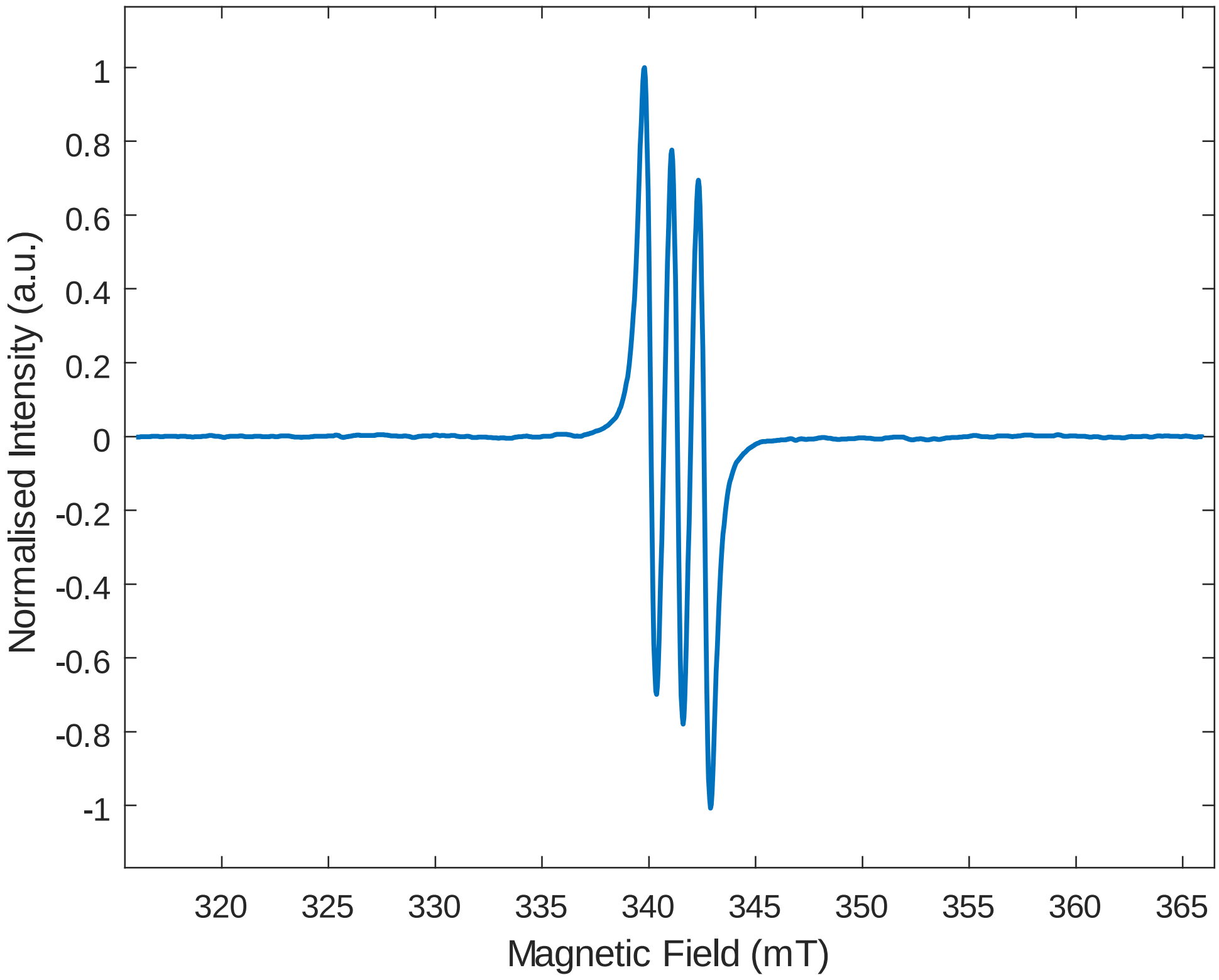

Figure S9. Continuous wave EPR spectrum of $[Fe(NO)(detc)_2]$ confirming NO contamination within the NO_2 cylinder. Experimental parameters: solvent: toluene, frequency: 9.740093 GHz, temperature: 298 K, modulation amplitude 4, 1 scan, receiver gain 30 dB, g = 2.038, A_N = 35.5 Hz

5. Synthesis and purification of NO_2

5.1 Experimental setup to synthesise NO_2

As commercially purchased NO_2 has NO as a contaminant, NO_2 was synthesised and purified. Nitric acid (65%, 0.1 mol, 10 mL) was added to a dropping funnel connected to a Schlenk-flask charged with elemental copper (6.3 g, 0.1 mol). Nitric acid was slowly added to the elemental copper generating NO_2 gas and $Cu(NO_3)_2$. The NO_2 gas, which is denser than air, flowed through the tubing and a three-way tap into a

flame-dried ampoule. Excess gas was quenched by bubbling it through a saturated solution of KOH. This procedure is in line with the literature synthesis of NO_2.[11]

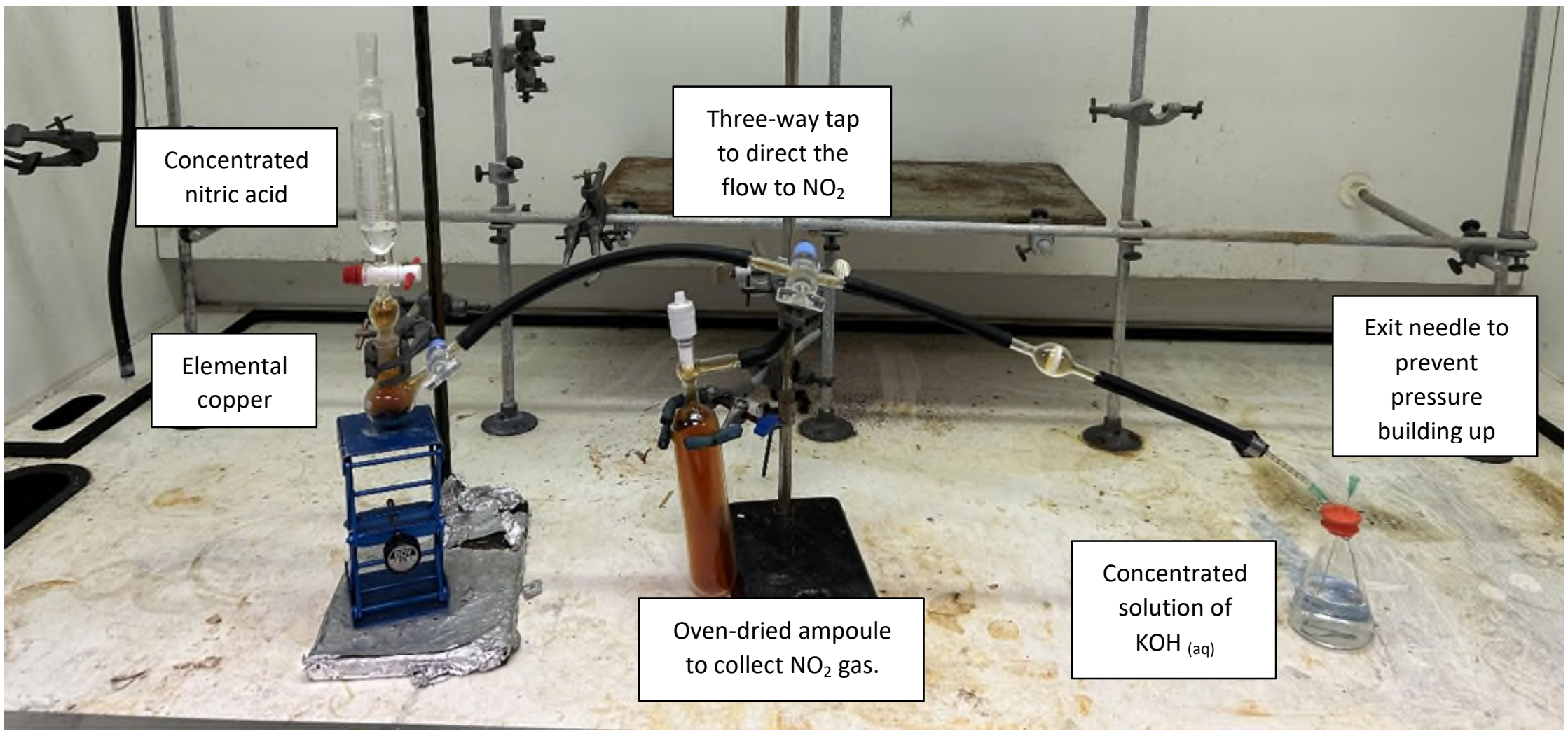

Figure S10. The experimental set-up used to generate and collect NO_2 in an ampoule.

5.2 EPR of NO_2

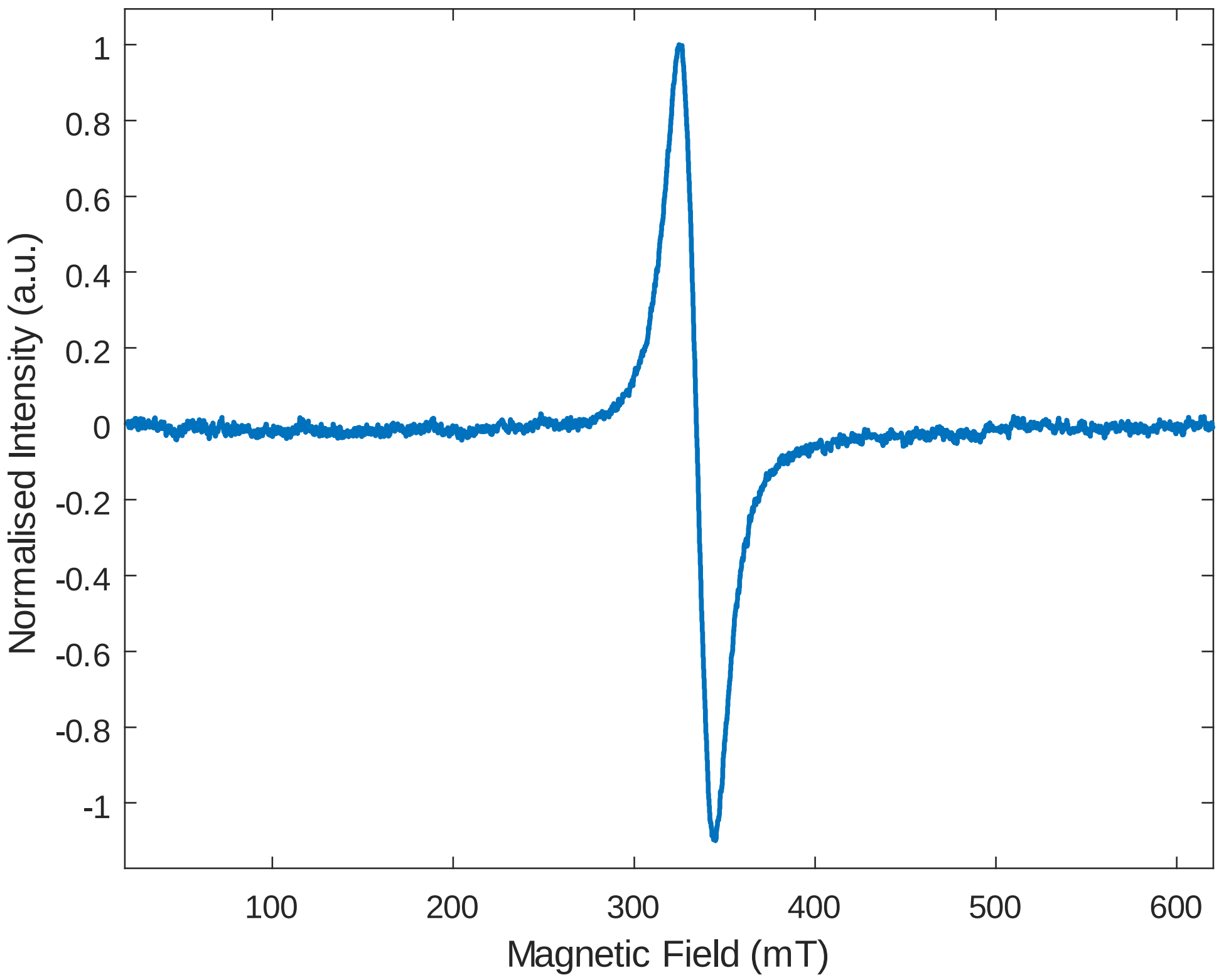

Figure S11. Continuous wave EPR spectra of NO_2. Experimental parameters: solvent: toluene, frequency: 9.372515 GHz, temperature: 298 K, modulation amplitude 4, 1 scan, receiver gain 30 dB, g = 2.001

5.3 Purification of NO_2

The ampoule of NO_2 was frozen at –20 °C and successively degassed by freeze-pump-thaw (approximately 15 times) to remove any concurrently generated NO (melting point –153 °C), until only trace NO could be detected by spin trapping using $Fe(detc)_3$.

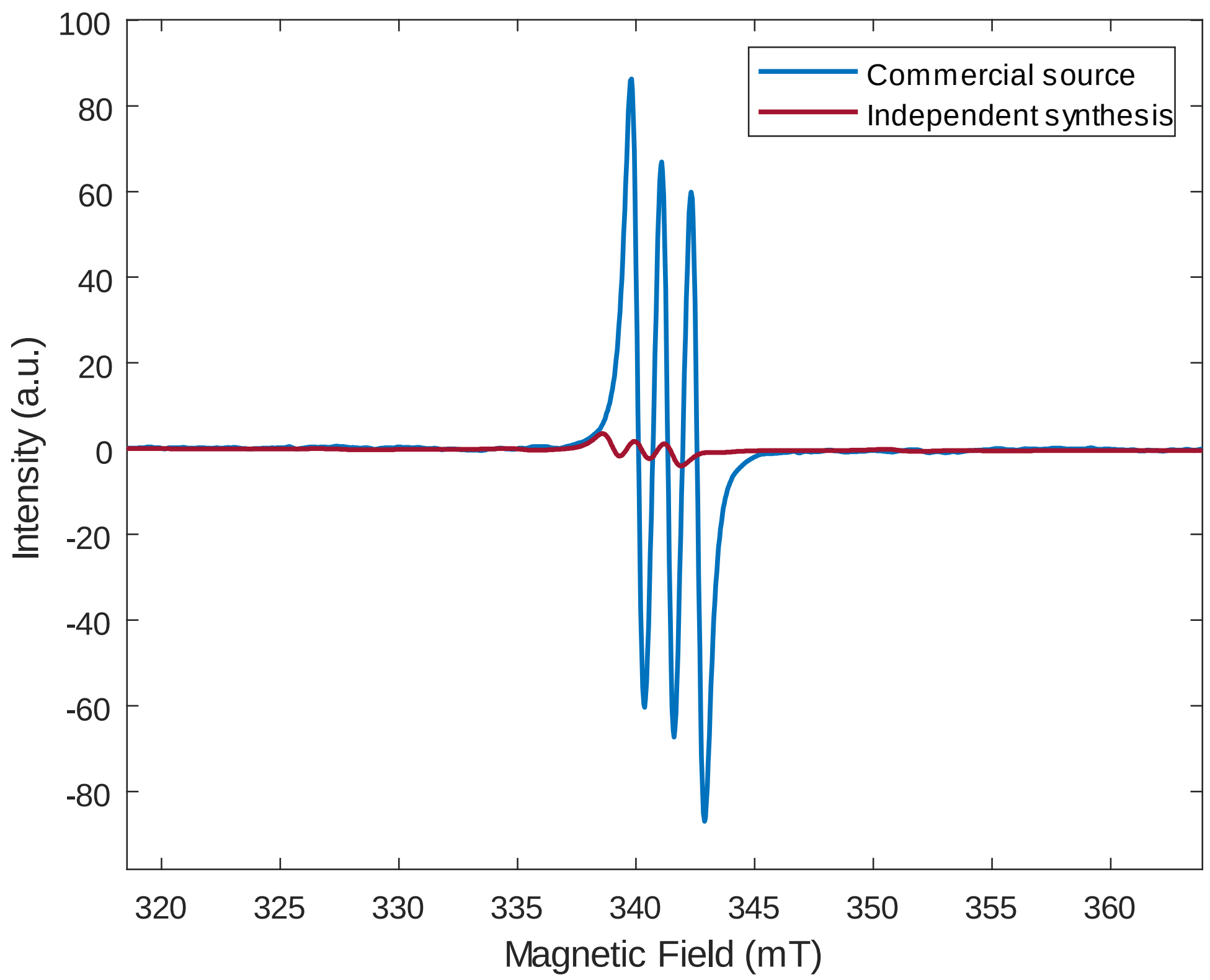

Figure S12. Continuous wave EPR spectra of $[Fe(NO)(detc)_2]$ from commercial canister showing NO contamination (blue) vs from independently synthesised NO_2 (red). Experimental parameters: solvent: toluene, frequency: 9.707564 GHz, temperature: 298 K, modulation amplitude 4.00 G, 1 scan, receiver gain 30 dB, g = 2.038, A_N = 35.5 Hz.

6 General Procedure for NO_2 (from independent synthesis, section 5) reduction with main group reducing agents

The general procedure below is given with HBpin. All the experiments in Table 1 in the manuscript were conducted as described below, by changing the reducing agent.

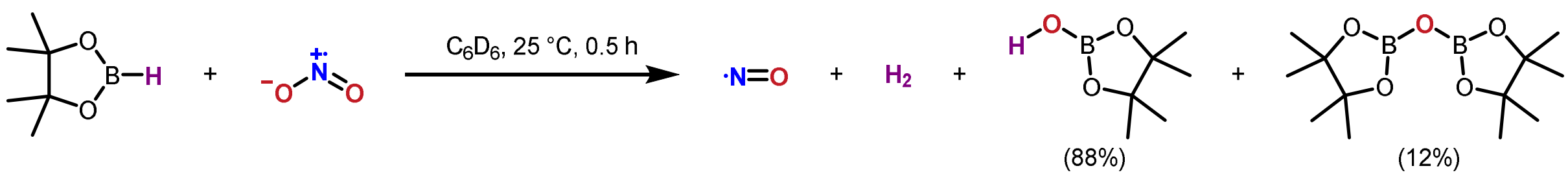

In an N_2 filled glovebox, HBpin (10.7 µL, 0.0735 mmol) was dissolved in C_6D_6 (0.7 mL) and added to a J. Young NMR tube (2.5 mL). The solution was degassed and pressurised with NO_2 (~1 atm, 25 °C). The J. Young NMR tube was inverted multiple times forming a light orange solution. Within 5 minutes the solution turned light green, and upon cooling to −196 °C, a bright blue solid was observed, consistent with the

formation of N_2O_3. The reaction mixture was analysed by ^1H and ^{11}B NMR spectroscopy. The yield of HOBpin (88%) and pinBOBpin (12%) was determined *in-situ* by relative integral comparison from the ^1H NMR spectrum using toluene as an internal standard.

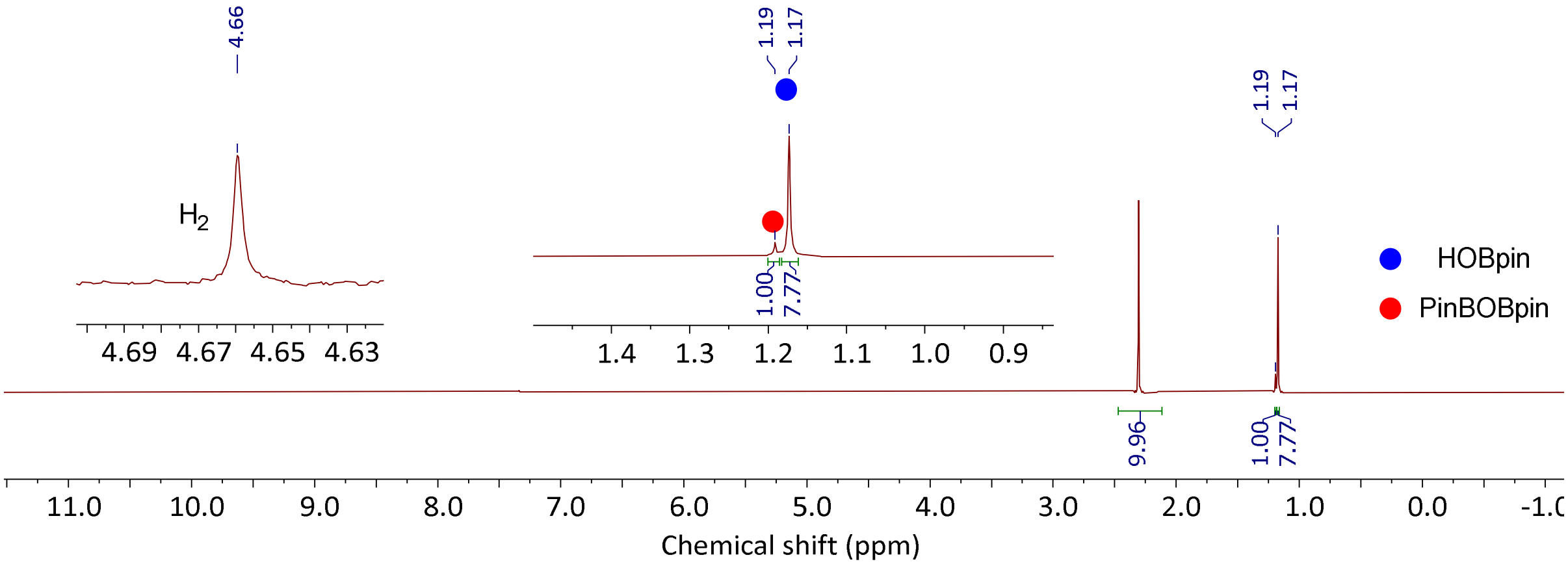

Figure S13. ^1H NMR spectrum (400 MHz, C_6D_6) of the hydroborative reduction of NO_2 by HBpin.

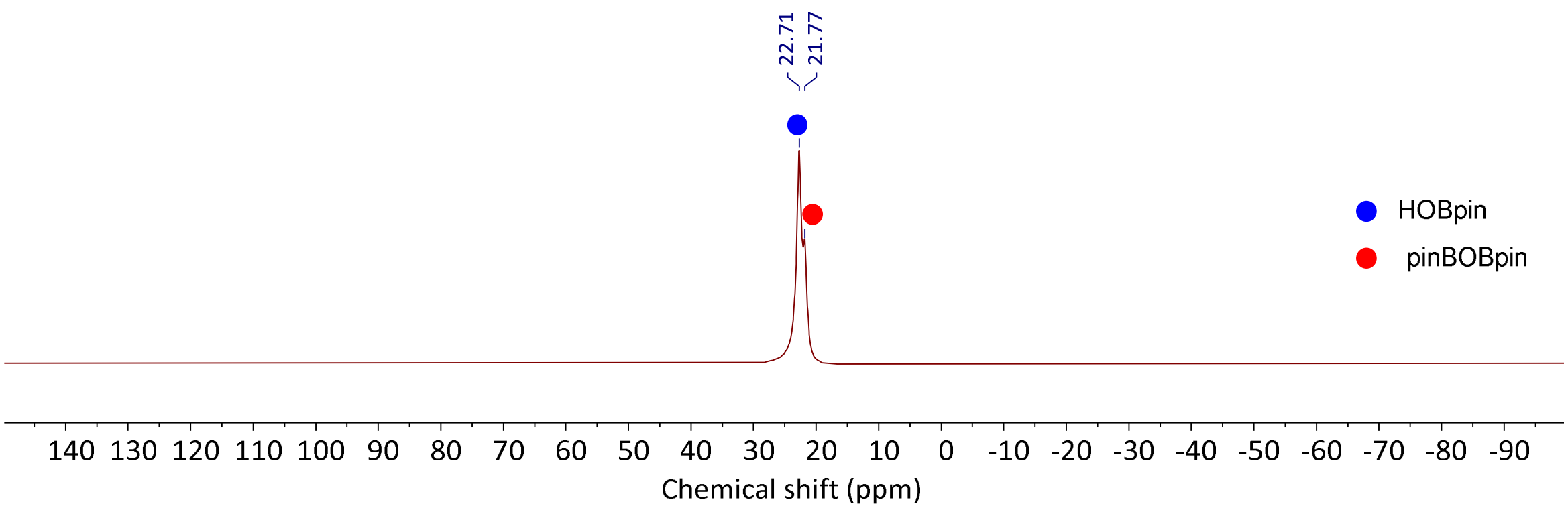

Figure S14. $^{11}B\{^1H\}$ NMR spectrum (128 MHz, C_6D_6) of the hydroborative reduction of NO_2 by HBpin.

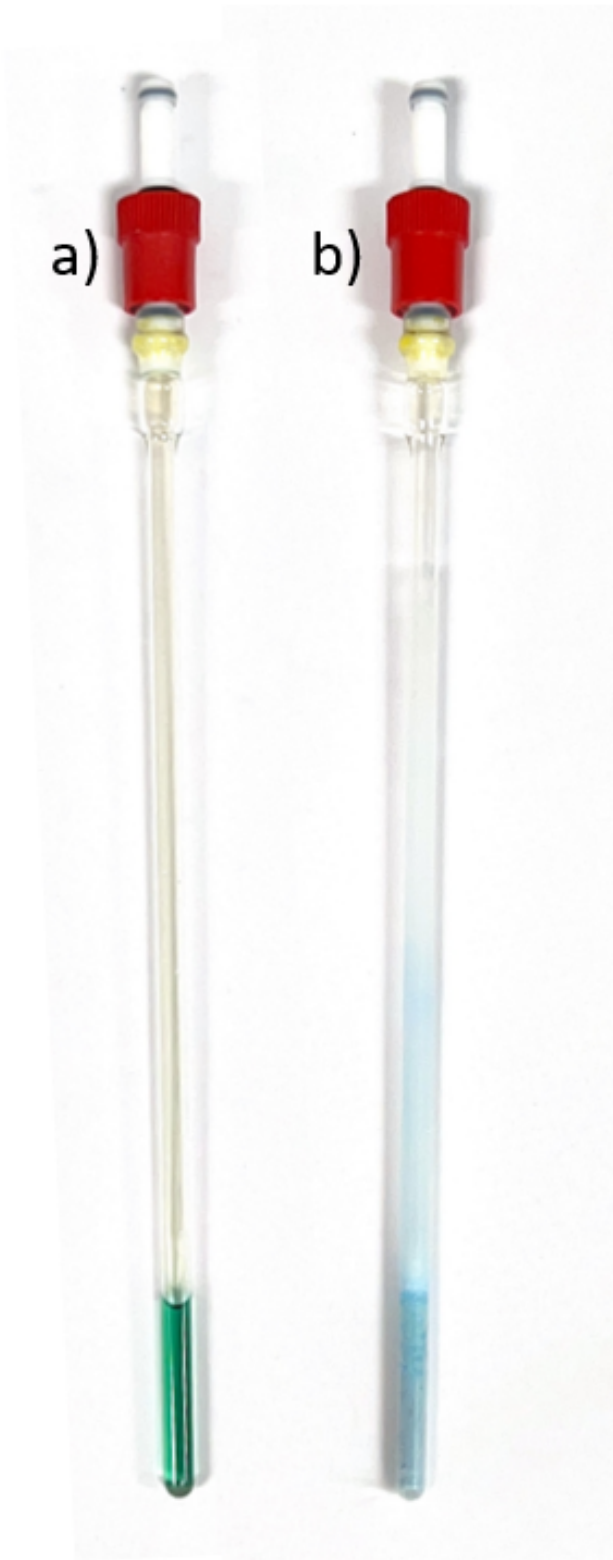

Figure S15. a) A light green reaction mixture of HBpin and excess NO_2 after 30 minutes at room temperature, b) reaction mixture of HBpin and NO_2 after 30 minutes at 77K, showing the distinct blue colour of N_2O_3.

6.1 Reduction of NO_2 to NO and subsequent spin trapping by $Fe(detc)_3$

The general procedure below is given with HBpin. All the experiments in Table 1 in the manuscript were conducted as described below, by changing the reducing agent.

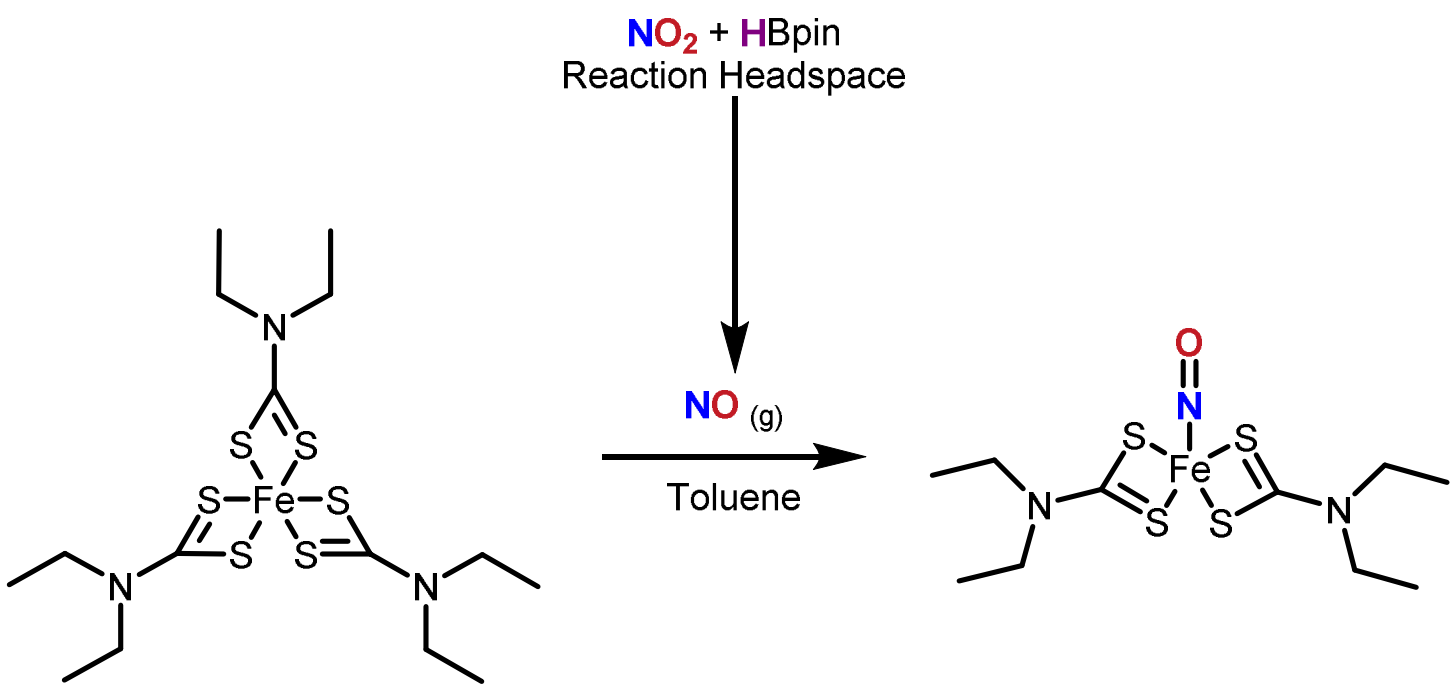

In an N_2 filled glovebox, HBpin (10.7 μL, 0.0735 mmol) was dissolved in C_6D_6 (0.7 mL) and added to a J. Young NMR tube (2.5 mL). The solution was degassed and the tube was pressurised with NO_2 (~1 atm, 25 °C). The J. Young NMR tube was inverted multiple times and allowed to react. After 30 minutes the reaction mixture was cooled to –20 °C to selectively freeze the excess NO_2. The NMR tube's headspace was then vacuum transferred to a degassed EPR tube charged with a toluene solution of

$Fe(detc)_3$ (200 µL, 10 mM). The reaction mixture was analysed by EPR spectroscopy, which showed the formation of a triplet (A_N = 35.5 Hz) at g = 2.038 which confirmed the formation of $Fe(NO)(detc)_2$, in agreement with literature values.[12]

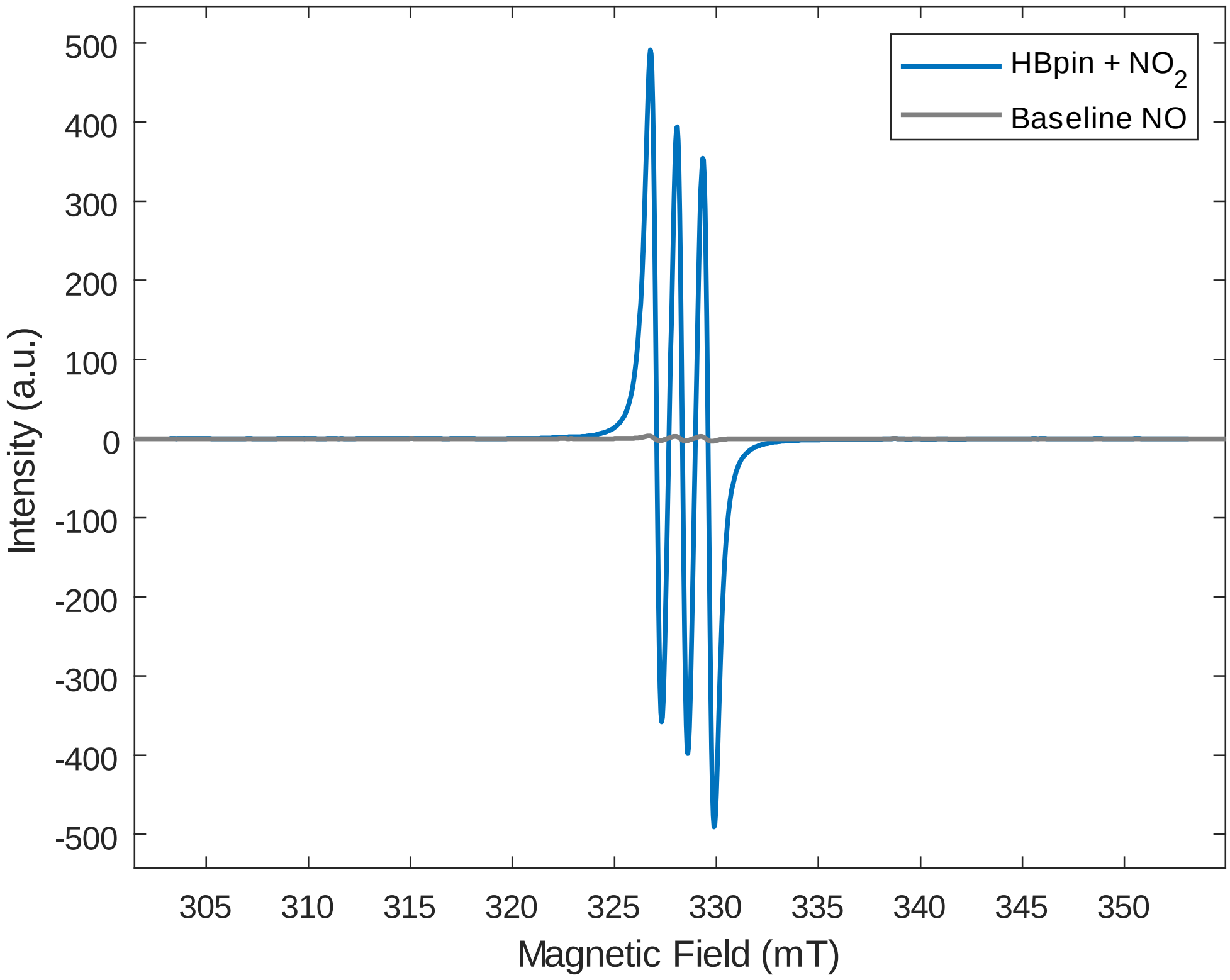

Figure S16. Continuous wave EPR spectra of $[Fe(NO)(detc)_2]$ generated from the reduction of NO_2 using HBpin. Experimental parameters: solvent: toluene, frequency: 9.367292 GHz, temperature: 298 K, modulation amplitude 4.00 G, 1 scan, receiver gain 30 dB, g = 2.038, A_N = 35.5

6.2 Reduction of NO_2 to NO and subsequent trapping by [T(OMe)PP]Co(II)

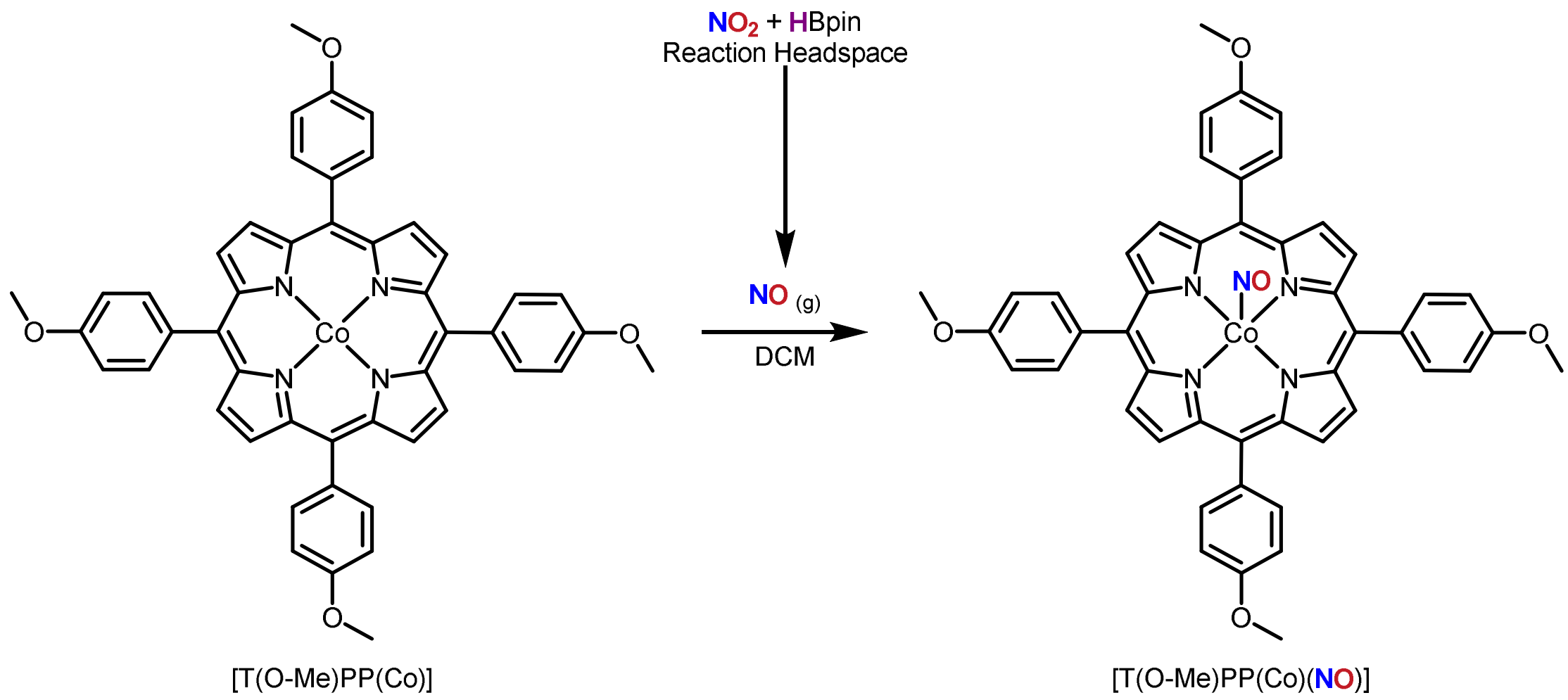

In an N_2 filled glovebox, HBpin (10.7 µL, 0.0735 mmol) was dissolved in C_6D_6 (0.7 mL) and added to a J. Young NMR tube (2.5 mL). The solution was degassed and the tube was pressurised with NO_2 (~1 atm, 25 °C). The J. Young NMR tube was inverted multiple times and allowed to react. After 30 minutes the reaction mixture was cooled to –20 °C to selectively freeze the excess NO_2. The NMR tube's headspace was then vacuum transferred to a degassed ampoule charged with a stirrer bar and a solution of [T(O-Me)PP(Co)] in DCM (58 mg, 0.0735 mmol, 1 equiv). The reaction mixture was left to stir for 12 hours. An aliquot (1 mL) was taken and the solvent was removed under reduced pressure. The resultant solid was dissolved in $CDCl_3$ and was analysed by ^{1}H NMR spectroscopy. [T(O-Me)PP(Co)(NO)] and [T(O-Me)PP(Co)] have markedly different ^{1}H NMR spectra, as shown in Figures S17 and S18. The resonance at δ 5.30 corresponds to [T(O-Me)PP]Co and the resonance at δ 4.06 corresponds to [T(O-Me)PP]Co(NO).

In previous work [T(O-Me)PP(Co)] has been employed to quantify the amount of NO generated. This experimental approach generally employs a double vial set-up,[13] where a solution of [T(O-Me)PP(Co)] traps the NO generated from a solid reagent in the inner vial. As NO_2 and NO are both gaseous, this approach was not appropriate for this chemistry. In cooling the reaction mixture to –20 °C, some NO converts to N_2O_3, as evidenced by the bright blue colour formed upon cooling. Therefore, it was not possible to transfer all the NO in the reaction headspace and ascertain an accurate NO conversion. However, analysis of the reaction mixtures by ^{1}H NMR spectroscopy does confirm the consumption of [T(O-Me)PP(Co)] and the formation of [T(O-Me)PP(Co)(NO)], shown in Figure S18, thus providing additional evidence of NO generation.

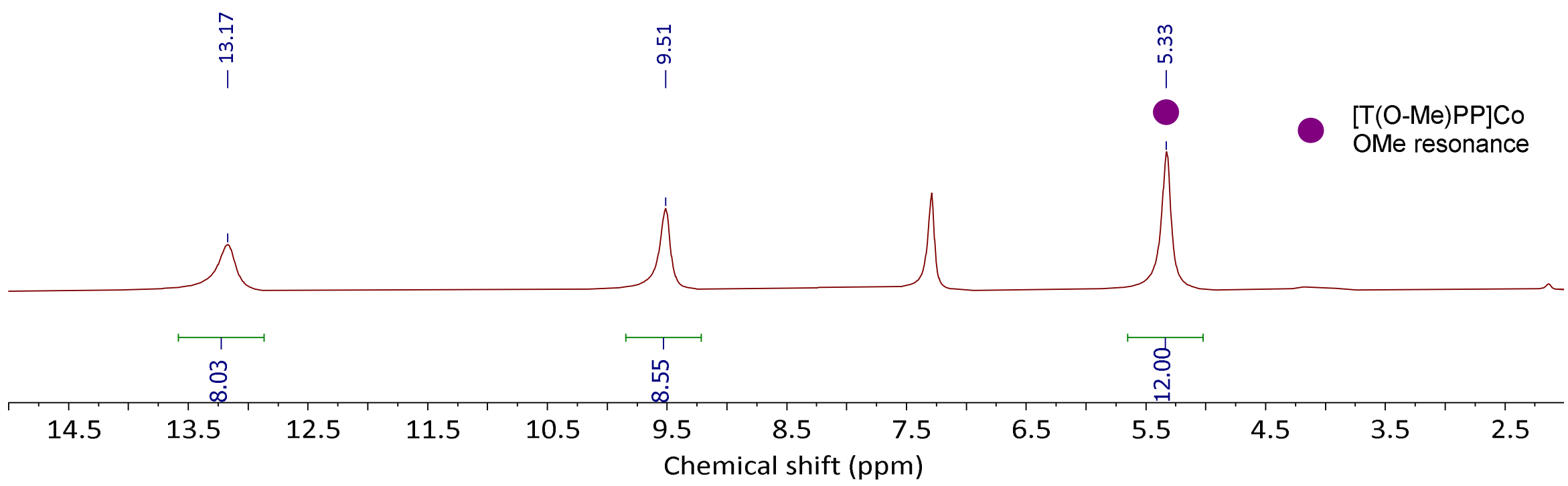

Figure S17. ^{1}H NMR spectrum (500 MHz, $CDCl_3$) of [T(O-Me)PP(Co)], highlighting the distinct methoxy resonance at δ 5.3 ppm.

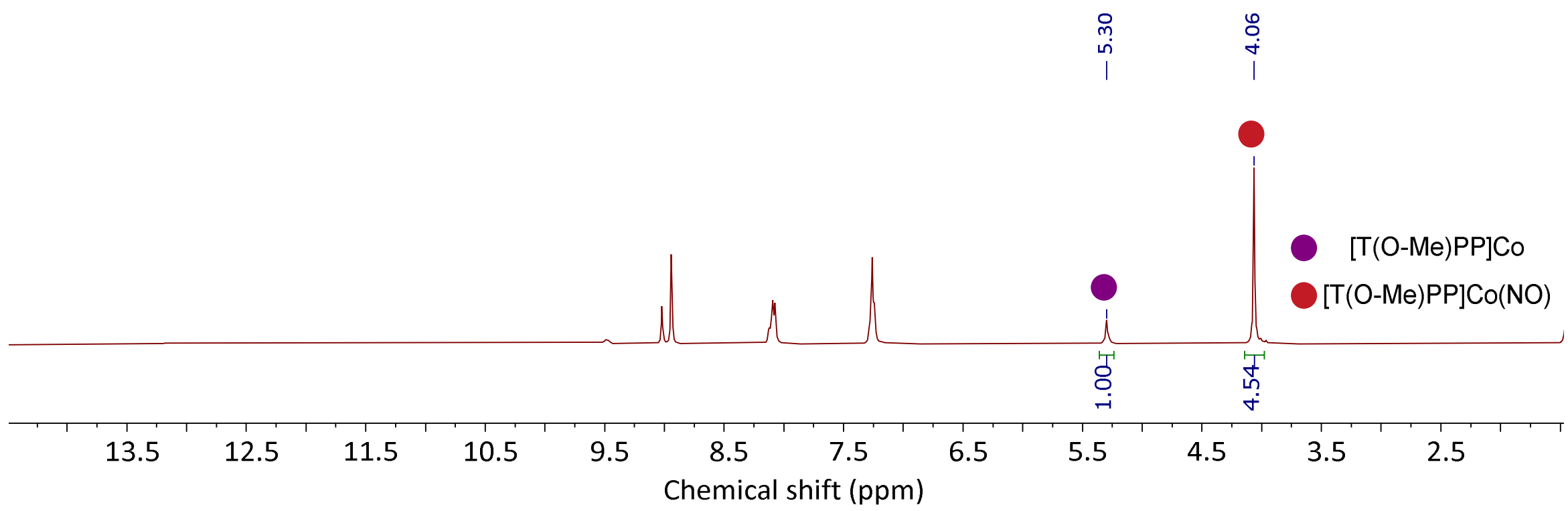

Figure S18. ^{1}H NMR spectrum (400 MHz, $CDCl_3$) of [T(O-Me)PP(Co)], showing the formation of [T(O-Me)PP(Co)(NO)] when exposed to the reaction headspace from the reduction of NO_2 using HBpin.

6.3 Characterisation data when $HSiEt_3$ was employed as a reductant

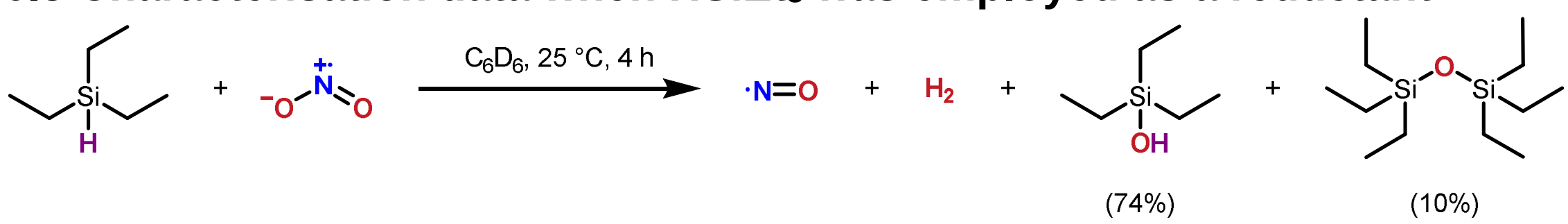

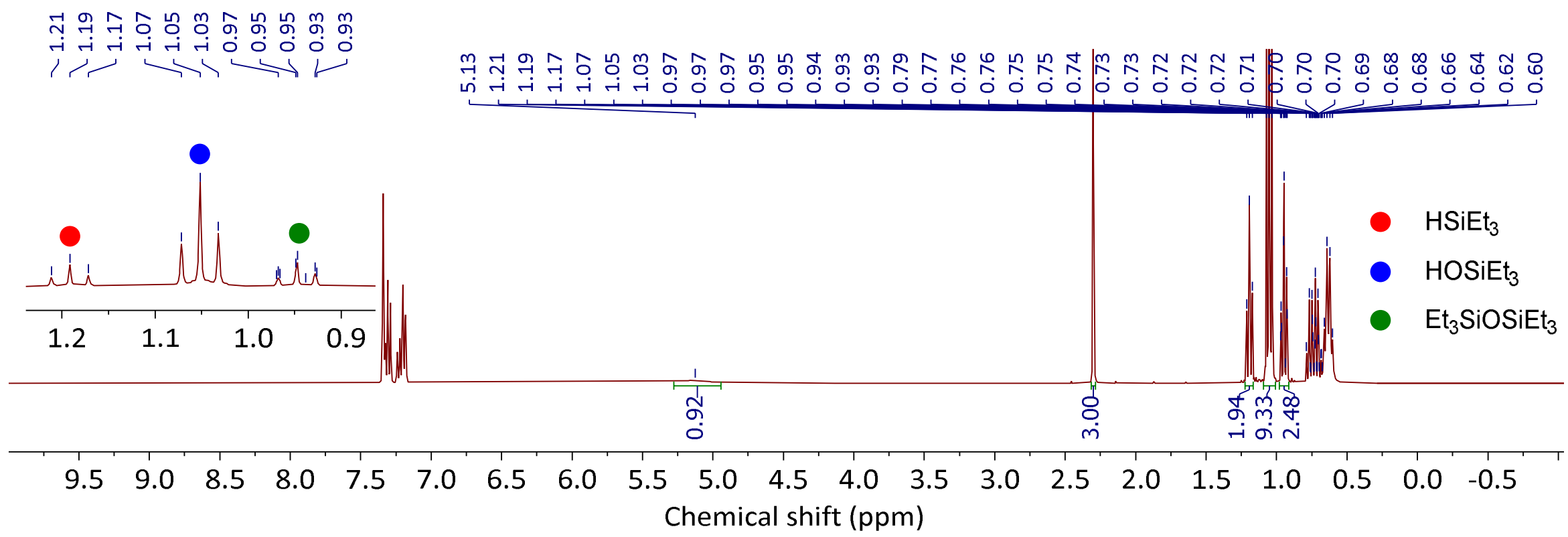

Figure S19. ^{1}H NMR spectrum (400 MHz, C_6D_6) showing the hydrosilylative reduction of NO_2

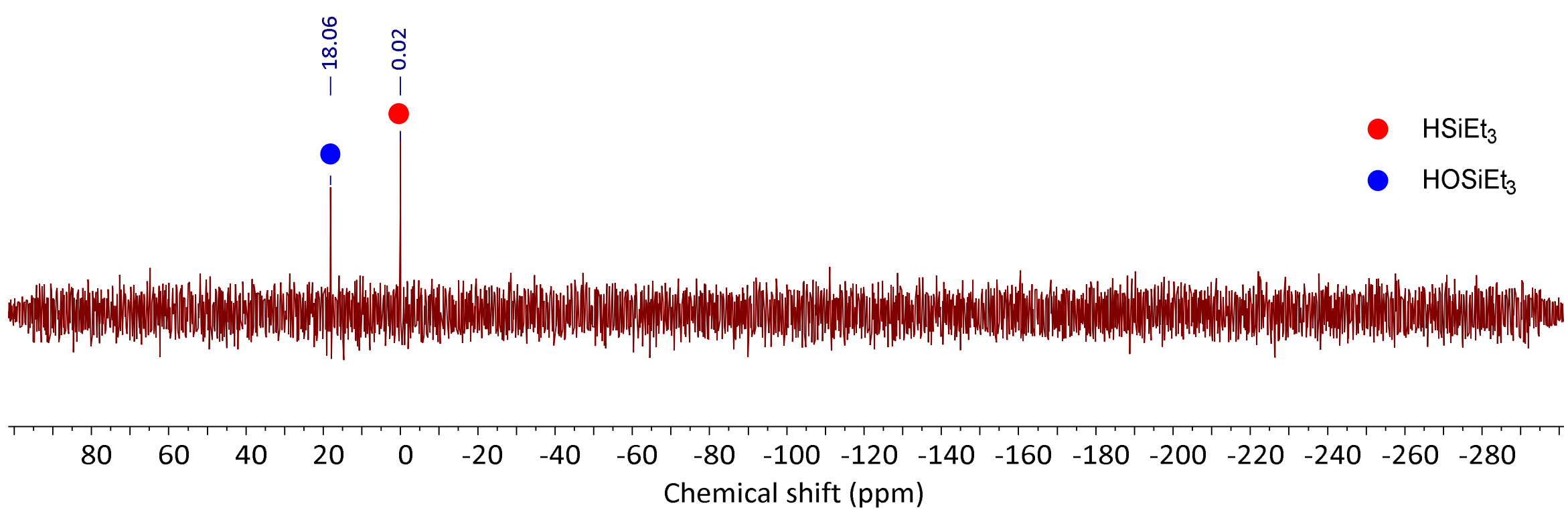

Figure S20. $^{29}Si\{^1H\}$ NMR spectrum (79.48 MHz, C_6D_6) showing the hydrosilylative reduction of NO_2.

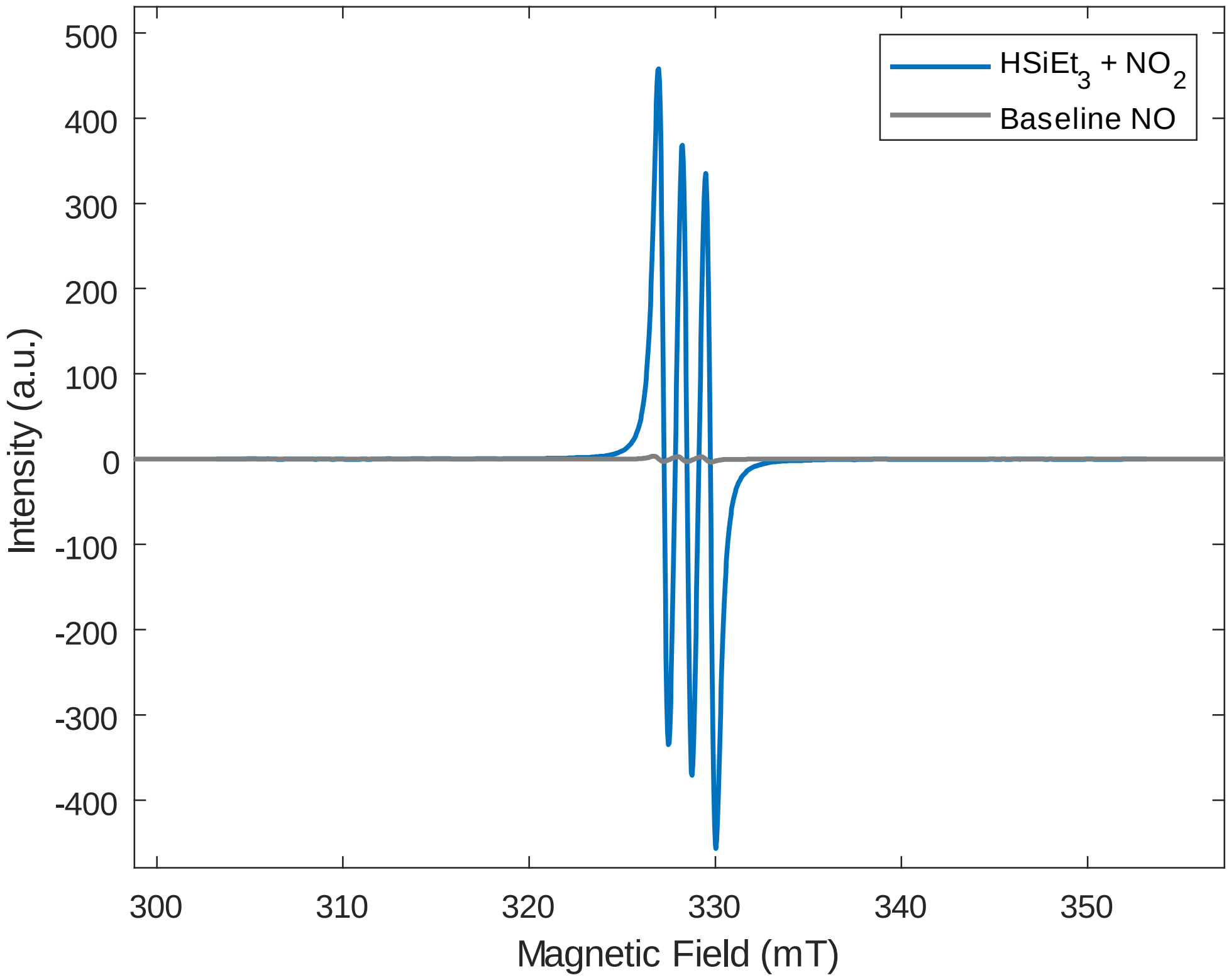

Figure S21. Continuous wave EPR spectra of [Fe(NO)(detc)$_2$] generated from the reduction of NO_2 using $HSiEt_3$. Experimental parameters: solvent: toluene, frequency: 9.373619 GHz, temperature: 298 K, modulation amplitude 4.00 G, 1 scan, receiver gain 30 dB, g = 2.038, A_N = 35.5 Hz.

6.4 Characterisation data when B_2pin_2 was employed as a reductant

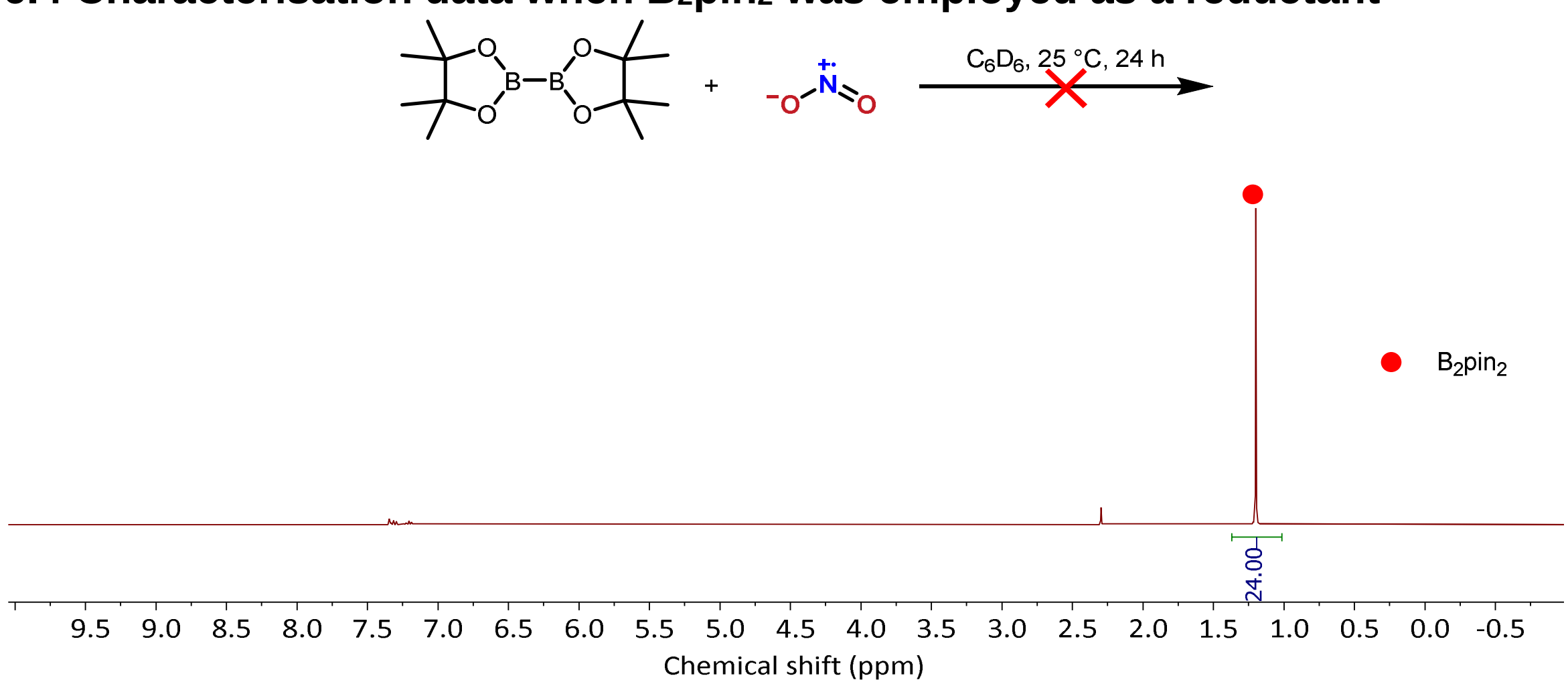

Figure S22. ^1H NMR spectrum (400 MHz, C_6D_6) showing unreacted B_2pin_2.

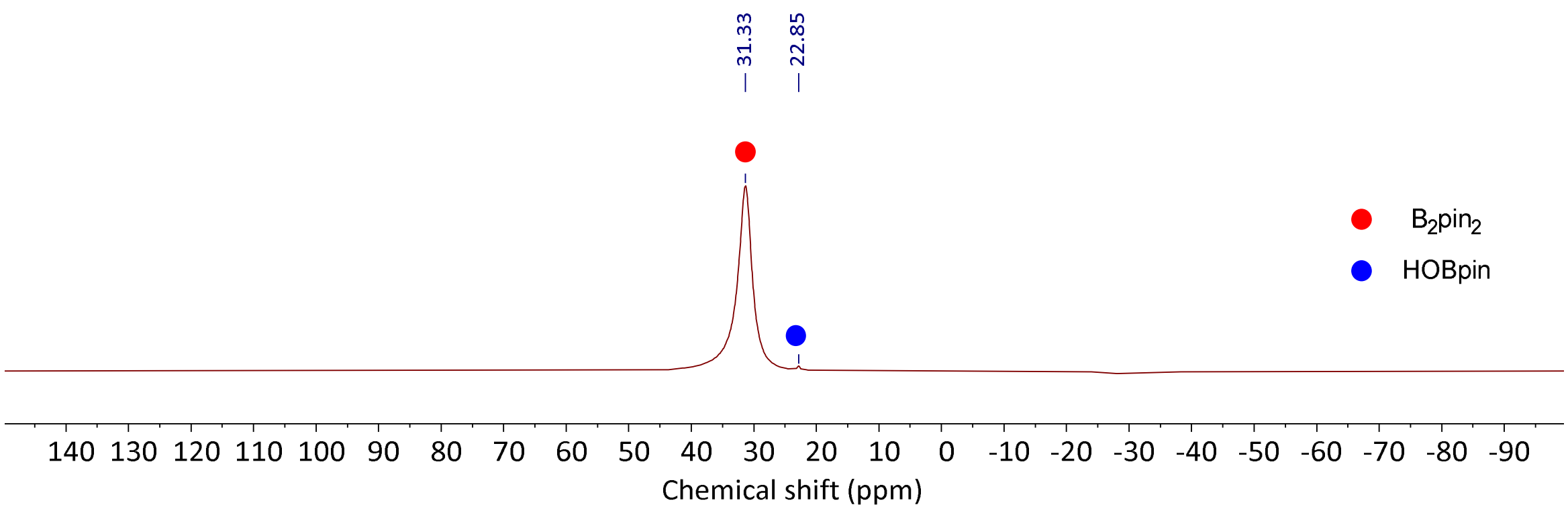

Figure S23. $^{11}B\{^{1}H\}$ NMR spectrum (128 MHz, C_6D_6) showing unreacted B_2pin_2.

6.5 Characterisation data when Si_2Me_6 was employed as a reductant

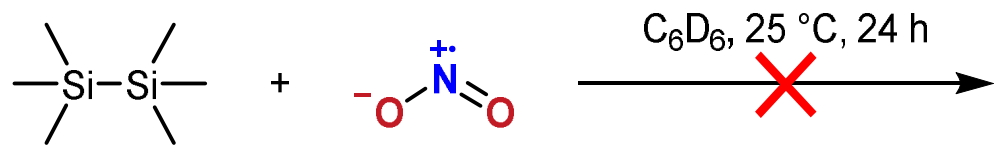

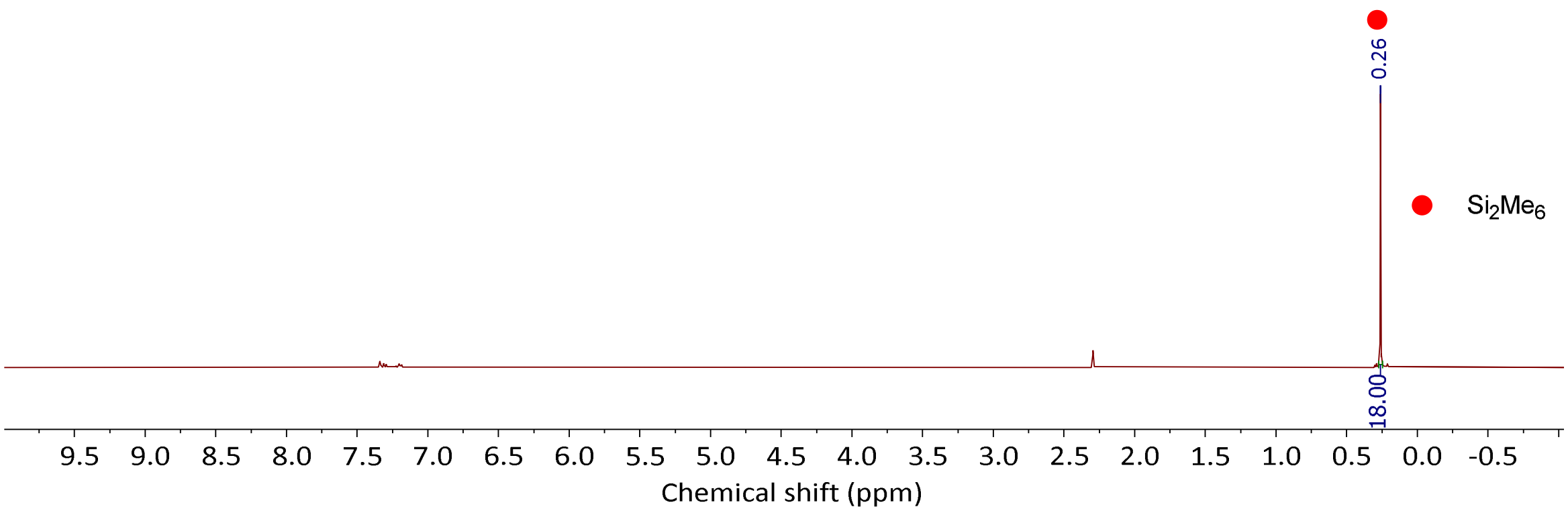

Figure S24. ^{1}H NMR spectrum (400 MHz, C_6D_6) showing unreacted Si_2Me_6.

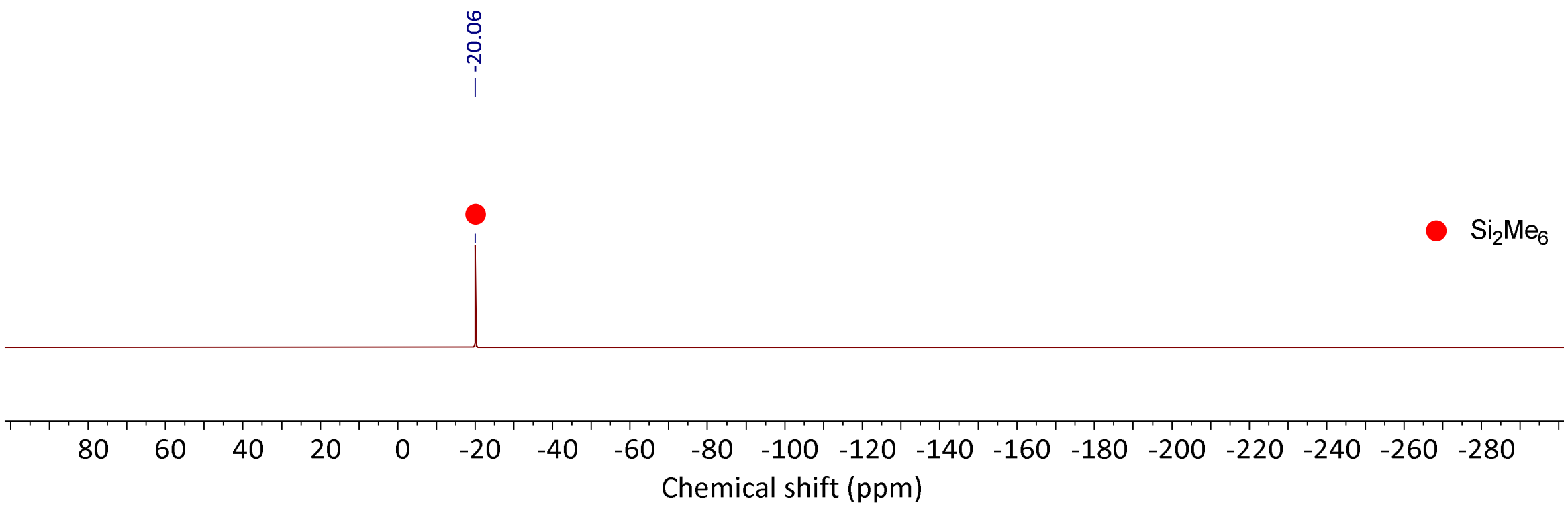

Figure S25. $^{29}Si\{^{1}H\}$ NMR spectrum (79.48 MHz, C_6D_6) showing unreacted Si_2Me_6.

6.6 Characterisation data when $HSiPh_3$ was employed as a reductant

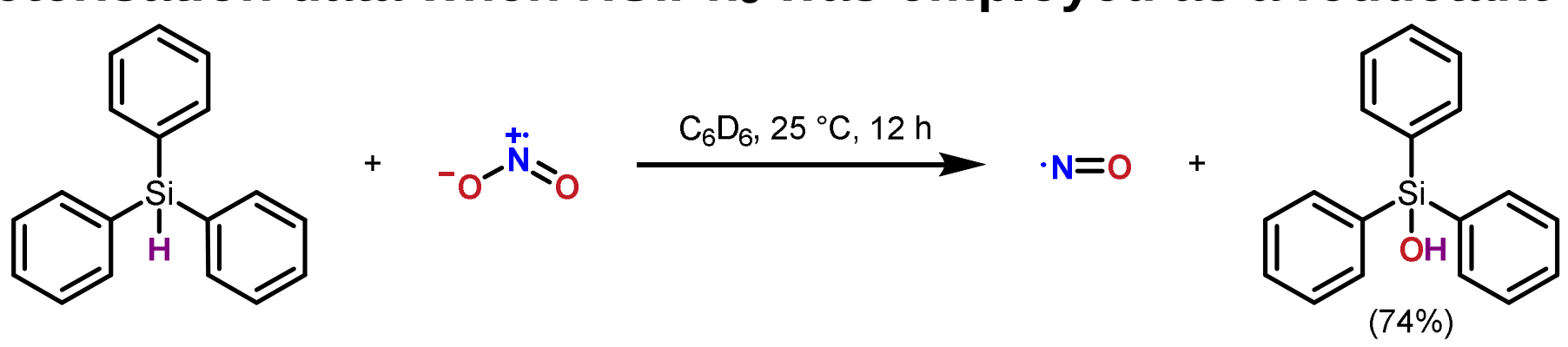

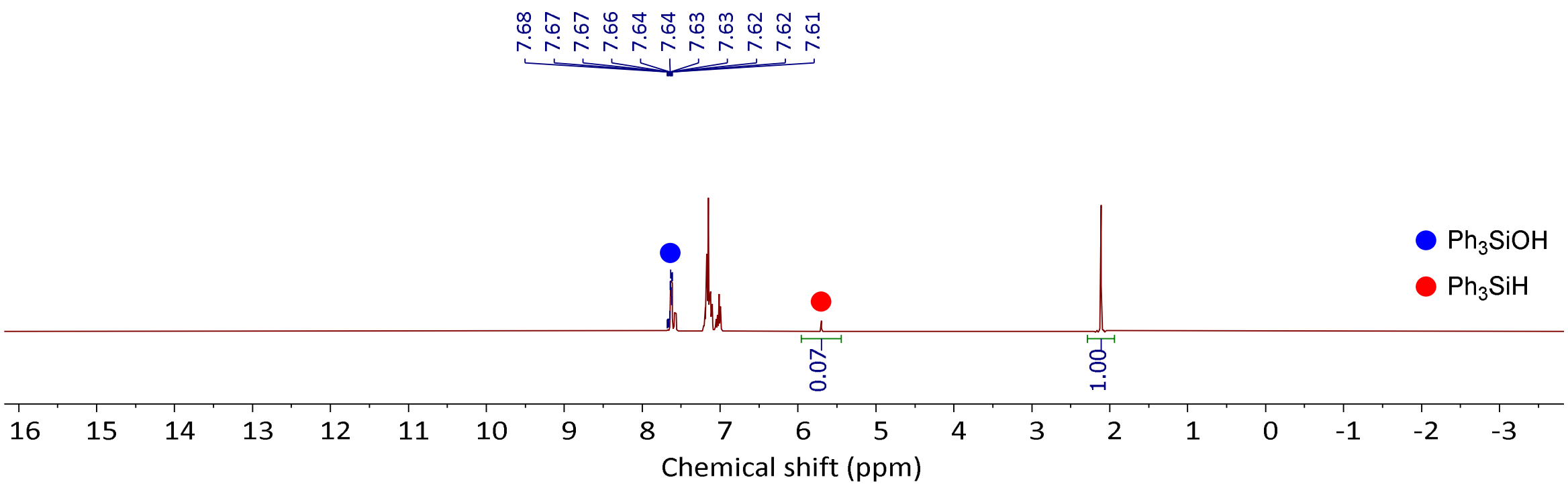

Figure S26. ^{1}H NMR spectrum (400 MHz, C_6D_6) showing the hydrosilylative reduction of NO_2

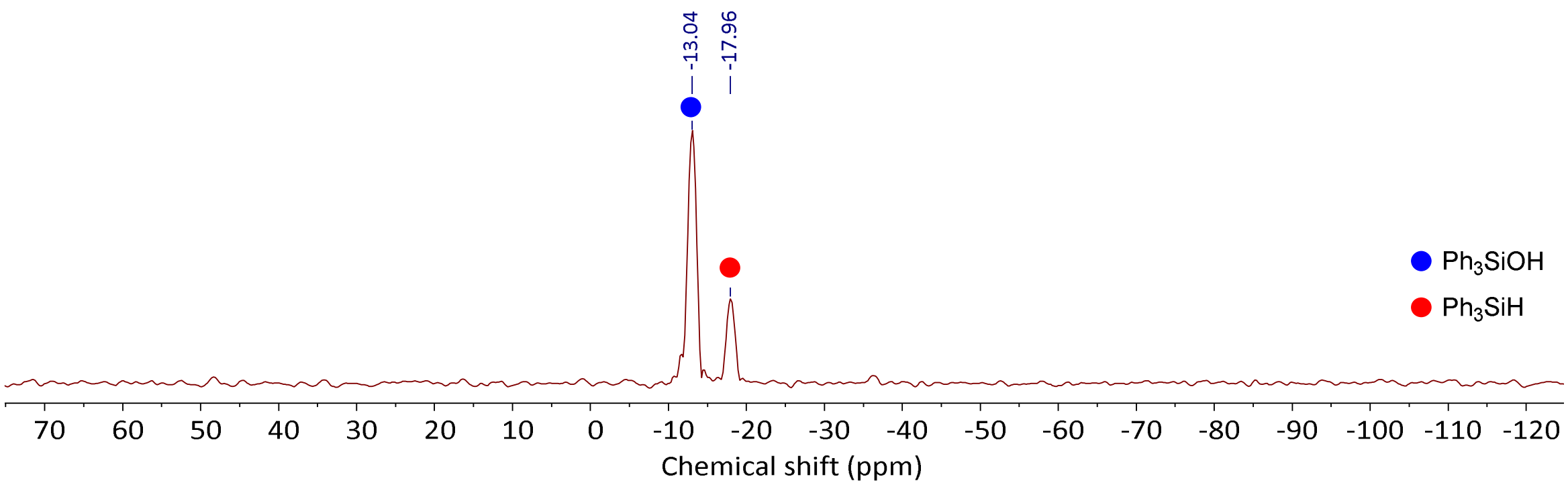

Figure S27. ^{29}Si{^{1}H} NMR spectrum (79.48 MHz, C_6D_6) showing the hydrosilylative reduction of NO_2

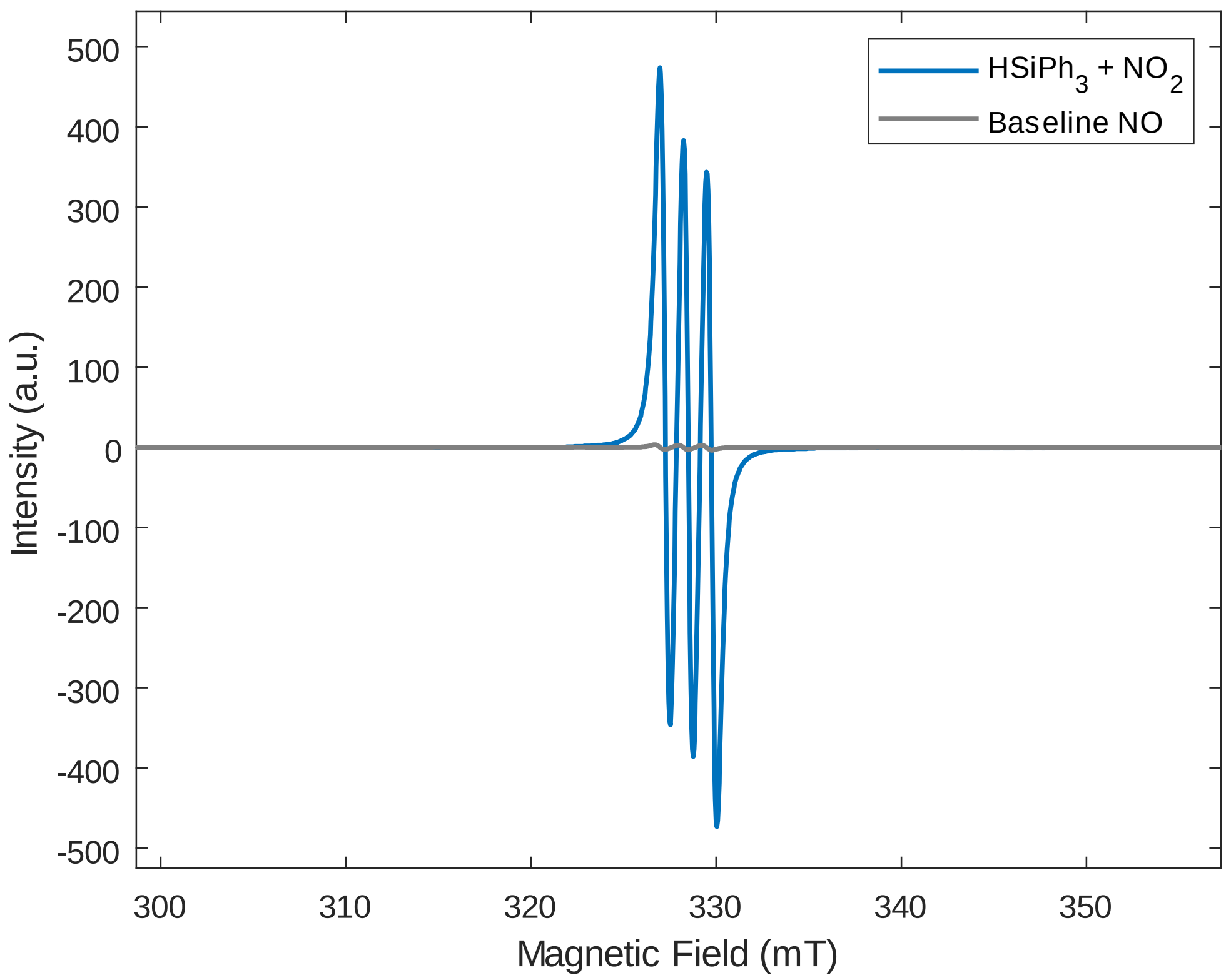

Figure S28. Continuous wave EPR spectra of [Fe(NO)(detc)$_2$] generated from the reduction of NO_2 using triphenylsilane. Experimental parameters: solvent: toluene, frequency: 9.31684 GHz, temperature: 298 K, modulation amplitude 4.00 G, 1 scan, receiver gain 30 dB, g = 2.038, A_N = 35.5 Hz.

6.7 Characterisation data when 1,4-cyclohexadiene was employed as a reductant

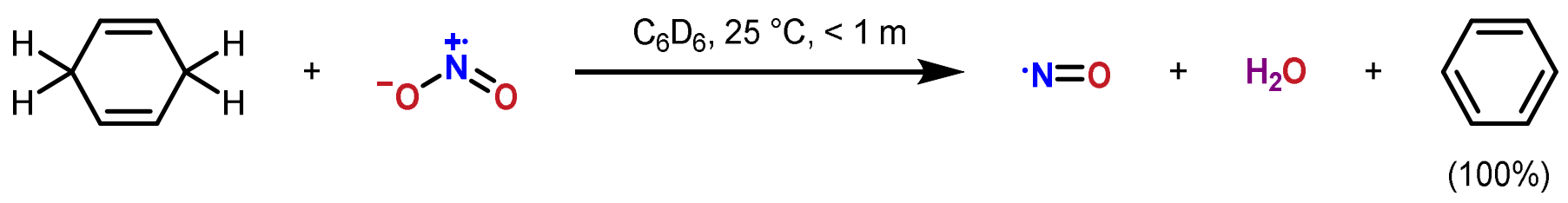

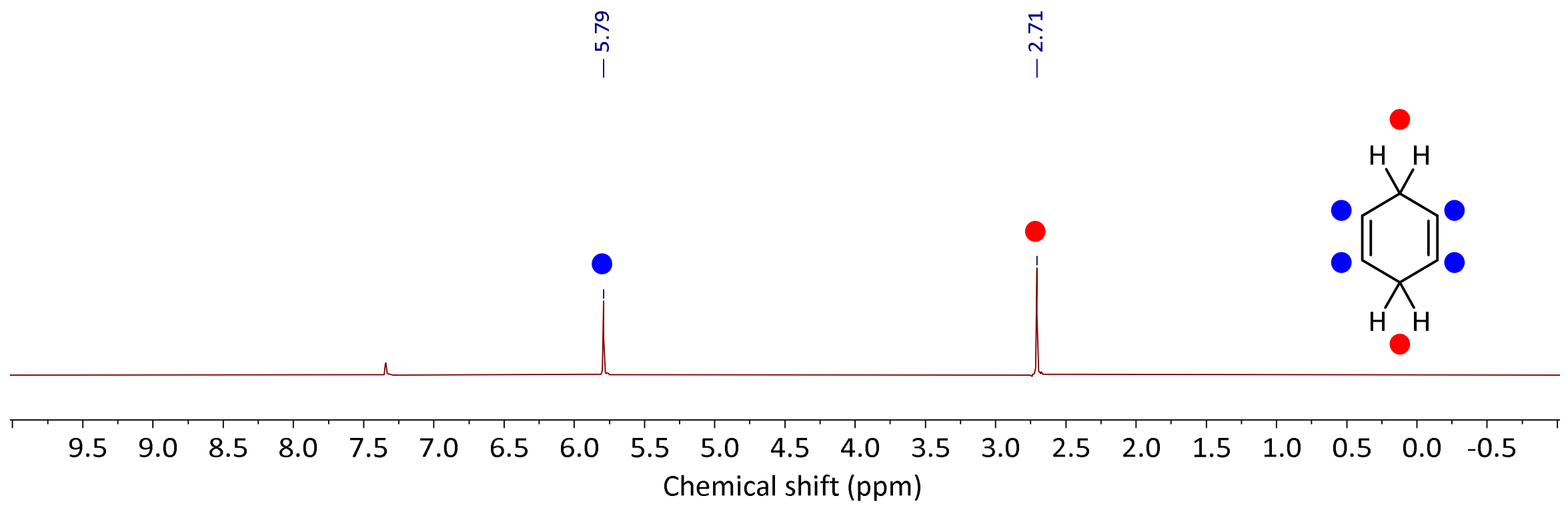

Figure S29. ^{1}H NMR spectrum (400 MHz, C_6D_6) showing 1,4-cyclohexadiene (CHD) before the addition of NO_2. Only trace C_6H_6 was observed, likely from the solvent.

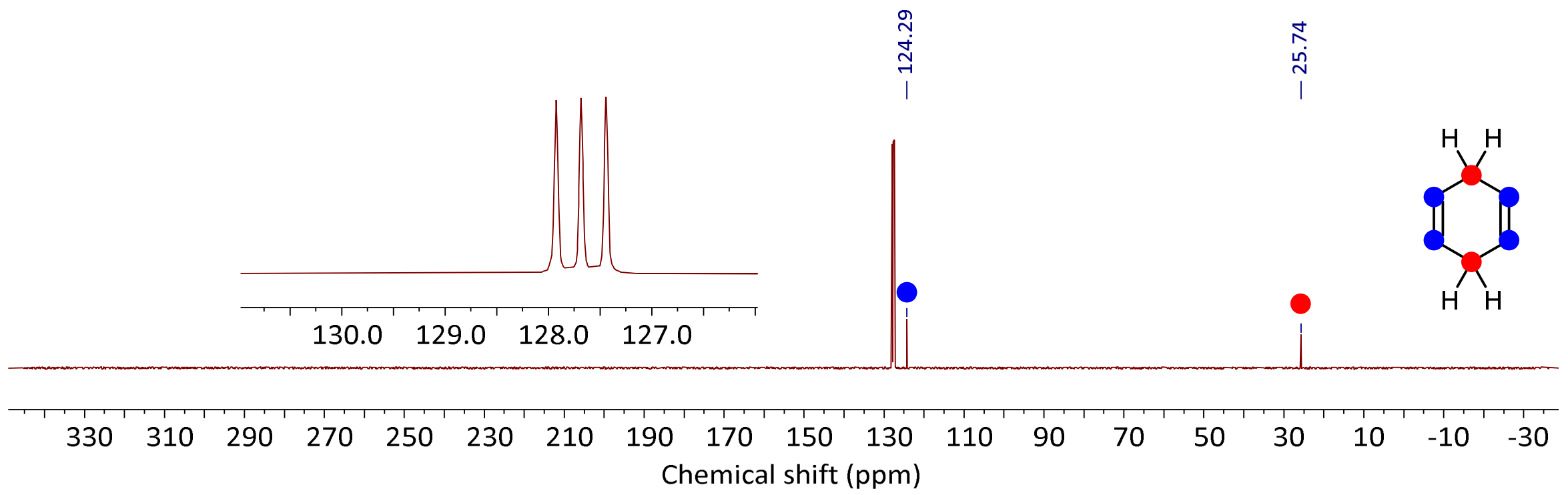

Figure S30. $^{13}C\{^1H\}$ NMR spectrum (101 MHz, C_6D_6) showing 1,4-cyclohexadiene (CHD) before the addition of NO_2.

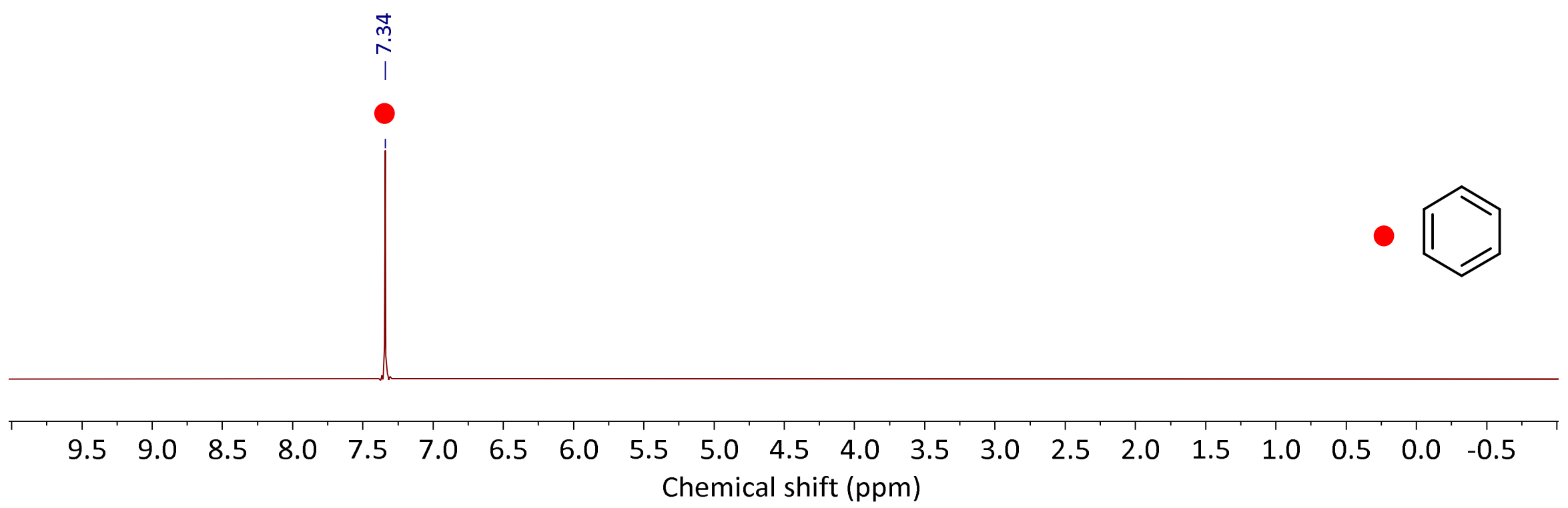

Figure S31. ^{1}H NMR spectrum (400 MHz, C_6D_6) showing the quantitative consumption of 1,4-cyclohexadiene to form benzene.

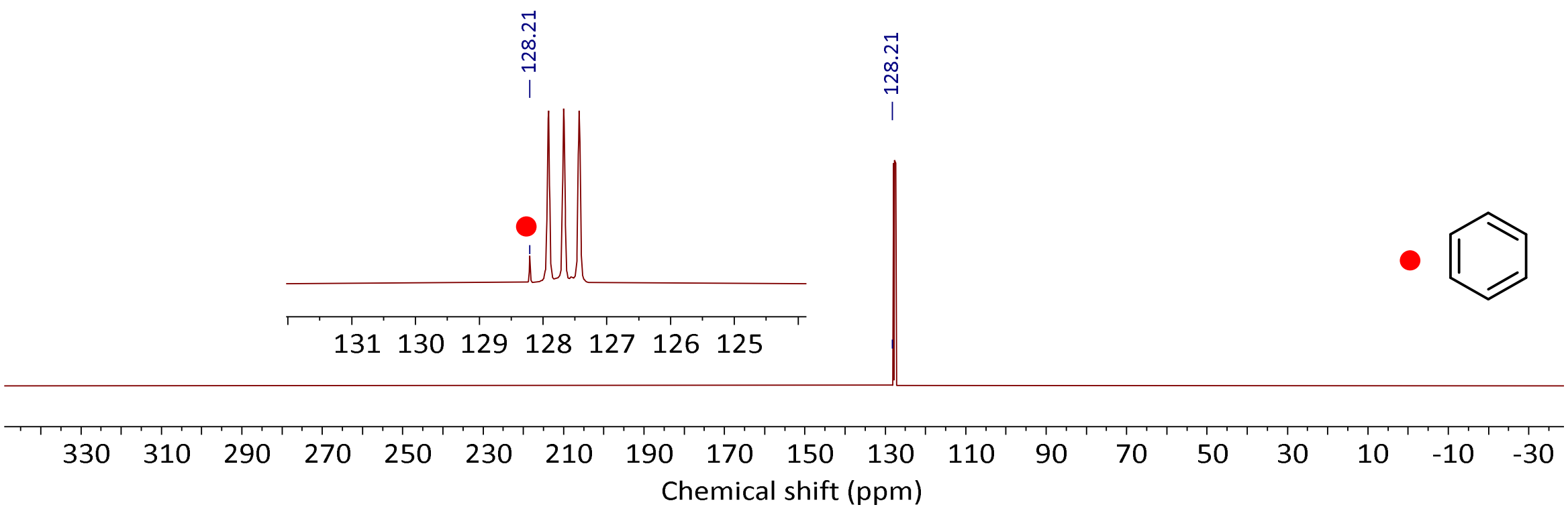

Figure S32. $^{13}C\{^{1}H\}$ NMR spectrum (101 MHz, C_6D_6) showing the quantitative consumption of 1,4-cyclohexadiene to form benzene.

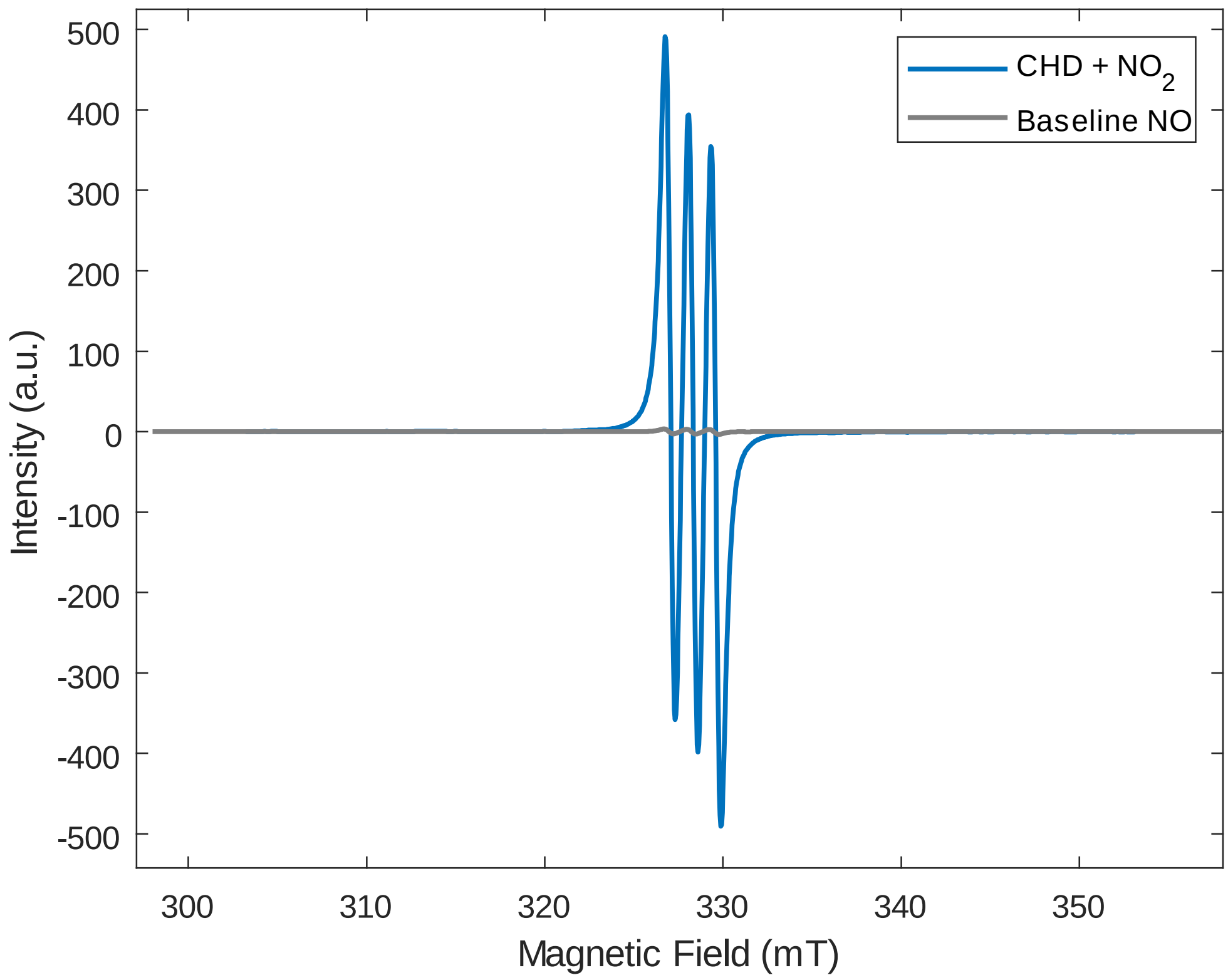

Figure S33. Continuous wave EPR spectra of $[Fe(NO)(detc)_2]$ generated from the reduction of NO_2 using 1,4-cyclohexadiene. Experimental parameters: solvent: toluene, frequency: 9.376491 GHz, temperature: 298 K, modulation amplitude 4.00 G, 1 scan, receiver gain 30 dB, g = 2.038, A_N = 35.5 Hz

6.8 Characterisation data when isopropyl alcohol was employed as a reductant

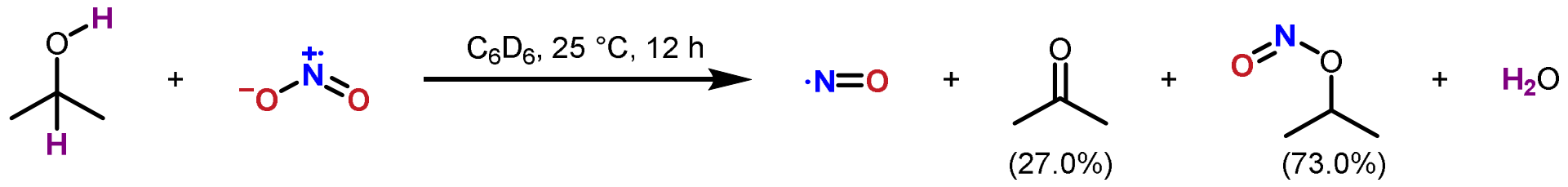

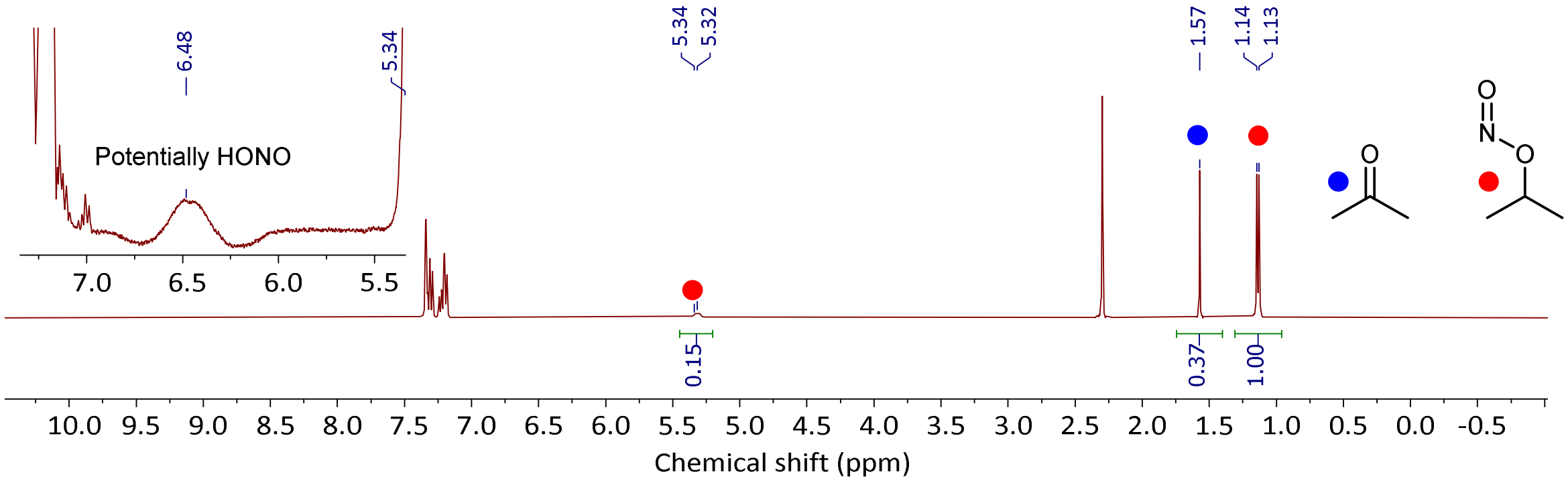

Figure S34. ^{1}H NMR spectrum (400 MHz, C_6D_6) showing the conversion of isopropyl alcohol to acetone, and the formation of isopropyl nitrite as an additional by-product.

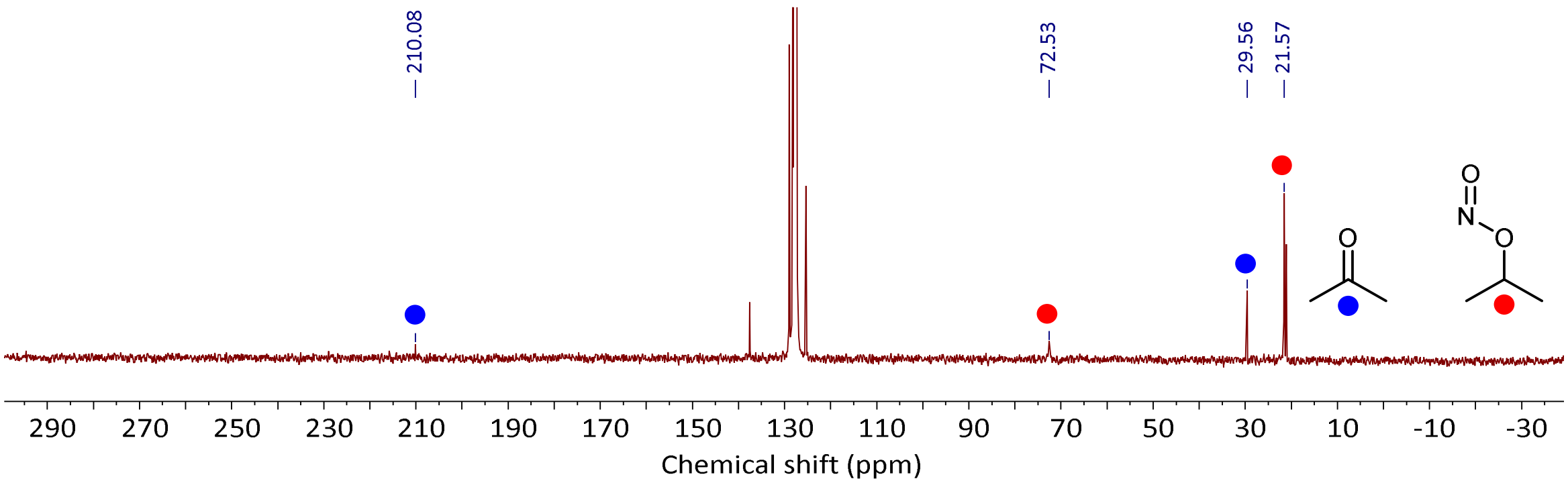

Figure S35. $^{13}C\{^{1}H\}$ NMR spectrum (101 MHz, C_6D_6) showing the conversion of isopropyl alcohol to acetone, and the formation of isopropyl nitrite as an additional

by-product.

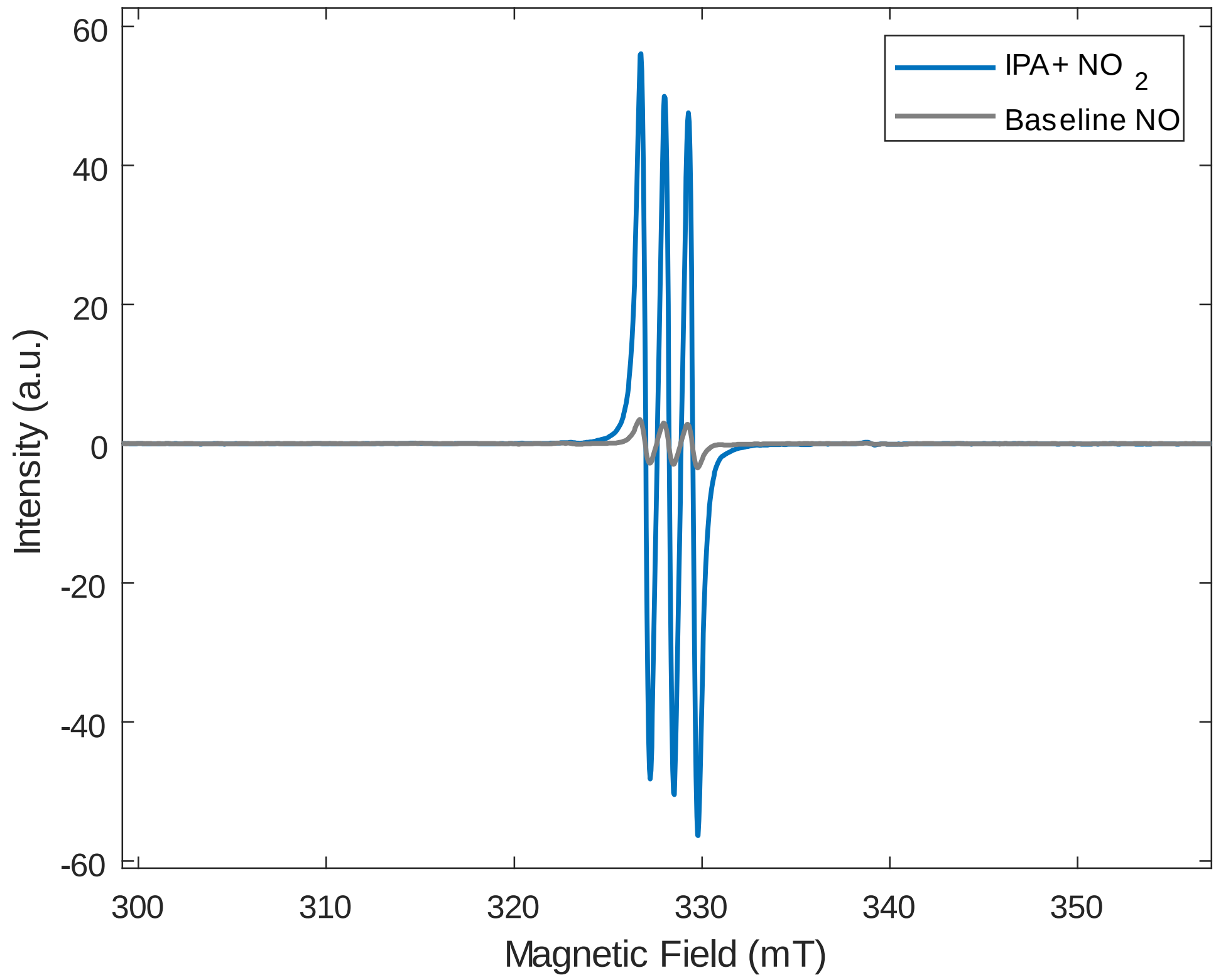

Figure S36. Continuous wave EPR spectra of [Fe(NO)(detc)$_2$] generated from the reduction of NO_2 using isopropyl alcohol. Experimental parameters: solvent: toluene, frequency: 9.366960 GHz, temperature: 298 K, modulation amplitude 4.00 G, 1 scan, receiver gain 30 dB, g = 2.038, A_N = 35.5 Hz.

7 ^{15}N-labelled Experiments

7.1 Synthesis and purification of ^{15}N-labelled NO_2

^{15}N-labelled NO_2 was synthesised and purified in an analogous manner to NO_2, described in section 5.

7.2 Reduction of 15NO_2 by HBpin

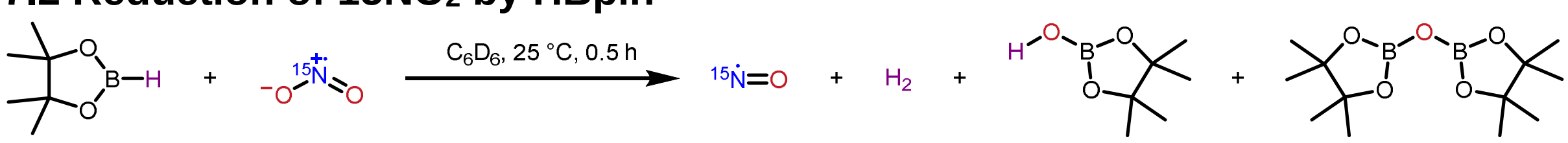

$^{15}NO_2$ was reduced using the method described in section 6. The reaction mixture was studied by low-temperature (–50 °C) ^{15}N{^{1}H} NMR which confirmed the presence of NO by the formation of the diamagnetic adduct N_2O_3. Spin trapping of ^{15}NO was carried out as described in section 6.1.

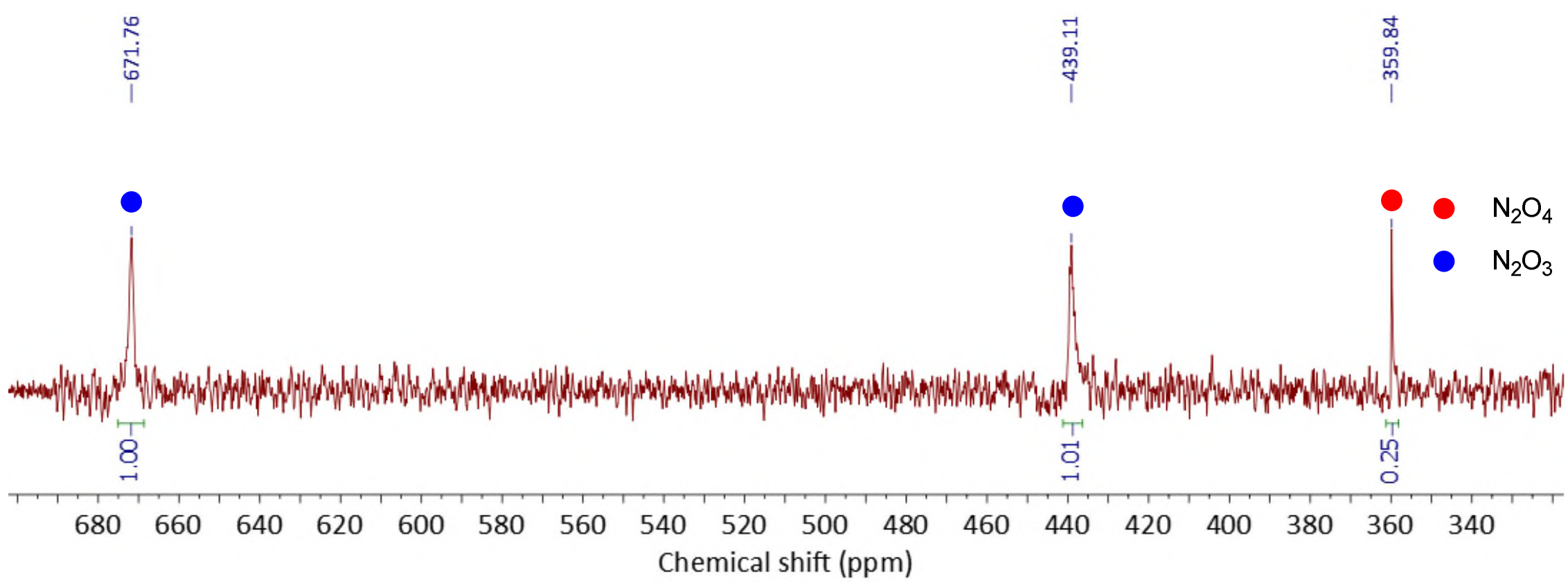

Figure S37. $^{15}N\{^1H\}$ NMR spectrum (51 MHz, C_7H_8) of the hydroborative reduction of $^{15}NO_2$ at –50 °C after 10 minutes.

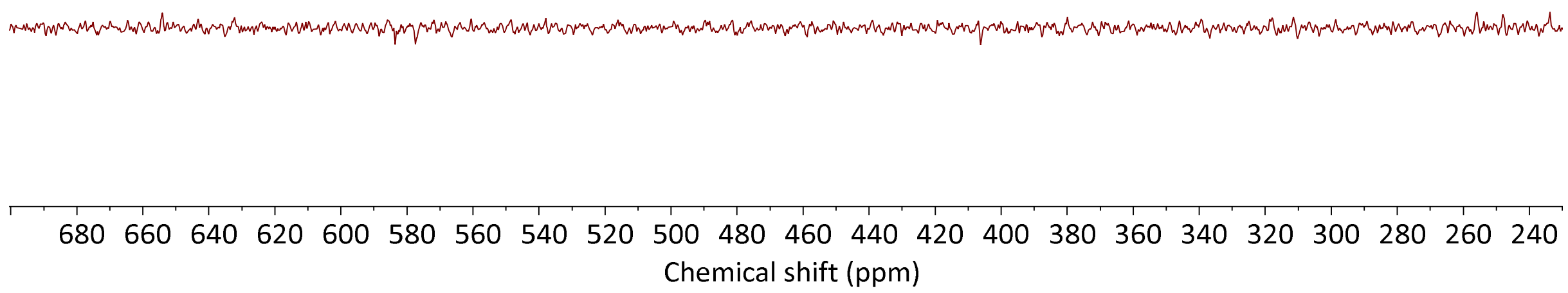

Figure S38. $^{15}N\{^1H\}$ NMR spectrum (51 MHz, C_7D_8) of hydroborative reduction of $^{15}NO_2$ at room temperature after 10 minutes. N_2O_3 and N_2O_4 are not stable at room temperature, and dissociate back into radicals, hence the lack of resonances.[14]

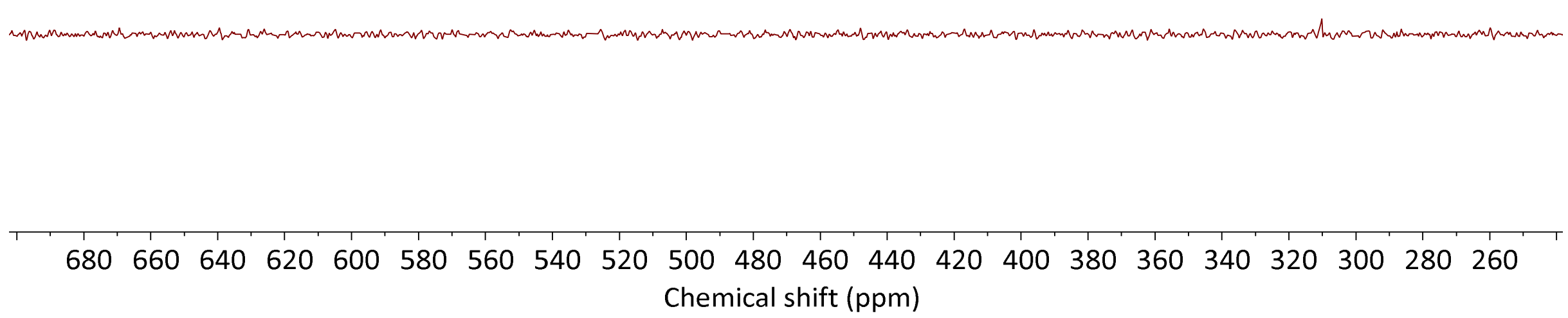

Figure S39. $^{15}N\{^1H\}$ NMR spectrum (51 MHz, C_7H_8) of the hydroboration of $^{15}NO_2$ at –50 °C after 30 minutes, indicating the complete hydroborative reduction of $^{15}NO_2$ to ^{15}NO. N_2O_2 is not stable at –50 °C, and was therefore not observed.[15]

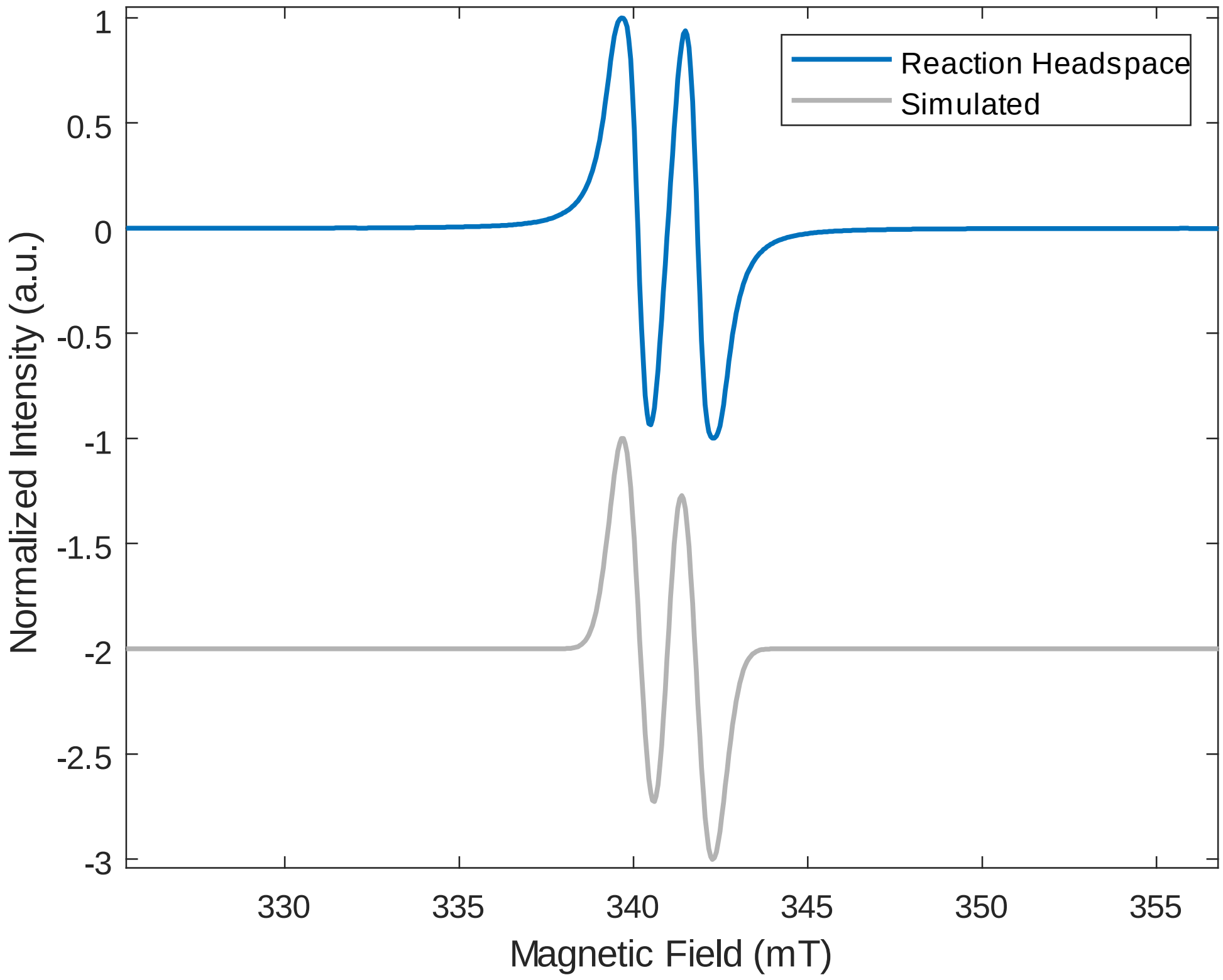

Figure S40. Continuous wave EPR spectra of [Fe(^{15}NO)(detc)$_2$] confirming the formation of ^{15}NO. Simulation (grey) x1 A_{15N} = 45.5 Hz. Experimental parameters: solvent: toluene, frequency: 9.739740 GHz, temperature: 298 K, modulation amplitude 4, 1 scan, receiver gain 30 dB, g = 2.04

8 Mechanistic Investigations

8.1 Reaction of NO_2 with Excess HBpin

In an N_2 filled glovebox, HBpin (53.5 μL, 0.3675 mmol) was dissolved in toluene (0.2 mL) and added to a J. Young EPR tube. The solution was degassed and pressurised with NO_2 (~1 atm, 25 °C). The EPR tube was inverted multiple times forming a light orange solution. Within 5 minutes the solution turned light green, then gradually turned yellow. After 30 minutes, upon cooling to −196 °C, the reaction mixture formed a pale-yellow solid, consistent with the complete consumption of NO_2. This was confirmed by EPR spectroscopy, as no resonances could be detected.

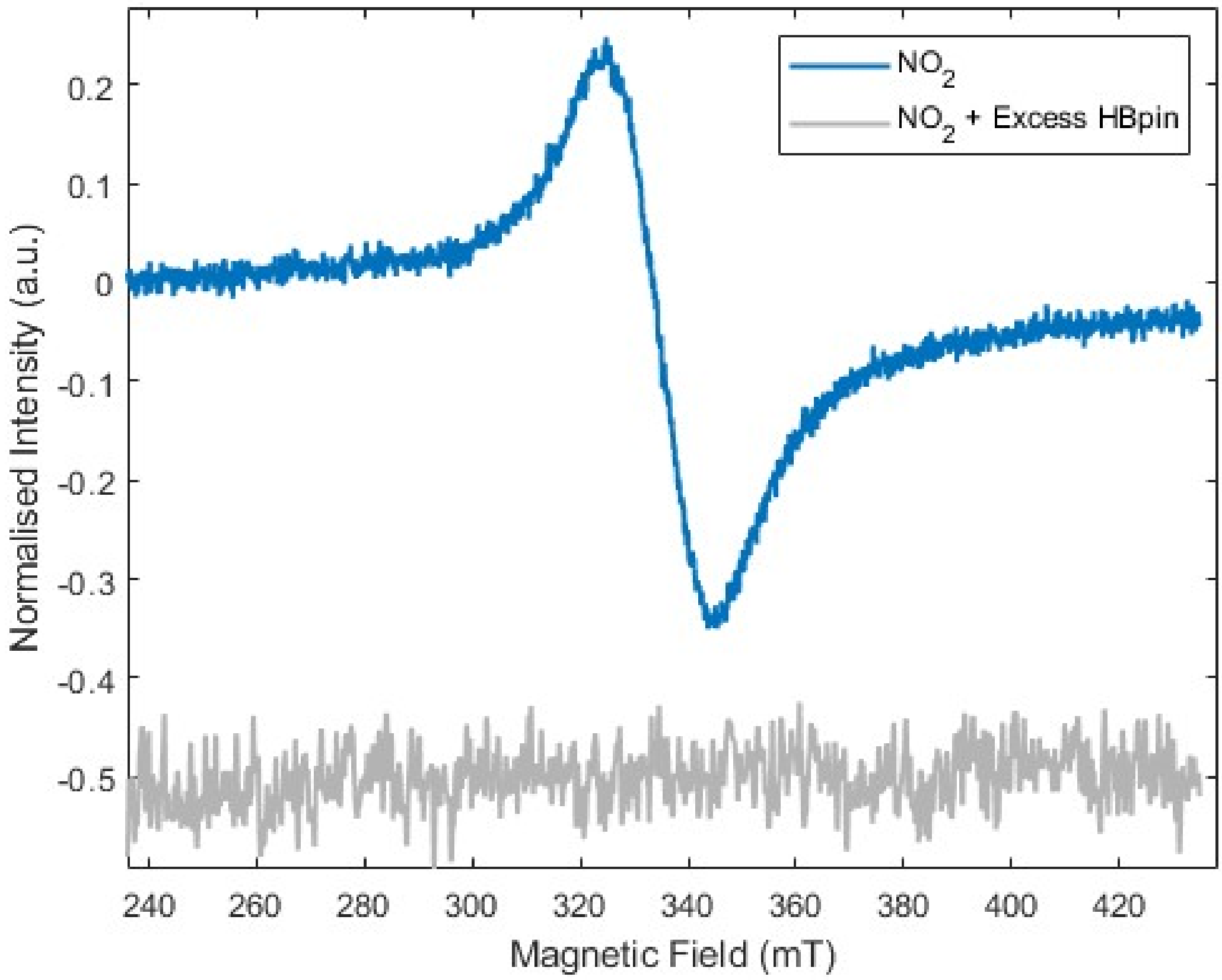

Figure S41. Continuous wave EPR spectra of NO_2 before and after being treated with an excess of HBpin, indicating the complete consumption of NO_2. Experimental parameters: solvent: toluene, frequency: 9.935004 GHz, temperature: 298 K, modulation amplitude 4.00 G, 1 scan, receiver gain 30 dB

8.2 Attempted synthesis of pinBOBpin from dehydrocoupling with HBpin with HOBpin in C_6D_6

The HBpin was purified by stirring it over triphenylphosphine overnight followed by distillation. In an N_2 filled glovebox, HBpin (13.2 mg, 0.0735 mmol 1 equiv) was dissolved in C_6D_6 (0.6 mL) and added to a J. Young NMR tube. The NMR tube was cycled onto a Schlenk apparatus, and H_2O (0.8 equiv) was added under a positive pressure of argon gas. Vigorous gas evolution was observed. The reaction mixture was analysed by ^1H and $^{11}B\{^1H\}$ NMR spectroscopy which confirmed the formation of HOBpin and H_2. A second equivalent of HBpin was added to the reaction mixture. No gas evolution was observed and the reaction mixture was left to react for 12 hours. Analysis of the reaction mixture by ^1H and $^{11}B\{^1H\}$ NMR spectra showed no formation of pinBOBpin.

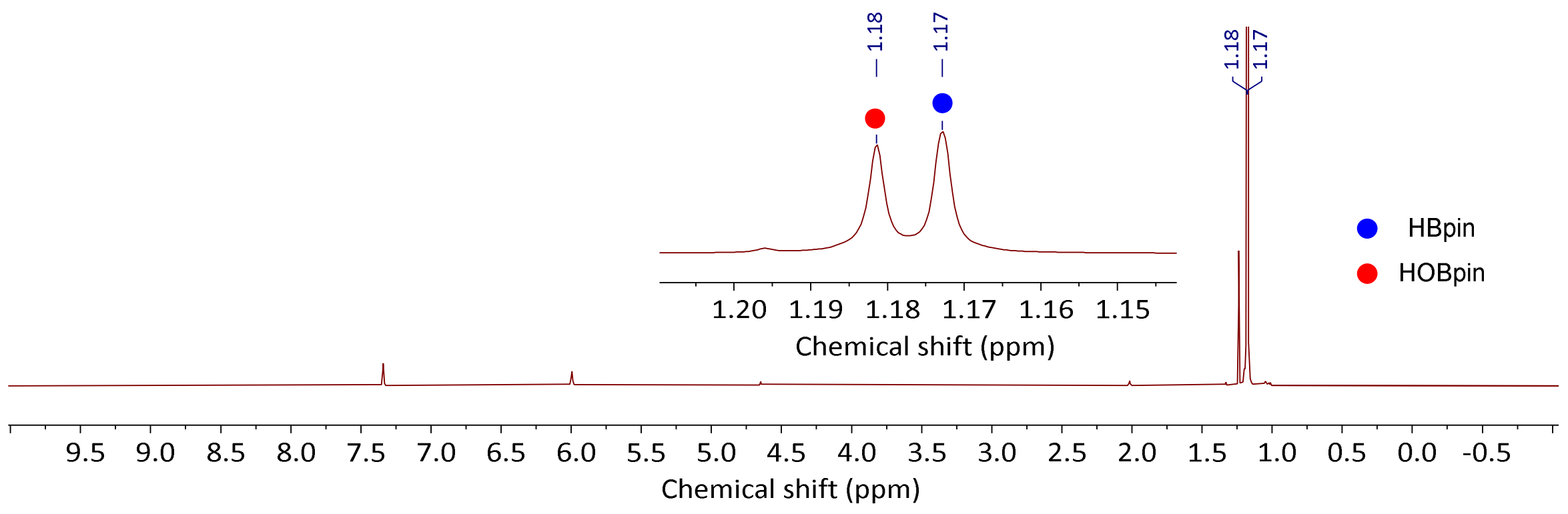

Figure S42. ^{1}H NMR spectrum (400 MHz, C_6D_6) of HBpin and HOBpin after 12 hours at room temperature in C_6D_6. No formation of pinBOBpin was observed.

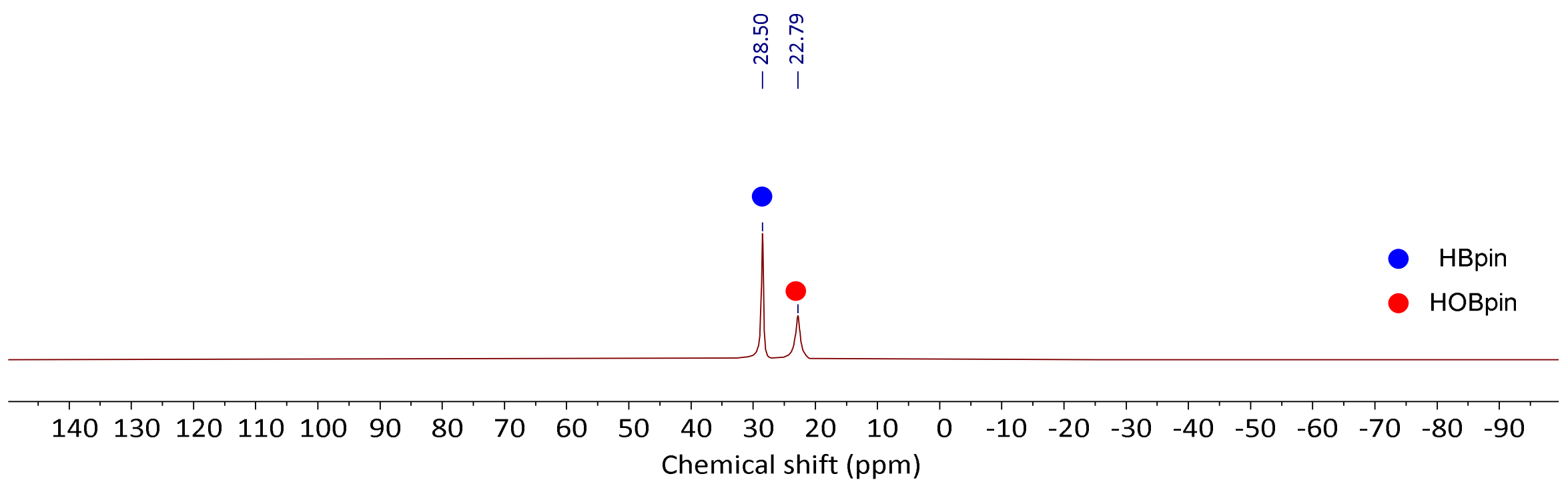

Figure S43. $^{11}B\{^1H\}$ NMR spectrum (128 MHz, C_6D_6) of HBpin and HOBpin after 12 hours at room temperature in C_6D_6. No formation of pinBOBpin was observed.

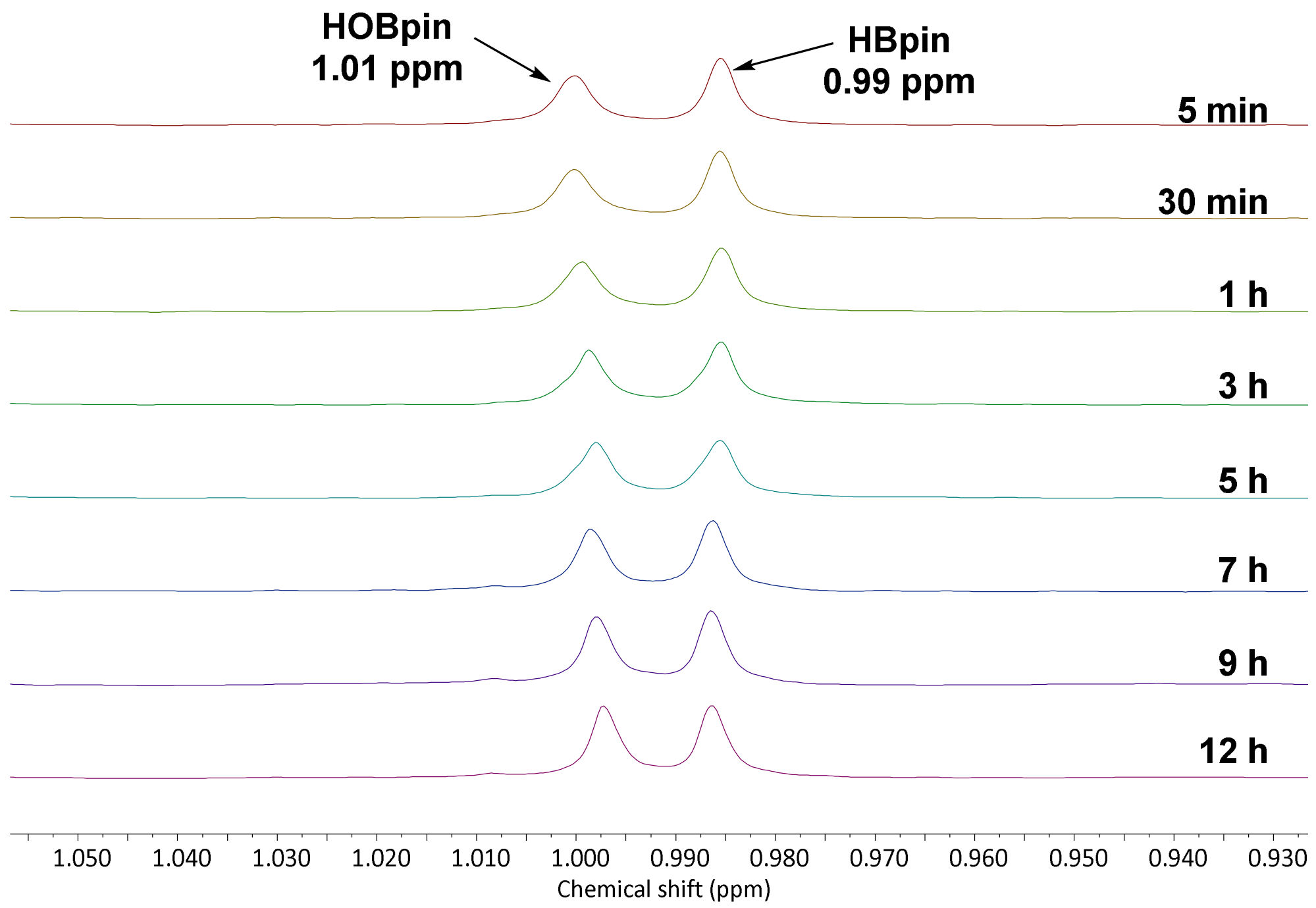

Figure S44. Time-dependent ^{1}H NMR spectrum (400 MHz, C_6D_6) of HBpin and HOBpin after 12 hours at room temperature in C_6D_6. No formation of pinBOBpin was observed.

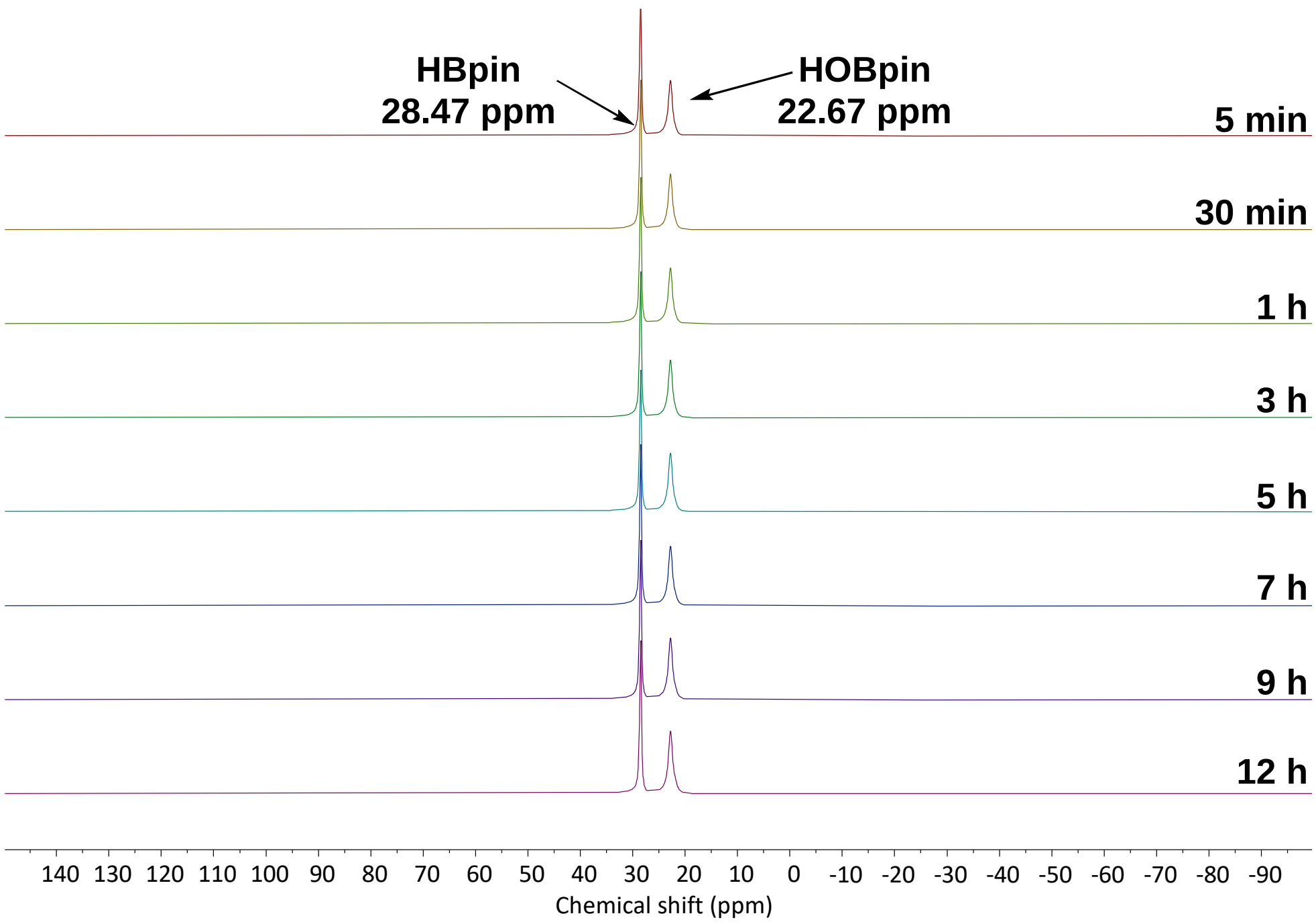

Figure S45. Time-dependent ^{11}B{^{1}H} NMR spectrum (128 MHz, C_6D_6) of HBpin and HOBpin after 12 hours at room temperature in C_6D_6. No formation of pinBOBpin was observed.

8.3 Reaction of NO_2 with Excess 1,4-cyclohexadiene

In an N_2 filled glovebox, 1,4-cyclohexadiene (34.8 μL, 0.3675 mmol) was dissolved in C_6D_6 (0.7 mL) and added to a J. Young EPR tube. The solution was degassed and pressurised with NO_2 (~1 atm, 25 °C). The J. Young EPR tube was inverted several times, resulting in the immediate formation of a deep green solution, which gradually turned increasingly orange. The reaction mixture was analysed by EPR spectroscopy, which indicated the complete consumption of NO_2. The reaction mixture was analysed daily by EPR spectroscopy over the course of a week. No decomposition back into NO_2 was observed.

9 Density Functional Theory

9.1 Discounted radical HBpin mechanisms

Given the radical nature of NO_2, H atom abstraction by NO_2 from HBpin was also considered, as shown in Figure S44. This was found to be 38 kcal mol^{-1} uphill. Additionally, the homolytic cleavage of HBpin was calculated to be 95 kcal mol^{-1} uphill. Finally, the lack of B_2pin_2 evolution in the reaction mixture experimentally suggests that $^{\bullet}$Bpin is not being generated. This reaction pathway was discounted, and a full mechanistic profile was not pursued.

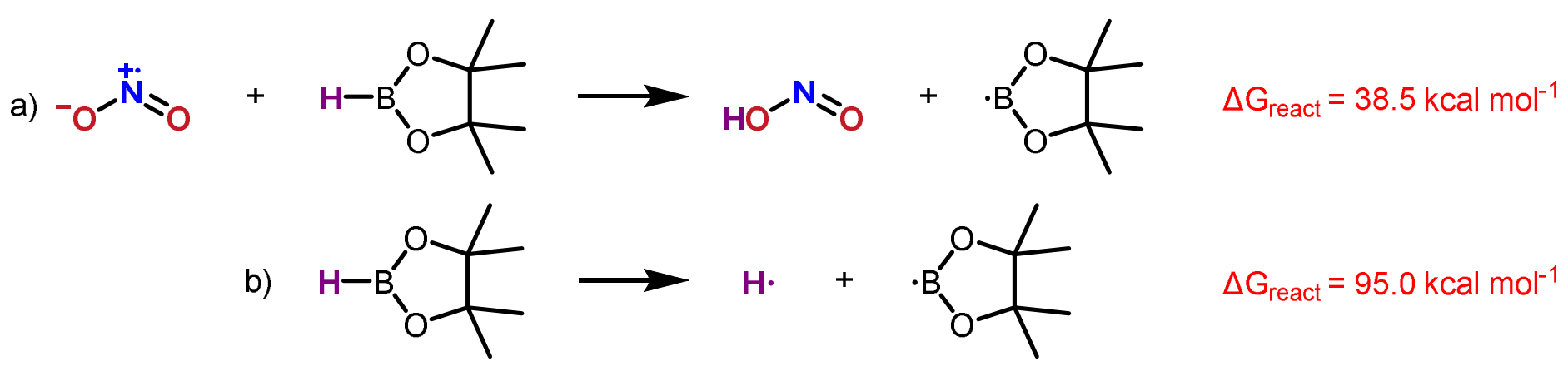

Figure S46. a) The highly endergonic H atom abstraction and b) homolytic fission of the H–B bond in HBpin. Gibbs energies in kcal mol^{-1} (r^{2}SCAN-3c composite method with CPCM benzene solvation).

9.2 Discounted three-membered HBpin pathway

A three-membered pathway for HOBpin and NO formation was considered, as shown in Figure S45 **TS-a**, and was found to be 4.5 kcal mol^{-1} higher in energy than the four-membered pathway **TS-2**, shown in Figure 4 of the manuscript.

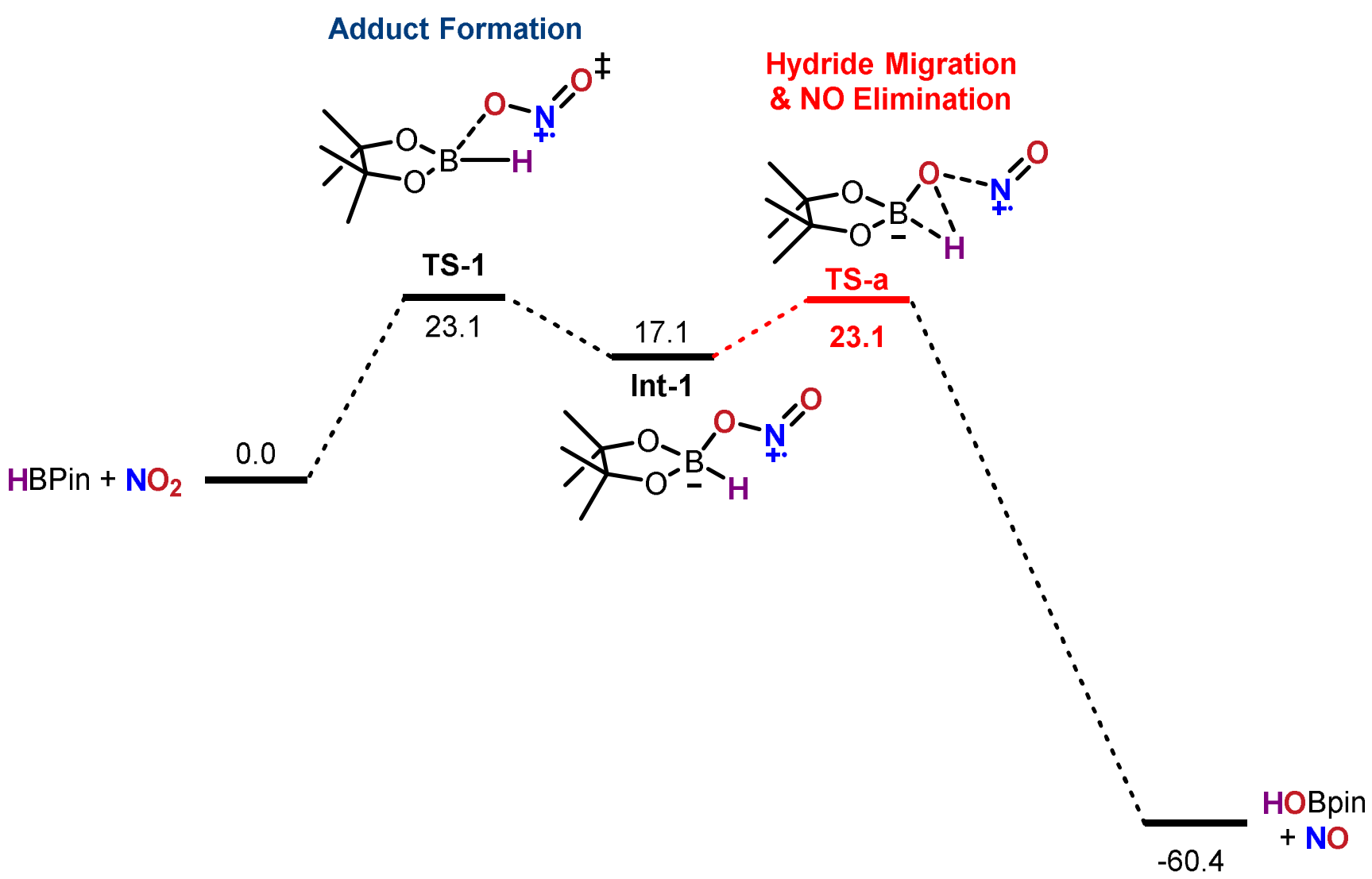

Figure S47. The discounted three-membered transition state **TS-a** highlighted in red. Gibbs energies in kcal mol^{-1} (r^{2}SCAN-3c composite method with CPCM benzene solvation).

9.3 Discounted five-membered HBpin pathway

A five-membered pathway for HOBpin and NO formation was also considered. Despite being geometrically accessible, this pathway was discounted due to the higher energy associated with a five-membered transition state. **TS-b** was again found to be 4.5 kcal mol^{-1} more energetically unfavourable than the analogous four-membered transition state **TS-2**, shown in Figure 4 of the manuscript. Additionally, the thermodynamic stability of **Int-b** resulted in a significantly higher barrier for NO elimination (**TS-c** 42.3 kcal mol^{-1} vs **TS-3** 17 kcal mol^{-1}; **TS-3** shown in Figure 4 of the manuscript).

Additional computations sought to ascertain whether **Int-b** could act as a vector for dehydrocoupling with another equivalent of HBpin. Again, the stability of **Int-b** resulted in higher barriers for dehydrocoupling (**TS-c'** 46 kcal mol^{-1} vs TS-**4** 32 kcal mol^{-1}, **TS-4** shown in Figure 4 of the manuscript).

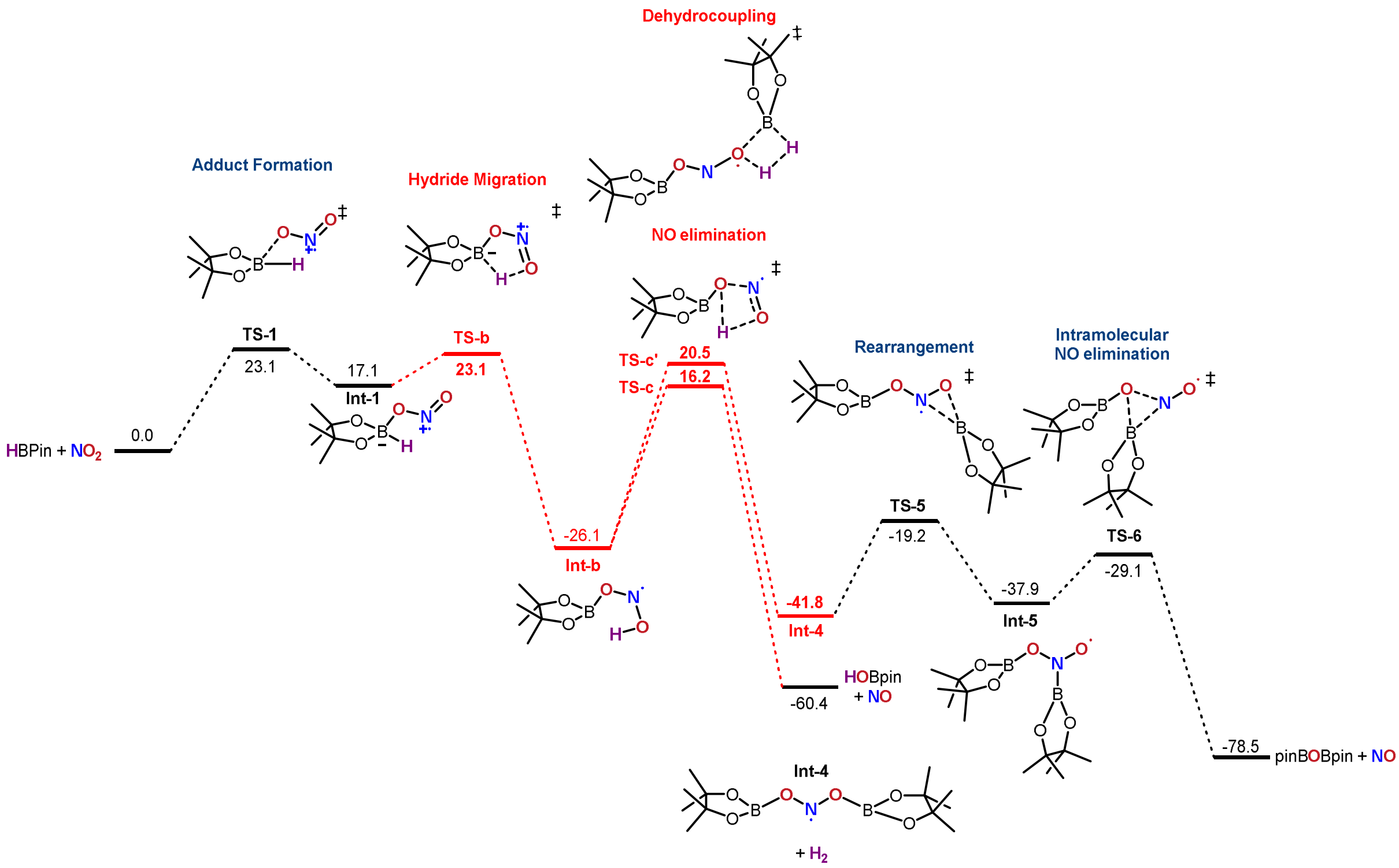

Figure S48. The discounted five-membered transition state. The less energetically feasible steps, compared to a four-membered pathway, have been highlighted in red. Gibbs energies in kcal mol^{-1} (r^2SCAN-3c composite method with CPCM benzene solvation).

9.4 Discounted dehydrocoupling mechanism between HOBpin and HBpin

The formation of pinBOBpin was experimentally and computationally explored. Computational studies are in agreement with experiments carried out in Section 8.2. The direct uncatalysed dehydrocoupling reaction between HBpin and HOBpin was found to be energetically infeasible.

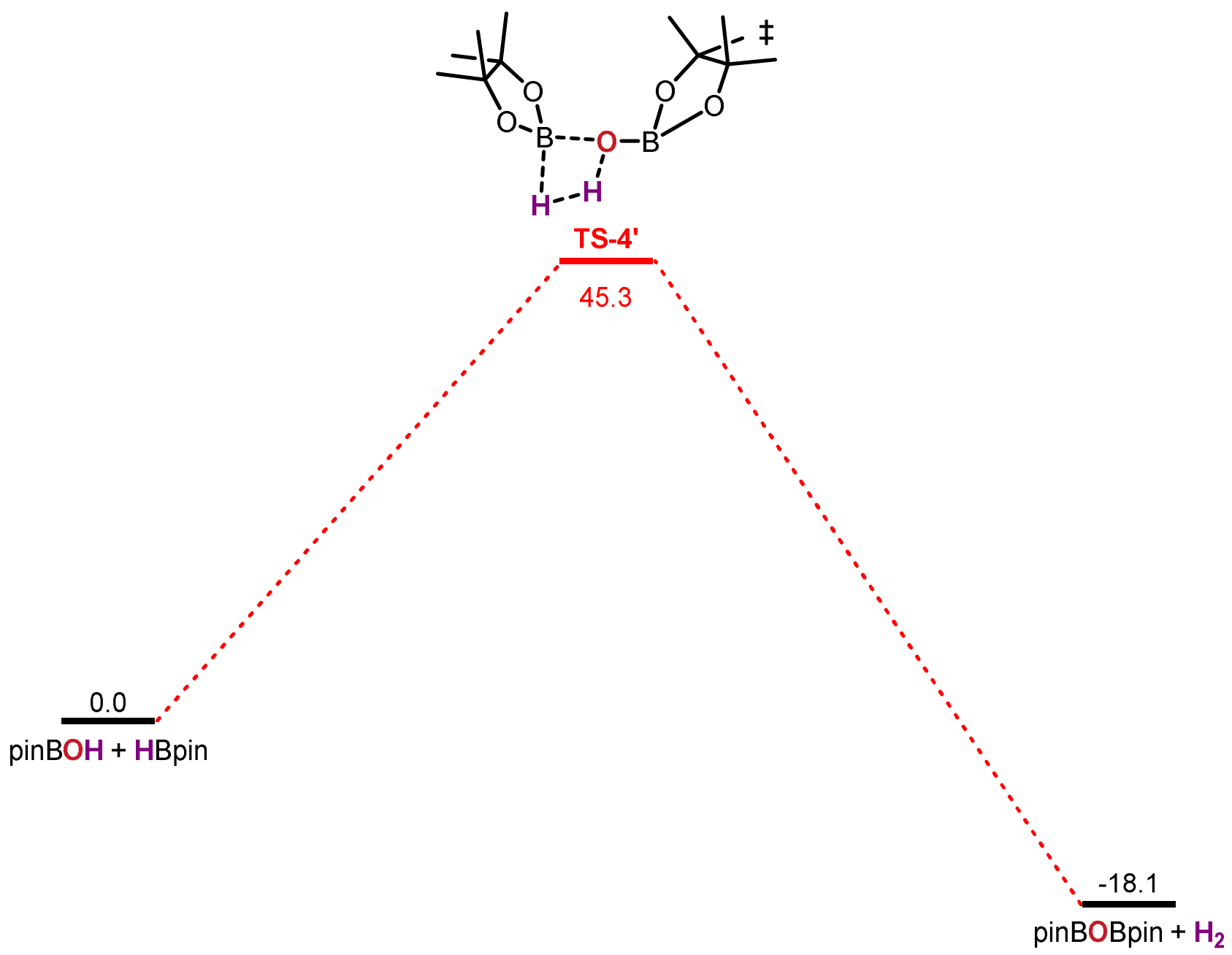

Figure S49. The discounted mechanism for the direct dehydrocoupling of HBpin and HOBpin. Gibbs energies in kcal mol^{-1} (r^{2}SCAN-3c composite method with CPCM benzene solvation).

9.5 Mulliken Atomic Charges and Spin Populations for HBpin Reduction Intermediates

Table 1: Mulliken Atomic Charges and Spin Populations for **Int-1**

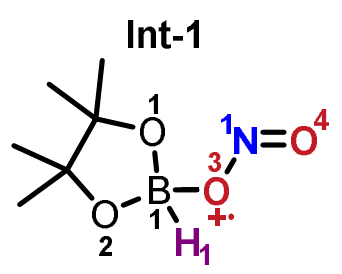

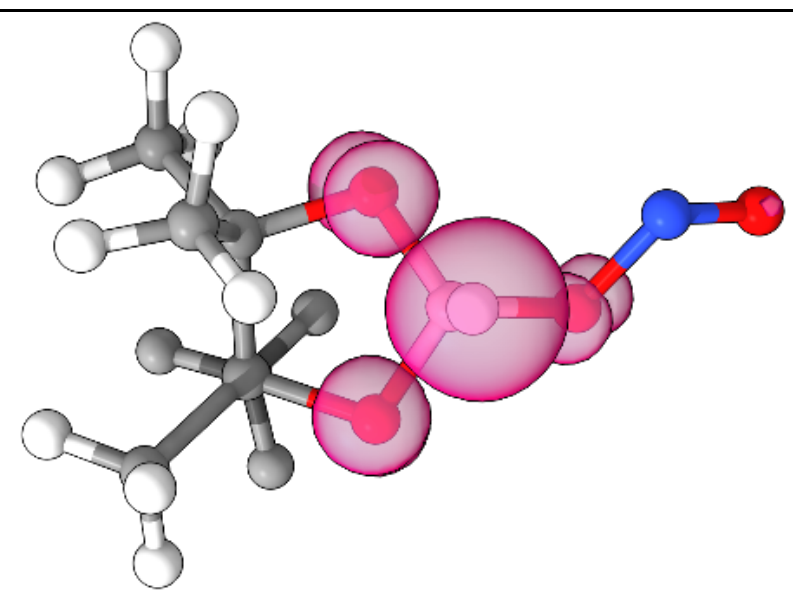

Atom	Atomic Charges	Spin Population
C	0.215673	-0.025245
C	0.169898	-0.009834
2 B	0.156725	0.064354
1 O	-0.268929	0.189637
2 O	-0.266143	0.201809
C	-0.531011	0.017284
H	0.177555	-0.000476
H	0.176163	0.010082
H	0.179046	-0.000634
C	-0.512868	0.014550
H	0.183447	0.001984
H	0.167959	-0.000888
H	0.174284	-0.000602
C	-0.508276	0.016126
H	0.182786	-0.000175
H	0.173904	-0.001794
H	0.170702	-0.001733
C	-0.504777	0.000862
H	0.181417	-0.000732
H	0.168112	-0.000363
H	0.169849	0.000314
3 O	-0.080854	0.061232
1 N	-0.148705	-0.013996
1 H	0.164919	0.473070
4 O	0.009125	0.005168

Table 2: Mulliken Atomic Charges and Spin Populations for **Int-2**

Int-2 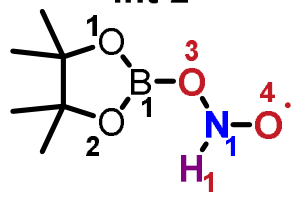	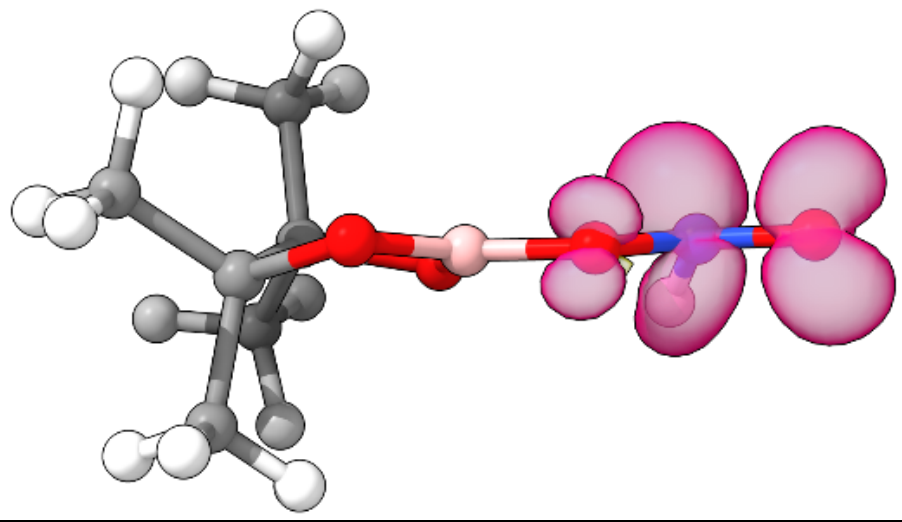	
**Atom**	**Atomic Charges**	**Spin Population**
C	0.179812	-0.000483
C	0.191156	-0.000913
**1 B**	**0.725536**	**0.002852**
**1 O**	**-0.423086**	**0.003558**
**2 O**	**-0.405649**	**-0.002775**
C	-0.509232	0.000659
H	0.175387	-0.000032
H	0.173389	-0.000007
H	0.172036	-0.000061
C	-0.515767	0.000217
H	0.181083	0.000017
H	0.166851	-0.000016
H	0.171277	-0.000013
C	-0.509272	0.000306
H	0.177416	-0.000018
H	0.172719	-0.000063
H	0.167880	-0.000034
C	-0.515453	-0.000007
H	0.182681	-0.000017
H	0.166063	-0.000004
H	0.170252	-0.000004
**3 O**	**-0.183042**	**0.040174**
**1 N**	**-0.276728**	**0.420512**
**1 H**	**0.303707**	**0.023606**
**4 O**	**-0.139016**	**0.512546**

Table 3: Mulliken Atomic Charges and Spin Populations for **Int-4**

Int-4 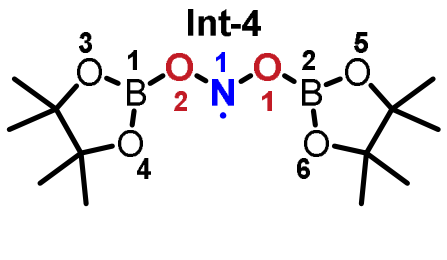	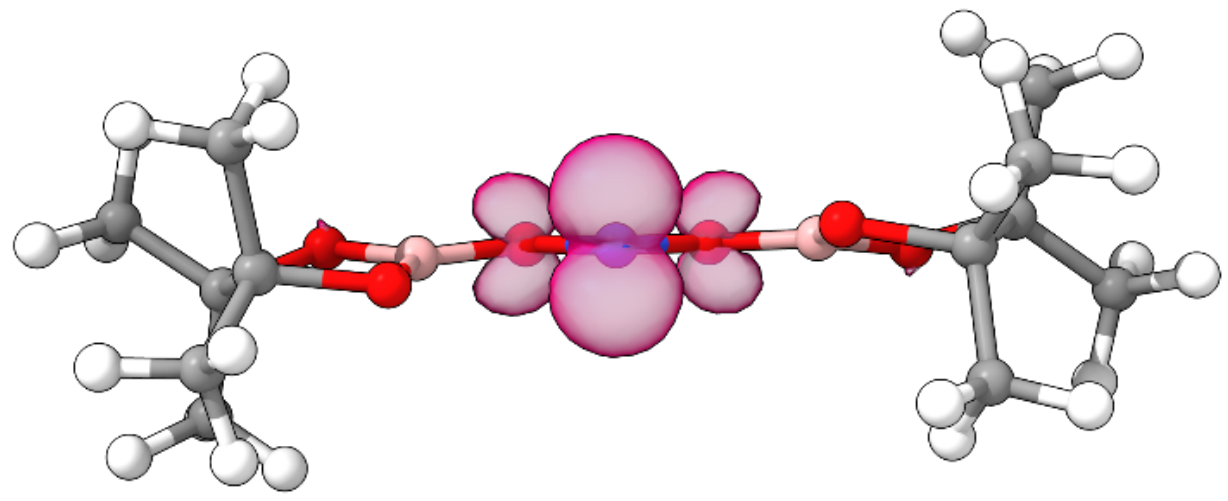	
**Atom**	**Atomic Charges**	**Spin Population**
**1 N**	**-0.499198**	**0.842396**
**1 O**	**-0.062994**	**0.082433**
**2 O**	**-0.062284**	**0.083000**

**1 B**	**0.715582**	**-0.006663**
**3 O**	**-0.409800**	**0.006679**
**4 O**	**-0.384187**	**-0.003566**
C	0.193926	-0.000844
C	0.187570	-0.000428
C	-0.518197	0.000187
C	-0.512291	0.000397
C	-0.510682	0.000241
C	-0.516339	-0.000006
H	0.165856	-0.000023
H	0.180319	-0.000060
H	0.169886	-0.000009
H	0.171635	0.000103
H	0.176075	-0.000011
H	0.169691	-0.000050
H	0.171315	0.000033
H	0.176588	0.000004
H	0.169312	-0.000032
H	0.165950	-0.000009
H	0.180243	-0.000026
H	0.169548	-0.000002
**2 B**	**0.716935**	**-0.006256**
**5 O**	**-0.384292**	**-0.003570**
**6 O**	**-0.410191**	**0.006700**
C	0.185783	-0.000562
C	0.194061	-0.000783
C	-0.516132	0.000002
C	-0.509705	0.000217
C	-0.512045	0.000382
C	-0.518169	0.000221
H	0.165946	-0.000010
H	0.180093	-0.000025
H	0.169644	-0.000003
H	0.171184	0.000026
H	0.176550	0.000004
H	0.169400	-0.000028
H	0.171521	0.000091
H	0.176164	-0.000010
H	0.169437	-0.000049
H	0.165693	-0.000023
H	0.180545	-0.000057
H	0.170056	-0.000011

Table 4: Mulliken Atomic Charges and Spin Populations for **Int-5**

Int-5 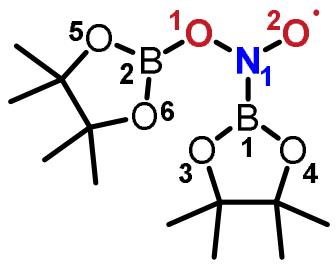	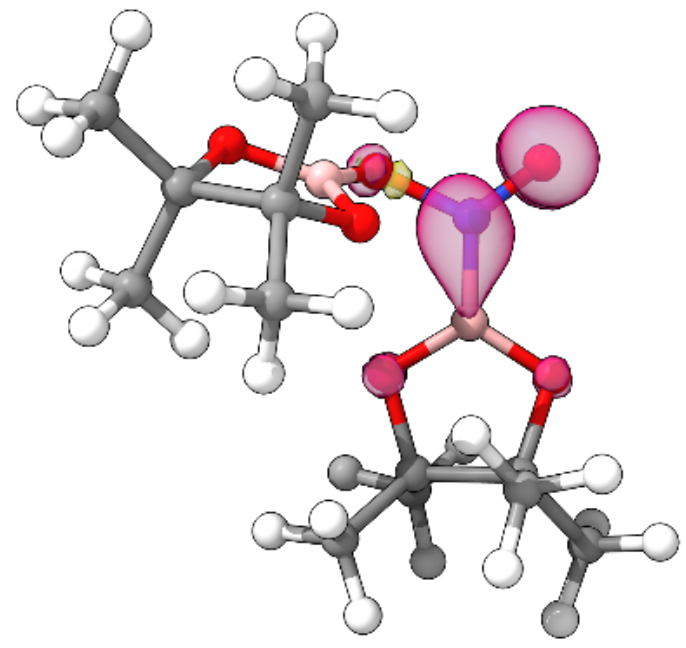	
**Atom**	**Atomic Charges**	**Spin Population**
**1 N**	**-0.150147**	**0.470588**
**1 O**	**-0.193983**	**-0.025428**
**2 O**	**-0.208456**	**0.538466**
**1 B**	**0.648334**	**-0.017711**
**3 O**	**-0.362410**	**0.010758**
**4 O**	**-0.410496**	**0.021013**
C	0.176197	-0.004178
C	0.199982	-0.000647
C	-0.518678	0.000735
C	-0.506058	0.000983
C	-0.519970	0.001007
C	-0.505718	0.000751
H	0.169988	-0.000070
H	0.182343	-0.000037
H	0.170443	-0.000022
H	0.174113	0.000369
H	0.178011	-0.000004
H	0.172264	-0.000104
H	0.174384	0.000255
H	0.177708	0.000014
H	0.174611	-0.000147
H	0.168033	-0.000062
H	0.181420	-0.000072
H	0.171962	-0.000018
**2 B**	**0.660034**	**0.008085**
**5 O**	**-0.370125**	**-0.005113**
**6 O**	**-0.416820**	**0.000472**
C	0.179265	-0.000443
C	0.207345	0.000400
C	-0.527117	-0.000092
C	-0.508340	0.000006
C	-0.526048	0.000135
C	-0.516150	0.000091
H	0.168236	0.000018
H	0.180781	-0.000026
H	0.168121	0.000019
H	0.171527	0.000056
H	0.175236	0.000016

H	0.173592	-0.000020
H	0.171969	0.000016
H	0.175221	-0.000006
H	0.173181	-0.000023
H	0.165410	0.000019
H	0.179525	-0.000046
H	0.171278	-0.000004

Table 5: Mulliken Atomic Charges and Spin Populations for **Int-b**

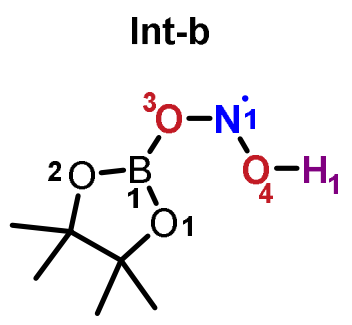	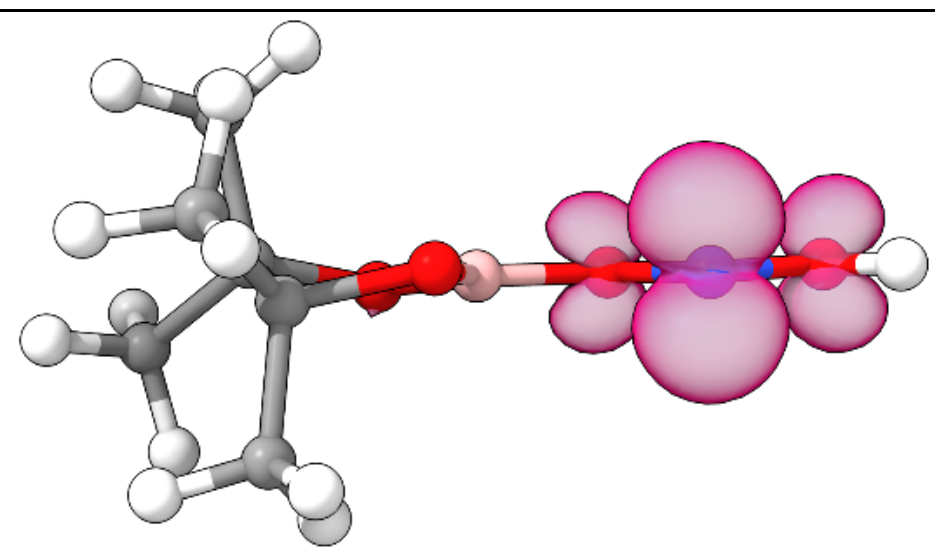	
**Atom**	**Atomic Charges**	**Spin Population**
C	0.194241	-0.000893
C	0.190264	-0.000426
**1 B**	**0.709907**	**-0.002671**
**1 O**	**-0.389380**	**-0.004562**
**2 O**	**-0.413037**	**0.005817**
C	-0.512274	0.000362
H	0.176055	-0.000014
H	0.171892	0.000075
H	0.170080	-0.000050
C	-0.515795	-0.000018
H	0.180922	-0.000023
H	0.165824	-0.000009
H	0.170362	-0.000002
C	-0.511100	0.000281
H	0.176195	0.000004
H	0.171819	0.000019
H	0.170177	-0.000032
C	-0.517905	0.000163
H	0.180799	-0.000055
H	0.165872	-0.000023
H	0.170509	-0.000009
**3 O**	**-0.142659**	**0.066210**
**1 N**	**-0.361525**	**0.802718**
**4 O**	**-0.149638**	**0.140549**
**1 H**	**0.348394**	**-0.007412**

9.6 Discounted proton-hydride mechanisms for transfer hydrogenation

1,4-Cyclohexadiene is known to be able to react in both a radical and stepwise two-electron fashion.[16] Stepwise proton-hydride mechanisms were considered and

subsequently discounted. The transfer of a hydride or a proton from 1,4-cyclohexadiene to NO_2 was found to be 158 kcal mol^{-1} and 272 kcal mol^{-1} uphill, respectively. A full mechanistic profile was not considered.

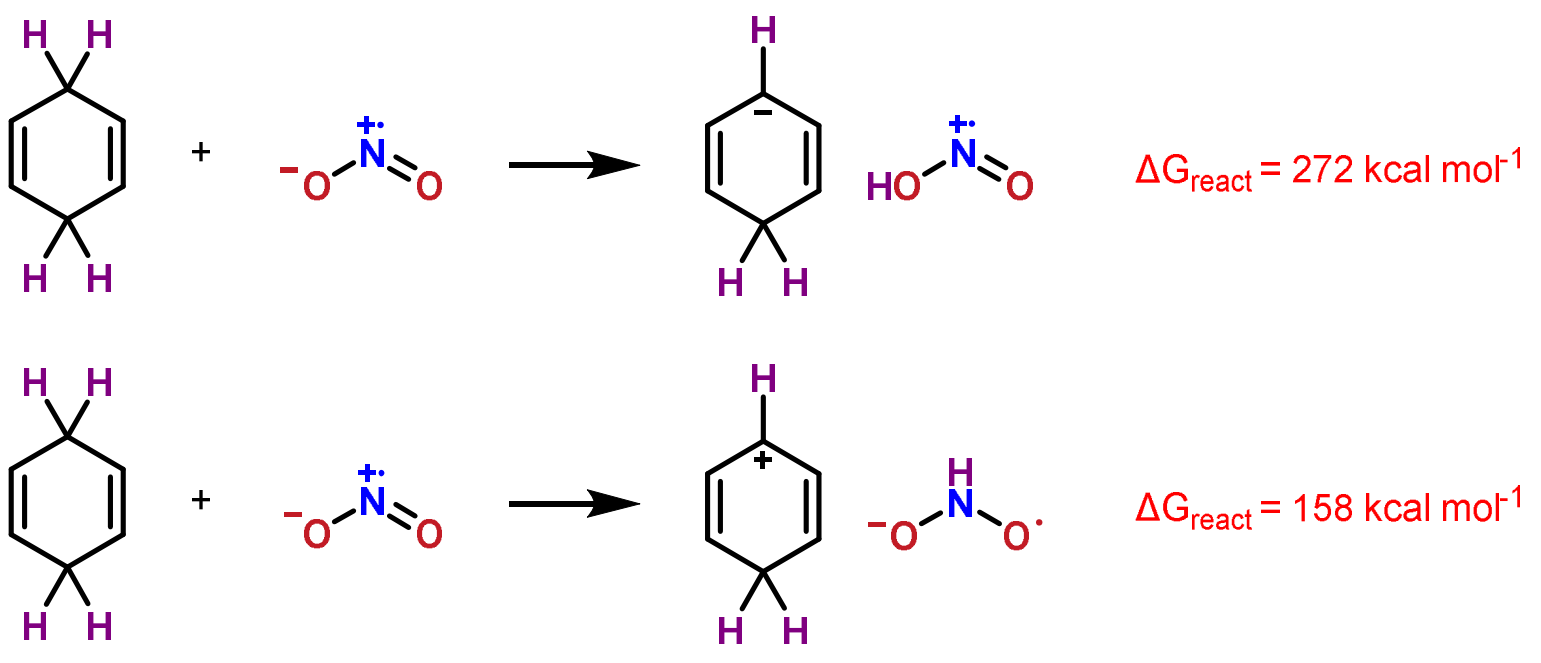

Figure S50. The calculated energy differences for hydride and proton abstraction from 1,4-cyclohexadiene by NO_2. Gibbs energies in kcal mol^{-1} (r^2SCAN-3c composite method with CPCM benzene solvation).

9.7 Discounted intramolecular isomerisation of O(H)NO to HONO

O(H)NO was found to be 5.7 kcal mol^{-1} more stable compared to HONO, the canonical isomer of nitrous acid. Intramolecular isomerisation of O(H)NO to HONO was found to be energetically inaccessible. An alternative six-membered intermolecular transition state, **TS-8** shown in Figure 5 of the manuscript, was found to be energetically accessible (**TS-d** 47.2 kcal mol^{-1} vs **TS-8** 7.9 kcal mol^{-1}, **TS-8** shown in Figure 5 of the manuscript).

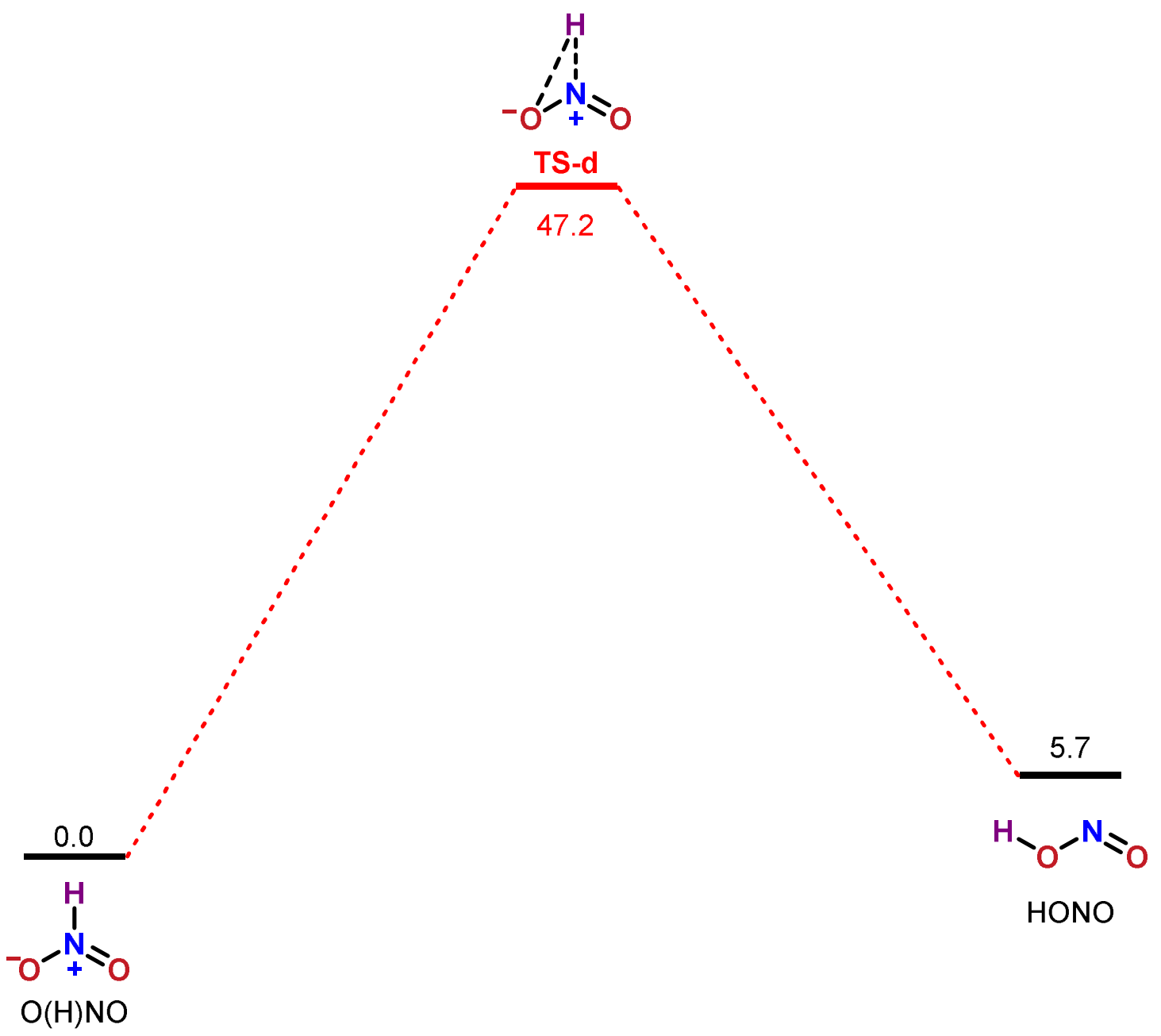

Figure S51. The discounted intramolecular isomerisation of O(H)NO to HONO. Gibbs energies in kcal mol^{-1} (r^2SCAN-3c composite method with CPCM benzene solvation).

9.8 DFT Coordinates

**HBpin**			
Single-point energy (Eh)		-411.7754107	
Gibbs free energy (Eh)		-411.6190664	
**Atom**	**X**	**Y**	**Z**
C	-4.77360100	3.79032100	-0.11041600
C	-6.03704100	2.86406700	-0.16969700
B	-4.15046500	1.60593900	0.01116700
H	-3.45124700	0.64697500	0.09482600
O	-3.73185500	2.86592600	0.34268400
O	-5.44605900	1.54866600	-0.42528500
C	-4.87006000	4.94213800	0.87427600
H	-3.94406000	5.52399700	0.84929300
H	-5.69800000	5.60613200	0.60284000
H	-5.02319500	4.58856300	1.89568500
C	-6.76631100	2.75726800	1.16702200
H	-7.29600100	3.68399900	1.40858800
H	-7.49750400	1.94621500	1.10562400
H	-6.06948400	2.52764300	1.97889600
C	-7.01559600	3.18582600	-1.28548900
H	-7.85391000	2.48405700	-1.25028300
H	-7.41370400	4.19917300	-1.16388300
H	-6.54630200	3.10801500	-2.26799100
C	-4.33489000	4.29572200	-1.48242000
H	-5.02623500	5.05029800	-1.87061500
H	-3.34371800	4.74881800	-1.39070700
H	-4.26911100	3.47448700	-2.20247100

pinBOBpin			
Single-point energy (Eh)		-897.7009668	
Gibbs free energy (Eh)		-897.379995	
**Atom**	**X**	**Y**	**Z**
C	-4.75738000	3.42063500	-0.75518900
C	-5.24552100	3.46529100	0.73730900
B	-3.97217300	1.61935000	0.38142100
O	-3.69739400	2.41158000	-0.70465000
O	-4.97673900	2.09809800	1.18844000
C	-4.17213500	4.72322400	-1.26888500
H	-3.86689600	4.60271800	-2.31270500
H	-4.92447400	5.51781800	-1.22213600
H	-3.30033300	5.02939200	-0.68694500
C	-4.40453300	4.38823500	1.61387600
H	-4.59534600	5.43970800	1.37828600
H	-4.66663300	4.21391300	2.66153400
H	-3.33539100	4.18809500	1.49032400
C	-6.72382000	3.76020300	0.91242900

H	-6.97178600	3.77768900	1.97806000
H	-6.96381900	4.74133300	0.48892400
H	-7.34479500	3.00554000	0.42545400
C	-5.82015000	2.89306000	-1.71427900
H	-6.62309600	3.62296100	-1.85591400
H	-5.35374800	2.69862300	-2.68453000
H	-6.25570500	1.95799700	-1.34778000
O	-3.31928800	0.43711600	0.58939100
B	-3.06428700	-0.17575900	1.78354300
O	-2.92309500	-1.53726200	1.87886700
O	-2.87888200	0.49379900	2.96971100
C	-2.38729200	-1.80252500	3.21581300
C	-2.84461600	-0.53063300	4.01594400
C	-2.96296500	-3.11128600	3.72505800
C	-0.87216600	-1.90310400	3.07040400
C	-4.26525400	-0.64564700	4.56002400
C	-1.88752800	-0.08533600	5.10614500
H	-2.59156100	-3.93870800	3.11292500
H	-2.64757500	-3.28289300	4.75975300
H	-4.05416300	-3.11384500	3.68425000
H	-0.40084800	-2.16616500	4.02233500
H	-0.63940800	-2.68476300	2.34129200
H	-0.44340200	-0.96097200	2.71423600
H	-4.31498800	-1.35180100	5.39457800
H	-4.58517100	0.33720800	4.91819600
H	-4.96325800	-0.97087200	3.78191000
H	-2.28828700	0.80309000	5.60352500
H	-1.77421100	-0.87518700	5.85641200
H	-0.90327900	0.15959300	4.70167500

**HOBpin**			
Single-point energy (Eh)		-487.0638123	
Gibbs free energy (Eh)		-486.9028475	
**Atom**	**X**	**Y**	**Z**
C	-4.74725100	3.80809900	-0.07797300
C	-5.98309000	2.84031200	-0.12104300
B	-4.05858900	1.65548100	0.13799500
O	-3.69375300	2.94288900	0.45322500
O	-5.34572200	1.53956900	-0.34377900
C	-4.90981100	5.00362900	0.84291400
H	-4.00735500	5.62134900	0.80440900
H	-5.75819500	5.61749800	0.52165600
H	-5.07098700	4.69430800	1.87771800
C	-6.71529400	2.75067200	1.21449400
H	-7.26921500	3.67075500	1.42425200
H	-7.42760300	1.92126300	1.17234500
H	-6.01732100	2.56569900	2.03734100
C	-6.96200900	3.10678400	-1.24992800

H	-7.78472300	2.38716700	-1.19839600
H	-7.38285500	4.11354000	-1.15528600
H	-6.48404800	3.01560300	-2.22743800
C	-4.29469100	4.25528700	-1.46514400
H	-5.00426800	4.96251600	-1.90559100
H	-3.32298400	4.74986100	-1.37658100
H	-4.18572200	3.40034900	-2.14010600
O	-3.19435700	0.61443600	0.29793600
H	-3.59870200	-0.23627200	0.09356000

**Bpin Radical**			
Single-point energy (Eh)		-411.0993302	
Gibbs free energy (Eh)		-410.953976	
**Atom**	**X**	**Y**	**Z**
C	-4.78638500	3.80782784	-0.10610676
C	-6.05009443	2.88199932	-0.16223476
B	-4.16988022	1.63059751	0.03213990
O	-3.73738301	2.87460576	0.36615868
O	-5.45061036	1.55139941	-0.41447253
C	-4.88026855	4.96093676	0.87476782
H	-3.95070422	5.53700857	0.85254871
H	-5.70270994	5.62927571	0.59621826
H	-5.04156664	4.61159962	1.89626015
C	-6.78146946	2.77617607	1.17109561
H	-7.31541103	3.70263115	1.40558470
H	-7.50988946	1.96276471	1.11034794
H	-6.08723722	2.55275063	1.98656728
C	-7.02145433	3.19082340	-1.28578895
H	-7.85696543	2.48585274	-1.25092161
H	-7.42478493	4.20334481	-1.17210067
H	-6.54603377	3.10969565	-2.26494148

**H radical**			
**Atom**	**X**	**Y**	**Z**
H	-3.27425421	0.39648383	0.00000000

**H_2**			
Single-point energy (Eh)		-1.16954968	
Gibbs free energy (Eh)		-1.17079419	
**Atom**	**X**	**Y**	**Z**
H	3.89118700	1.04292100	0.01365300
H	3.14967300	1.04292100	0.01365300

**CHD**	
Single-point energy (Eh)	-233.3494207
Gibbs free energy (Eh)	-233.2566647

Atom	X	Y	Z
C	-2.40851700	0.33454600	-0.03179100
C	-1.07769300	0.33447300	-0.03152200
C	-0.24844300	1.58422900	-0.03176600
C	-1.07755700	2.83408000	-0.03310900
C	-2.40837900	2.83415300	-0.03337300
C	-3.23762800	1.58439200	-0.03271800
H	-2.94460800	-0.61301000	-0.03155000
H	-0.54170600	-0.61314800	-0.03087600
H	0.42686300	1.58475400	0.83937200
H	-0.54144700	3.78162900	-0.03367400
H	-2.94438100	3.78176200	-0.03434400
H	-3.91339000	1.58388700	-0.90349500
H	0.42797000	1.58365300	-0.90205000
H	-3.91359000	1.58498400	0.83792200

**CHD cation**			
Single-point energy (Eh)		-232.4817917	
Gibbs free energy (Eh)		-232.4000496	
**Atom**	**X**	**Y**	**Z**
C	-2.40072788194517	0.33409336605489	-0.02989309837585
C	-1.03338037005539	0.34869671158471	-0.03153368551785
C	-0.36059015374930	1.58426037098395	-0.03310389972937
C	-1.03326215268816	2.81988919034648	-0.03312491988123
C	-2.40060870044211	2.83462496576923	-0.03149900565360
C	-3.15896441952379	1.58439518733441	-0.03126740806350
H	-2.95186550395181	-0.60314118395125	-0.02859479559706
H	-0.46413198520690	-0.57519031025329	-0.03128255970074
H	-0.46393180820008	3.74372520443172	-0.03407078079394
H	-2.95165420822615	3.77191450043253	-0.03140940477540
H	-3.87390198236265	1.58388727096414	-0.88150388881384
H	0.72750610717746	1.58420683521652	-0.03441628113481
H	-3.88378055082594	1.58496663108595	0.80985048803720

**CHD anion**			
Single-point energy (Eh)		-232.7310448	
Gibbs free energy (Eh)		-232.6536355	
**Atom**	**X**	**Y**	**Z**
C	-2.39372820191895	0.33537918703902	-0.03355222320371
C	-1.02501524981968	0.38389823447013	-0.03124593043579
C	-0.27342961171427	1.58425937181945	-0.02907854030523
C	-1.02490102621039	2.78468828009305	-0.03278878681437
C	-2.39360926458869	2.83333463506310	-0.03515681132159
C	-3.26252344296176	1.58440009223854	-0.03145533003281
H	-2.89652916637539	-0.63228511426182	-0.03585882670410
H	-0.48817472087730	-0.56920130028087	-0.03189300540575
H	-0.48796983999890	3.73773512442513	-0.03466733639557

H	-2.89631797775063	3.80104302914366	-0.03871685042873
H	-3.96166884024391	1.58387560124064	-0.90221191463411
H	0.81129315402140	1.58420806287180	-0.02875123316266
H	-3.95671942156149	1.58499353613813	0.84352754884443

**CHD radical**			
Single-point energy (Eh)		-232.7313955	
Gibbs free energy (Eh)		-232.652078	
**Atom**	**X**	**Y**	**Z**
C	-2.39658300	0.33216500	-0.03237100
C	-1.03363300	0.36223000	-0.03148200
C	-0.32093800	1.58426000	-0.03152400
C	-1.03351600	2.80635700	-0.03305100
C	-2.39646400	2.83655100	-0.03397900
C	-3.21673500	1.58439700	-0.03266000
H	-2.92389700	-0.61802800	-0.03239200
H	-0.47880400	-0.57281800	-0.03088200
H	-0.47859900	3.74135300	-0.03366200
H	-2.92368700	3.78679300	-0.03523300
H	-3.90580400	1.58387600	-0.89866100
H	0.76347300	1.58420900	-0.03074600
H	-3.90410700	1.58498500	0.83479300

**Benzene**			
Single-point energy (Eh)		-232.185748	
Gibbs free energy (Eh)		-232.1124603	
**Atom**	**X**	**Y**	**Z**
C	-2.43931700	0.37860600	-0.03189100
C	-1.04687000	0.37857600	-0.03145300
C	-0.35066100	1.58425500	-0.03199900
C	-1.04682900	2.79017400	-0.03299400
C	-2.43912800	2.79020000	-0.03343200
C	-3.13540200	1.58438300	-0.03288500
H	-2.98247100	-0.56220300	-0.03147900
H	-0.50386800	-0.56232700	-0.03065600
H	0.73566700	1.58433400	-0.03166700
H	-0.50342000	3.73083500	-0.03340700
H	-2.98240100	3.73094600	-0.03422800
H	-4.22173100	1.58454600	-0.03321700

**Water**			
Single-point energy (Eh)		-76.42412728	
Gibbs free energy (Eh)		-76.42040848	
**Atom**	**X**	**Y**	**Z**
O	-10.12344800	-0.12948100	0.00000000

H	-9.16276700	-0.08375400	0.00000000
H	-10.40103700	0.79137400	0.00000000

**Int-1**			
Single-point energy (Eh)		-616.8166288	
Gibbs free energy (Eh)		-616.6598947	
**Atom**	**X**	**Y**	**Z**
C	-0.83042700	-0.43069900	-0.02057300
C	0.73533400	-0.32566600	-0.08791400
B	-0.17253500	1.78135800	0.00487800
O	0.97656800	1.03088500	0.38541700
O	-1.26668100	0.93523000	-0.28848000
C	-1.34130800	-0.73885800	1.38713800
H	-2.42669300	-0.60838800	1.40668200
H	-1.11015300	-1.77275100	1.66242900
H	-0.89584300	-0.06778700	2.12805400
C	1.46660900	-1.28107100	0.84478000
H	1.21120600	-2.31528800	0.59132300
H	2.54599300	-1.15300700	0.72339700
H	1.21048300	-1.09812000	1.89950000
C	1.29640600	-0.44487700	-1.50303200
H	2.34644800	-0.13875400	-1.49316800
H	1.23966500	-1.47880900	-1.85596100
H	0.75701700	0.19753600	-2.20570900
C	-1.44793000	-1.36784700	-1.04118200
H	-1.06594700	-2.38383700	-0.89524300
H	-2.53417400	-1.38833100	-0.91386400
H	-1.22432700	-1.05150400	-2.06218400
O	-0.49725100	2.99522300	0.71718200
N	0.52148700	4.00596100	0.57496700
N	0.20720700	2.17787600	-1.21469100
O	0.29885500	4.92154300	1.26580500

**Int-2**			
Single-point energy (Eh)		-616.893891	
Gibbs free energy (Eh)		-616.7296192	
**Atom**	**X**	**Y**	**Z**
C	-3.52235900	-0.63998500	0.18603200
C	-1.96221600	-0.81570600	0.12358000
B	-2.48717300	1.36592800	0.40337500
O	-1.48018000	0.47326800	0.64098600
O	-3.67574400	0.81265200	-0.00288200
C	-4.10767500	-0.96427500	1.55550000
H	-5.14911900	-0.63094400	1.58378900
H	-4.08456100	-2.04135100	1.74623600
H	-3.55925200	-0.45473800	2.35403300
C	-1.41545700	-1.92424000	1.00330300

H	-1.83326900	-2.88895500	0.69641400
H	-0.32787400	-1.97329100	0.89586400
H	-1.65147700	-1.75632200	2.05597100
C	-1.43165000	-0.94402100	-1.30010700
H	-0.34098000	-0.86503000	-1.27872400
H	-1.70041800	-1.91222400	-1.73293000
H	-1.82215000	-0.15041100	-1.94518600
C	-4.28727300	-1.35547000	-0.91085000
H	-4.11268400	-2.43465700	-0.84665000
H	-5.35955400	-1.17624800	-0.78752200
H	-3.98964100	-1.00963000	-1.90280600
O	-2.27841300	2.72188500	0.54131100
N	-3.51298700	3.52139400	0.45090600
N	-4.07889100	3.16346900	-0.33274900
O	-3.35781600	4.73370100	0.58408700

**Int-4**			
Single-point energy (Eh)		-1027.525994	
Gibbs free energy (Eh)		-1027.201943	
**Atom**	**X**	**Y**	**Z**
N	-1.14098000	-0.50061100	-0.56724200
O	-1.93857900	0.28884800	-1.36955200
O	-0.13570200	-0.87502600	-1.43468300
B	0.85525300	-1.65141900	-0.85635300
O	1.82718900	-2.18696700	-1.65773400
O	0.97318200	-1.94043400	0.47449900
C	2.84199100	-2.71705600	-0.74749400
C	2.01490700	-2.96503000	0.56560000
C	3.44457500	-3.96583500	-1.36501600
C	3.90070800	-1.63010400	-0.58785500
C	1.30094700	-4.31325700	0.57698100
C	2.78651500	-2.75649800	1.85590100
H	3.99239700	-3.69832900	-2.27328400
H	4.14732500	-4.43403500	-0.66730600
H	2.67507700	-4.69270500	-1.63170100
H	4.73503500	-1.97548100	0.03040900
H	4.28701300	-1.36647200	-1.57645100
H	3.47677100	-0.72839600	-0.13499200
H	2.00780800	-5.13766600	0.71269600
H	0.58821700	-4.32600600	1.40630600
H	0.74578500	-4.47500100	-0.35224400
H	2.12865700	-2.94397000	2.70947100
H	3.62929000	-3.45391100	1.91451700
H	3.16674100	-1.73641700	1.93590600
B	-3.11710200	0.70212200	-0.77011900
O	-3.58380400	0.29115700	0.44726400
O	-3.92155800	1.58854600	-1.43415800
C	-4.73162800	1.15042100	0.74142100

C	-5.18232400	1.60691100	-0.69287700
C	-5.76112400	0.33738800	1.50552600
C	-4.21136900	2.30090600	1.59752700
C	-6.10677200	0.60217900	-1.37447800
C	-5.76829700	3.00547700	-0.76172900
H	-5.35685800	0.06291400	2.48413900
H	-6.67085500	0.92644700	1.66428100
H	-6.02297700	-0.57967300	0.97448400
H	-5.02612400	2.95706200	1.91913600
H	-3.72618600	1.88733800	2.48607100
H	-3.47347300	2.89853000	1.05309400
H	-7.10100900	0.60154000	-0.91703600
H	-6.20968500	0.87696400	-2.42803400
H	-5.69638500	-0.41127100	-1.32463600
H	-6.05349600	3.23305200	-1.79294300
H	-6.66497700	3.07297400	-0.13616400
H	-5.05009600	3.75837100	-0.43172200

**Int-5**			
Single-point energy (Eh)		-1027.521127	
Gibbs free energy (Eh)		-1027.19578	
**Atom**	**X**	**Y**	**Z**
N	-0.22998700	0.75500600	1.83521300
O	-0.66602400	1.08830200	0.48004600
O	0.48235200	1.58069300	2.40682200
B	-0.70695900	-0.53503900	2.33150900
O	-1.44634700	-1.37896800	1.55198700
O	-0.45949600	-0.95994100	3.60242100
C	-1.58332200	-2.61121400	2.33519600
C	-1.37249400	-2.09329600	3.80163300
C	-2.95289800	-3.20386100	2.05874100
C	-0.47888100	-3.55284400	1.86774300
C	-2.64447000	-1.51324900	4.41311200
C	-0.71832500	-3.08639300	4.74256500
H	-3.00818600	-3.52729800	1.01508600
H	-3.12594200	-4.07746800	2.69622300
H	-3.74708000	-2.47562100	2.23631100
H	-0.55624100	-4.52819200	2.35803000
H	-0.57174700	-3.69856600	0.78790800
H	0.51096600	-3.13383000	2.07277600
H	-3.36484900	-2.30062100	4.65615000
H	-2.38251900	-0.98592700	5.33461700
H	-3.11484600	-0.79608800	3.73202400
H	-0.61353600	-2.63712000	5.73414900
H	-1.34051000	-3.98292000	4.83714500
H	0.27335200	-3.37966800	4.39285800
B	-2.01857700	1.33534700	0.40539400
O	-2.86835800	1.35040200	1.48743500

O	-2.62368400	1.58344600	-0.79883000
C	-4.15095800	1.83702600	0.98389900
C	-4.05926000	1.48083800	-0.54239000
C	-5.26441200	1.13236800	1.73799500
C	-4.18912000	3.33947300	1.24388300
C	-4.45221700	0.03474200	-0.83537500
C	-4.79210700	2.43678700	-1.46537900
H	-5.24337600	1.43487700	2.78942700
H	-6.24076900	1.40651800	1.32396500
H	-5.15474700	0.04680700	1.68952700
H	-5.15234400	3.76868200	0.95113100
H	-4.03923900	3.51764100	2.31248700
H	-3.39315700	3.85380200	0.69673400
H	-5.53081600	-0.11673100	-0.72639300
H	-4.17023900	-0.20399300	-1.86474200
H	-3.92516000	-0.65654300	-0.16897300
H	-4.67130200	2.10975000	-2.50220600
H	-5.86258600	2.44555300	-1.23266800
H	-4.40415400	3.45372300	-1.38066800

**Int-b**			
Single-point energy (Eh)		-616.8852212	
Gibbs free energy (Eh)		-616.7215135	
**Atom**	**X**	**Y**	**Z**
**Atom**	**X**	**Y**	**Z**
C	-0.95905700	-0.53794300	0.00234100
C	0.56287100	-0.21190100	-0.20363800
B	-0.66668500	1.70377800	-0.12064500
O	0.60410700	1.22920300	0.12768200
O	-1.60582800	0.73998900	-0.33523500
C	-1.31519100	-0.83957800	1.45335400
H	-2.40429500	-0.85971800	1.55085100
H	-0.92257800	-1.81379600	1.75964700
H	-0.92265600	-0.07385200	2.12997600
C	1.50534100	-0.94800400	0.72688300
H	1.41185300	-2.02796300	0.57170200
H	2.53823200	-0.66089900	0.50888400
H	1.29477600	-0.72345400	1.77417400
C	1.01523400	-0.33599200	-1.65295100
H	2.01541800	0.09628100	-1.75240500
H	1.06403800	-1.38561100	-1.95707200
H	0.33842700	0.19372200	-2.33096600
C	-1.50087200	-1.61175300	-0.92191700
H	-0.96353900	-2.55223400	-0.76063500
H	-2.55927000	-1.78109500	-0.70365500
H	-1.40571600	-1.32588700	-1.97133600
O	-0.99801600	3.04115400	-0.17032300
N	-0.05524700	4.07993900	-0.02065400

N	1.14129500	3.76945000	-0.59480900
H	1.32925400	2.80769500	-0.49070200

**Int-b configuration 2**			
Single-point energy (Eh)		-616.8927241	
Gibbs free energy (Eh)		-616.7286625	
**Atom**	**X**	**Y**	**Z**
C	-0.84404600	-0.45280100	0.04299500
C	0.72066500	-0.32630200	-0.01078200
B	-0.22123100	1.70384900	0.29760000
O	0.94182400	1.02571900	0.52625100
O	-1.27453200	0.94196000	-0.12522400
C	-1.36004600	-0.90917300	1.40339200
H	-2.44614600	-0.78189400	1.42981500
H	-1.13082300	-1.96492300	1.57679800
H	-0.92358100	-0.31710100	2.21418200
C	1.46869700	-1.32083300	0.85645000
H	1.24570200	-2.34303800	0.53268000
H	2.54578500	-1.15760600	0.75547700
H	1.20037000	-1.21868100	1.90985500
C	1.27327300	-0.32413300	-1.43172100
H	2.32708400	0.03283900	-1.39936300
H	1.20266300	-1.31790600	-1.88431600
H	0.73752500	0.38926300	-2.06613100
C	-1.45138700	-1.28627200	-1.06964000
H	-1.06978100	-2.31167700	-1.02213300
H	-2.53841500	-1.32014500	-0.95053900
H	-1.22401100	-0.87117100	-2.05354500
O	-0.41032100	3.06755500	0.46499100
N	0.73475300	3.76879300	0.78595900
O	0.25680100	5.05027300	0.92763700
H	1.05552300	5.55537000	1.14904200

**NO_2**			
Single-point energy (Eh)		-205.0520117	
Gibbs free energy (Eh)		-205.0680747	
**Atom**	**X**	**Y**	**Z**
N	-4.35729800	1.53581600	0.02010500
O	-4.97609800	2.60760900	0.02010500
O	-3.11969800	1.53581600	0.02010500

**NO**			
Single-point energy (Eh)		-129.8635711	
Gibbs free energy (Eh)		-129.8805722	
**Atom**	**X**	**Y**	**Z**
N	-4.35729800	1.53581600	0.02010500

O	-4.97609800	2.60760900	0.02010500

O(H)NO			
Single-point energy (Eh)		-205.6839738	
Gibbs free energy (Eh)		-205.6853922	
**Atom**	**X**	**Y**	**Z**
N	-0.24644100	0.91905200	0.00000000
O	-0.92929800	1.92632100	0.00000000
O	0.96736700	0.83132400	0.00000000
H	-0.76960200	0.01294100	0.00000000

HONO			
Single-point energy (Eh)		-205.6903869	
Gibbs free energy (Eh)		-205.6945808	
**Atom**	**X**	**Y**	**Z**
N	-0.36103700	0.84487500	0.00000000
O	-0.85864800	1.89707900	0.00000000
O	1.09112300	0.95647700	0.00000000
H	1.36818800	0.02584800	0.00000000

HNO_2 Radical Cation			
Single-point energy (Eh)		-205.24154	
Gibbs free energy (Eh)		-205.2473002	
**Atom**	**X**	**Y**	**Z**
N	-2.72241533401987	0.23443415903682	-0.00000000790596
H	-3.24986306533126	-0.67902132808910	0.00000003089550
O	-3.26885480891813	1.33037501254242	0.00000002407920
O	-1.50028127173073	0.30922708650986	0.00000002408490

HNO_2 Radical Anion			
Single-point energy (Eh)		-205.6755606	
Gibbs free energy (Eh)		-205.6819139	
**Atom**	**X**	**Y**	**Z**
N	-2.70494628081661	0.26476007466280	-0.00000003508507
H	-3.21653687034689	-0.62128983999133	0.00000001246025
O	-3.42376194025398	1.36109731958288	0.00000011312550
O	-1.39616938858250	0.19044737574566	0.00000011312260

TS-1	
Single-point energy (Eh)	-616.8063355
Gibbs free energy (Eh)	-616.6486378

Atom	X	Y	Z
C	-0.76360000	-0.38255300	-0.04807200
C	0.80210900	-0.40557700	-0.13760400
B	0.06966500	1.76648300	-0.17387000
O	1.14916700	0.94946600	0.25484800
O	-1.07928700	0.99041800	-0.42259700
C	-1.28118800	-0.57340500	1.37729000
H	-2.35008900	-0.34288600	1.39994600
H	-1.14359800	-1.60992400	1.70242900
H	-0.76736400	0.09033300	2.07904700
C	1.48362100	-1.37362200	0.81580700
H	1.15409700	-2.39664400	0.60499700
H	2.56756600	-1.32628600	0.67656800
H	1.25607300	-1.13570400	1.85678600
C	1.31603300	-0.61170100	-1.56476400
H	2.38936900	-0.40332400	-1.58845800
H	1.15496400	-1.64622600	-1.88251900
H	0.81453200	0.05823000	-2.26946000
C	-1.46413200	-1.33377100	-1.00289900
H	-1.16742200	-2.36689600	-0.79250100
H	-2.54700800	-1.25467600	-0.86858800
H	-1.22721900	-1.10289100	-2.04350600
O	-0.24198100	2.84215900	1.10614700
N	-0.13841200	4.04436100	0.62143300
O	0.35760100	2.58161100	-1.06807100
O	-0.34348500	4.94304600	1.36760900

TS-2			
Single-point energy (Eh)		-616.8117307	
Gibbs free energy (Eh)		-616.6574346	
**Atom**	**X**	**Y**	**Z**
C	-0.83774900	-0.46506200	0.05074600
C	0.72887300	-0.35016600	-0.02014100
B	-0.19305600	1.70964100	0.25450700
O	0.96434100	0.98770400	0.51746700
O	-1.26948500	0.92230200	-0.10550400
C	-1.33869000	-0.91172800	1.42196700
H	-2.42318900	-0.77766300	1.46225900
H	-1.11210200	-1.96720700	1.60220700
H	-0.89043900	-0.31297300	2.22084400
C	1.47765300	-1.34769000	0.84792800
H	1.24351900	-2.37140400	0.53673300
H	2.55473100	-1.19432100	0.73506500
H	1.22409900	-1.23248700	1.90329000
C	1.27414100	-0.37782000	-1.44556900
H	2.32317600	-0.06889700	-1.42692400
H	1.21612900	-1.38391600	-1.87224100
H	0.72794600	0.31071600	-2.09732900

C	-1.46430500	-1.30244500	-1.04937200
H	-1.09458500	-2.33237200	-1.00137800
H	-2.55031400	-1.32345800	-0.92091600
H	-1.24513400	-0.89551100	-2.03840500
O	-0.40963900	3.00824100	0.79397400
N	0.48035100	3.96047700	0.15362800
O	0.30878800	2.38223200	-1.16149600
O	0.36602400	5.02855700	0.60090800

**TS-3**			
Single-point energy (Eh)		-616.8619636	
Gibbs free energy (Eh)		-616.7025178	
**Atom**	**X**	**Y**	**Z**
C	-0.63903500	-0.33994400	-0.02566800
C	0.91430400	-0.55775100	-0.06261600
B	0.44176700	1.67071800	0.07885200
O	1.41993700	0.74026200	0.37946600
O	-0.75355900	1.08660300	-0.32117000
C	-1.23692900	-0.55631200	1.36181200
H	-2.26999900	-0.19600300	1.36092900
H	-1.24109000	-1.61675600	1.63218000
H	-0.67849500	-0.00275200	2.12358400
C	1.42752800	-1.62968300	0.88253400
H	0.98491600	-2.59966400	0.63096900
H	2.51412400	-1.71476400	0.78531800
H	1.19219200	-1.39399700	1.92243800
C	1.44890500	-0.78771700	-1.47344100
H	2.54107000	-0.72910100	-1.45019600
H	1.16337400	-1.77518800	-1.84908700
H	1.07782600	-0.02656200	-2.16724700
C	-1.41585900	-1.12301900	-1.06910700
H	-1.26552200	-2.19833000	-0.92463000
H	-2.48482100	-0.91210000	-0.96634100
H	-1.10748700	-0.85825600	-2.08275300
O	0.64578500	3.00907000	0.15846300
N	-1.06983200	3.89213700	0.51651300
O	-0.71047200	3.69126300	-0.47789700
O	-0.89862900	4.92784700	1.03709500

**TS-4**			
Single-point energy (Eh)		-616.8619636	
Gibbs free energy (Eh)		-616.7025178	
**Atom**	**X**	**Y**	**Z**
N	0.21073200	0.61104400	0.19012300
O	-0.24550900	1.74649600	-0.43644300
O	1.51174000	0.51549900	0.04379900
B	1.86793800	-0.98979500	0.09116600

O	2.65187000	-1.42191600	-0.98857400
O	2.40261900	-1.39815000	1.32040800
C	3.89330200	-1.92693900	-0.41329100
C	3.43086900	-2.38247600	1.01255800
C	4.42530100	-3.04036900	-1.29802800
C	4.88047900	-0.76194000	-0.36599300
C	2.77774700	-3.76339500	1.00695800
C	4.50500400	-2.30954200	2.08453000
H	4.73324800	-2.62675800	-2.26345600
H	5.29958700	-3.51025700	-0.83409100
H	3.66721500	-3.80588800	-1.47758200
H	5.86850300	-1.08733900	-0.02454500
H	4.98167600	-0.34737300	-1.37349800
H	4.52176400	0.03084300	0.29821400
H	3.52043800	-4.55247900	0.85094900
H	2.29577600	-3.93081800	1.97504000
H	2.01686600	-3.83813600	0.22380500
H	4.08615500	-2.62654500	3.04478800
H	5.33662200	-2.97868200	1.83762400
H	4.89013400	-1.29383200	2.19595000
B	-1.63233100	1.90472000	-0.36938500
O	-2.49173500	0.98004400	0.14024200
O	-2.18959300	3.04949900	-0.84815600
C	-3.79436300	1.66936000	0.22200300
C	-3.64201100	2.79348600	-0.86540500
C	-4.89267700	0.66150000	-0.05460000
C	-3.90295600	2.21640200	1.64011800
C	-3.98187300	2.31613100	-2.27216100
C	-4.36409400	4.08726600	-0.54326700
H	-4.92807300	-0.07199300	0.75618300
H	-5.86265100	1.16761300	-0.10007200
H	-4.72749200	0.13159400	-0.99482900
H	-4.88205600	2.67485100	1.80831000
H	-3.78214200	1.39038900	2.34677000
H	-3.12683200	2.96114600	1.84299900
H	-5.06036300	2.17205500	-2.38654600
H	-3.65608500	3.07372200	-2.99052900
H	-3.47523700	1.37507300	-2.50883300
H	-4.20581100	4.80653700	-1.35215300
H	-5.43999900	3.90318800	-0.45495200
H	-4.00435700	4.52846600	0.38827500
H	-0.02959400	-0.62431800	-0.02013100
H	0.54225000	-1.47798100	-0.08429000

**TS-4’**			
Single-point energy (Eh)		-1028.636065	
Gibbs free energy (Eh)		-1028.2971	
**Atom**	**X**	**Y**	**Z**

N	0.21073200	0.61104400	0.19012300
O	-0.24550900	1.74649600	-0.43644300
O	1.51174000	0.51549900	0.04379900
B	1.86793800	-0.98979500	0.09116600
O	2.65187000	-1.42191600	-0.98857400
O	2.40261900	-1.39815000	1.32040800
C	3.89330200	-1.92693900	-0.41329100
C	3.43086900	-2.38247600	1.01255800
C	4.42530100	-3.04036900	-1.29802800
C	4.88047900	-0.76194000	-0.36599300
C	2.77774700	-3.76339500	1.00695800
C	4.50500400	-2.30954200	2.08453000
H	4.73324800	-2.62675800	-2.26345600
H	5.29958700	-3.51025700	-0.83409100
H	3.66721500	-3.80588800	-1.47758200
H	5.86850300	-1.08733900	-0.02454500
H	4.98167600	-0.34737300	-1.37349800
H	4.52176400	0.03084300	0.29821400
H	3.52043800	-4.55247900	0.85094900
H	2.29577600	-3.93081800	1.97504000
H	2.01686600	-3.83813600	0.22380500
H	4.08615500	-2.62654500	3.04478800
H	5.33662200	-2.97868200	1.83762400
H	4.89013400	-1.29383200	2.19595000
B	-1.63233100	1.90472000	-0.36938500
O	-2.49173500	0.98004400	0.14024200
O	-2.18959300	3.04949900	-0.84815600
C	-3.79436300	1.66936000	0.22200300
C	-3.64201100	2.79348600	-0.86540500
C	-4.89267700	0.66150000	-0.05460000
C	-3.90295600	2.21640200	1.64011800
C	-3.98187300	2.31613100	-2.27216100
C	-4.36409400	4.08726600	-0.54326700
H	-4.92807300	-0.07199300	0.75618300
H	-5.86265100	1.16761300	-0.10007200
H	-4.72749200	0.13159400	-0.99482900
H	-4.88205600	2.67485100	1.80831000
H	-3.78214200	1.39038900	2.34677000
H	-3.12683200	2.96114600	1.84299900
H	-5.06036300	2.17205500	-2.38654600
H	-3.65608500	3.07372200	-2.99052900
H	-3.47523700	1.37507300	-2.50883300
H	-4.20581100	4.80653700	-1.35215300
H	-5.43999900	3.90318800	-0.45495200
H	-4.00435700	4.52846600	0.38827500
H	-0.02959400	-0.62431800	-0.02013100
H	0.54225000	-1.47798100	-0.08429000

TS-5			
Single-point energy (Eh)		-1027.490321	
Gibbs free energy (Eh)		-1027.165967	
**Atom**	**X**	**Y**	**Z**
N	1.64926800	2.18273500	0.06939000
O	0.63547300	2.72702200	-0.74082000
O	2.83809700	2.28691200	-0.47332400
B	2.25757200	0.77040000	0.02364600
O	1.88072600	-0.09707300	-0.97543300
O	2.84536600	0.18041300	1.11798900
C	2.46231500	-1.39289500	-0.60645300
C	2.60843700	-1.25722900	0.95136700
C	1.51944800	-2.49053500	-1.06260300
C	3.80333900	-1.49476000	-1.32561800
C	1.31846200	-1.58458400	1.69819400
C	3.78177800	-2.01437800	1.54472300
H	1.48371100	-2.51247100	-2.15602900
H	1.87620500	-3.46488000	-0.71213500
H	0.50709600	-2.32868000	-0.68568900
H	4.27309600	-2.46757700	-1.14989300
H	3.63679800	-1.38036700	-2.40073900
H	4.48992100	-0.70793200	-0.99763900
H	1.10672700	-2.65805700	1.67027400
H	1.43199600	-1.28139400	2.74334500
H	0.46841200	-1.04136700	1.27342900
H	3.80210600	-1.86700000	2.62872900
H	3.67668100	-3.08663200	1.34722900
H	4.73212200	-1.67068400	1.13125600
B	-0.62027500	2.19166800	-0.47098400
O	-0.85792000	1.18795800	0.42318900
O	-1.71030200	2.65960600	-1.14327600
C	-2.32341300	1.07715600	0.51055400
C	-2.78195300	1.68935600	-0.86147800
C	-2.68445400	-0.38324600	0.70640900
C	-2.75170000	1.90906300	1.71293800
C	-2.76031200	0.67846100	-2.00249900
C	-4.10548000	2.42792400	-0.81632000
H	-2.33050400	-0.72054100	1.68544100
H	-3.77191000	-0.50875300	0.67504700
H	-2.23398000	-1.01550000	-0.06183200
H	-3.82959800	1.82781400	1.88210100
H	-2.23292200	1.53829000	2.60173400
H	-2.49557800	2.96474300	1.57838200
H	-3.57138700	-0.04890600	-1.90026000
H	-2.89132800	1.21166400	-2.94846200
H	-1.80738300	0.13993500	-2.03992000
H	-4.34014100	2.81974700	-1.81038800
H	-4.90694800	1.72426200	-0.52106400

H	-4.07766300	3.26211100	-0.11250600

TS-6			
Single-point energy (Eh)		-1027.50596	
Gibbs free energy (Eh)		-1027.181848	
**Atom**	**X**	**Y**	**Z**
N	-1.67893600	1.10718500	3.65085700
O	-1.26032900	0.99197300	1.96337600
O	-0.98224500	1.83135300	4.29066900
B	-1.61232400	-0.39501000	3.46356300
O	-2.72977500	-1.11081300	3.15137900
O	-0.50702300	-1.11344900	3.79435700
C	-2.23317500	-2.47589700	2.91589800
C	-0.94753400	-2.51651000	3.81802900
C	-3.32132700	-3.45592800	3.31049000
C	-1.91789700	-2.57904800	1.42735200
C	-1.24776500	-2.85140500	5.27458300
C	0.17906200	-3.38057500	3.28419700
H	-4.17475700	-3.34442800	2.63537600
H	-2.95340100	-4.48426400	3.22811000
H	-3.66670700	-3.28542600	4.33190700
H	-1.58033700	-3.58657000	1.16560600
H	-2.82491900	-2.36032600	0.85722500
H	-1.15191000	-1.85585700	1.13115000
H	-1.51510500	-3.90614300	5.39232100
H	-0.35509900	-2.65150200	5.87383300
H	-2.06572700	-2.23593400	5.66165000
H	1.02727000	-3.34204600	3.97368600
H	-0.14733600	-4.42305700	3.20269500
H	0.51810000	-3.03789000	2.30490800
B	-2.34395200	1.31382500	1.19467900
O	-3.49157100	1.92155600	1.65935500
O	-2.36784900	1.05546800	-0.16127000
C	-4.26494000	2.29963700	0.48435500
C	-3.74011100	1.28872700	-0.59505500
C	-5.74323000	2.17557000	0.81173500
C	-3.90722600	3.74859000	0.16390400
C	-4.45710100	-0.05835800	-0.53405500
C	-3.72174800	1.82220500	-2.01630300
H	-6.01425600	2.92055500	1.56556000
H	-6.35164400	2.35720600	-0.08103500
H	-5.98317600	1.18710600	1.20845500
H	-4.50069500	4.13137900	-0.67247400
H	-4.11130600	4.36420100	1.04464200
H	-2.84544700	3.84746600	-0.83027000
H	-5.48170400	0.01451800	-0.91244700

H	-3.90924800	-0.77561600	-1.15274900
H	-4.48560200	-0.43722900	0.49312800
H	-3.34094300	1.05100400	-2.69253900
H	-4.73402900	2.08950200	-2.33898700
H	-3.07959900	2.70064600	-2.10466900

**TS-7**			
Single-point energy (Eh)		-438.4067352	
Gibbs free energy (Eh)		-438.3161291	
**Atom**	**X**	**Y**	**Z**
C	-1.34112700	1.03273000	0.33719500
C	0.14063000	1.06763400	0.14900900
C	0.82014500	2.21626000	-0.02198900
C	0.15338000	3.51615900	0.01826000
C	-1.30675300	3.49692000	-0.02159300
C	-2.00387200	2.35818100	0.14912200
H	-1.79092900	0.28529300	-0.33710700
H	0.66751200	0.11636000	0.13987900
H	1.89842800	2.19735400	-0.16000400
H	-1.82876400	4.44076500	-0.15810100
H	-3.09106900	2.37869400	0.14126900
H	0.42643300	3.98039200	1.18238200
H	-1.57705200	0.64165200	1.34452600
N	0.67748700	4.45793200	2.48382000
O	-0.24310000	5.04138100	3.02095700
O	1.76974000	4.21151500	2.95565300
H	0.63318400	4.31240400	-0.56004800

**TS-7’**			
Single-point energy (Eh)		-438.4041189	
Gibbs free energy (Eh)		-438.3133297	
**Atom**	**X**	**Y**	**Z**
C	-2.10535100	-0.99104900	-2.26963100
C	-0.77883000	-0.78479800	-2.28243200
C	-0.17441500	0.57868200	-2.19794100
C	-1.17825400	1.68455900	-2.24379500
C	-2.50133000	1.46312000	-2.22683500
C	-3.07015100	0.10957800	-2.11899200
H	-2.49771200	-2.00313200	-2.34037500
H	-0.09821200	-1.62842200	-2.37460500
H	0.42896300	0.66106300	-1.27550900
H	-0.79799800	2.70197200	-2.30458500
H	-3.19514100	2.30002700	-2.25760300
H	-3.99850600	-0.03038000	-2.68521000
H	-3.50736800	0.00925400	-0.99188000
H	0.56866000	0.71066000	-3.00190200
O	-3.96194600	-0.15174700	0.38342700

O	-4.92462700	0.60237900	0.64759500
O	-5.41130700	1.34518100	-0.17124600

**TS-8**			
Single-point energy (Eh)		-411.3542428	
Gibbs free energy (Eh)		-411.3454865	
**Atom**	**X**	**Y**	**Z**
N	-1.36725300	0.01823600	-0.00036300
N	1.36725900	-0.01823700	0.00035600
O	0.80320900	-1.18395500	0.00073800
O	-0.80320100	1.18395300	-0.00079600
O	2.55696800	0.08475600	0.00068300
O	-2.55696100	-0.08476000	-0.00052300
H	0.43684300	0.83948000	-0.00032900
H	-0.43686400	-0.83947300	0.00023300

**TS-9**			
Single-point energy (Eh)		-438.4174247	
Gibbs free energy (Eh)		-438.3302947	
**Atom**	**X**	**Y**	**Z**
C	-0.98872000	1.13417000	0.87791800
C	0.38980600	1.34670900	0.86748700
C	0.90759200	2.48635800	0.27392200
C	0.03297200	3.46129800	-0.26303300
C	-1.35746800	3.19319500	-0.31155500
C	-1.86135800	2.05185000	0.28617000
H	1.05609500	0.61688900	1.31685800
H	1.97753700	2.66839400	0.26557900
H	-2.02311100	3.91944300	-0.76831000
H	-2.93031700	1.86199200	0.28646500
H	0.01652500	4.32005300	0.87813800
H	-1.38981000	0.24052100	1.34796500
H	-0.42421800	4.91667400	2.61546900
N	2.02343000	4.13260200	2.86468500
O	2.98076800	4.57672700	2.38649200
O	0.13875700	5.35038600	1.95863700
H	0.44253300	4.25619500	-0.88702600

**TS-a**			
Single-point energy (Eh)		-616.81361	
Gibbs free energy (Eh)		-616.6612594	
**Atom**	**X**	**Y**	**Z**
C	-0.78872200	-0.51444200	-0.01369100
C	0.77132900	-0.34845200	0.01148600
B	-0.22979700	1.67869400	-0.20864300
O	0.92622700	1.09146200	0.24550700

O	-1.22958800	0.79113000	-0.51721200
C	-1.39655000	-0.67690000	1.37574200
H	-2.48396900	-0.58827600	1.29706400
H	-1.15869400	-1.65845600	1.79684200
H	-1.03696600	0.09664800	2.06181900
C	1.47349400	-1.10090900	1.12657500
H	1.29601400	-2.17691600	1.02524500
H	2.55176900	-0.92582900	1.06413900
H	1.12716700	-0.77800700	2.11043200
C	1.42606800	-0.64565500	-1.33386800
H	2.47014800	-0.32125700	-1.29705500
H	1.40457900	-1.71693800	-1.55557600
H	0.92608400	-0.10904800	-2.14656800
C	-1.29836900	-1.59448800	-0.94990700
H	-0.90530900	-2.57117100	-0.64789800
H	-2.39062100	-1.63600100	-0.90107800
H	-1.00619800	-1.40142700	-1.98404600
O	-0.40088100	3.04393500	-0.37746200
N	1.01402900	3.86295500	-0.04161000
O	0.91651900	4.94057100	-0.39949300
H	-1.97776400	3.25877800	0.25920400

**TS-b**			
Single-point energy (Eh)		-616.8084919	
Gibbs free energy (Eh)		-616.6502437	
**Atom**	**X**	**Y**	**Z**
C	0.45040900	0.79692100	0.14391600
C	-0.76863800	-0.20132400	0.20744000
B	1.20372700	-1.42099100	0.02526200
O	1.50729400	-0.03136100	-0.34212600
O	-0.11438700	-1.43694600	0.55507800
C	0.88741100	1.25584000	1.55365600
H	1.85704500	1.75457700	1.48060300
H	0.14982000	1.96430500	1.94188600
H	0.96983400	0.40077800	2.22837800
C	-1.79403500	0.13373500	1.27744600
H	-2.21231200	1.13275700	1.11251300
H	-2.61378600	-0.58955500	1.22753800
H	-1.35927600	0.08880600	2.27801600
C	-1.45473000	-0.34214900	-1.15273100
H	-2.14584600	1.18916900	-1.10308400
H	-2.02917000	0.55541400	-1.40364800
H	-0.73376000	-0.53354600	-1.95252700
C	0.29226400	1.99638400	-0.77552400
H	-0.58064600	2.58148900	-0.46775800
H	1.17584600	2.63851800	-0.71469100
H	0.15930900	1.68227600	-1.81290900
O	2.06972700	-1.94612900	0.71963900

O	1.29474500	-2.10163100	-1.38228900
N	1.84525200	-3.30076100	-1.31150600
O	1.94390400	-3.88823500	-2.33257800

**TS-c**			
Single-point energy (Eh)		-616.8084557	
Gibbs free energy (Eh)		-616.6502438	
**Atom**	**X**	**Y**	**Z**
C	-0.75672000	-0.35323900	0.05568000
C	0.81035700	-0.38258500	-0.00920400
B	0.09763800	1.74444700	0.32280500
O	1.16690200	0.91099400	0.58356700
O	-1.05512100	1.07900100	-0.08088100
C	-1.30277400	-0.77032400	1.41787000
H	-2.36948200	-0.53234200	1.45959600
H	-1.18200900	-1.84689000	1.57422300
H	-0.79606500	-0.23763800	2.22883200
C	1.45988400	-1.48158000	0.81192900
H	1.14523900	-2.46293300	0.44132700
H	2.54763600	-1.41296400	0.71713000
H	1.19917200	-1.40294600	1.86921900
C	1.35059300	-0.38797100	-1.43642000
H	2.42749700	-0.19814500	-1.40735800
H	1.18350000	-1.35736100	-1.91559600
H	0.87859000	0.39096800	-2.04323100
C	-1.45145200	-1.10976500	-1.06064700
H	-1.15514000	-2.16404500	-1.04040800
H	-2.53542900	-1.05724700	-0.92225000
H	-1.20678800	-0.69404800	-2.04022700
O	-0.01064600	2.93370700	1.23741700
N	-0.23479800	4.08692600	0.64206100
O	-0.45126500	3.95608300	-0.57321900
H	0.24068000	2.74989500	-0.83221200

**TS-c’**			
Single-point energy (Eh)		-1028.6129	
Gibbs free energy (Eh)		-1028.273619	
**Atom**	**X**	**Y**	**Z**
C	0.03958900	-1.98892600	-2.33019500
C	1.41643500	-2.56336600	-1.83775200
B	1.26772600	-0.36009000	-1.35702500
O	1.89507200	-1.50009400	-0.94291800
O	0.27174700	-0.53923600	-2.27856400
C	-1.10576000	-2.28239400	-1.36708700
H	-1.97515000	-1.68586500	-1.65945800
H	-1.38556600	-3.33982800	-1.39757400
H	-0.83597400	-2.01908200	-0.33964600

C	1.31920900	-3.85410100	-1.04565200
H	0.87412300	-4.64185300	-1.66282300
H	2.32138200	-4.17726700	-0.74882700
H	0.71568400	-3.72823300	-0.14462600
H	2.44575300	-2.69306100	-2.95490100
H	3.42331400	-2.90251200	-2.51112100
H	2.18881000	-3.51441900	-3.63053200
H	2.52232200	-1.76943100	-3.53740900
C	-0.33775700	-2.37096900	-3.74879200
H	-0.42372700	-3.45936100	-3.83502000
H	-1.30690500	-1.93014600	-4.00071600
H	0.40157600	-2.01785100	-4.47038300
O	1.54147300	0.92473900	-0.91520800
N	2.62065100	1.01832900	-0.03824900
O	2.39657200	2.18083600	0.64270500
H	2.68922200	2.08035900	1.88726100
C	-0.90007500	2.89996100	1.77211500
C	-0.91933300	1.38181200	2.17287200
B	1.21214100	2.10667000	1.91167400
H	2.25912400	2.07252800	2.78538400
O	0.49301500	3.27440300	2.05526000
O	0.45112300	0.95724800	1.86562500
C	-1.81204300	3.78317800	2.60336300
H	-1.74000000	4.81714900	2.25277700
H	-2.85224500	3.45763800	2.49389600
H	-1.54383100	3.75647800	3.66155800
C	-1.13164300	1.15937200	3.66715000
H	-2.16204600	1.38420300	3.95948300
H	-0.92847400	0.10963900	3.89862300
H	-0.45404800	1.78186700	4.25949800
C	-1.87499000	0.52935900	1.35838800
H	-1.83064200	-0.50710000	1.70728700
H	-2.90238200	0.88845200	1.48208100
H	-1.61818400	0.54894100	0.29679600
C	-1.13853000	3.12951700	0.28276800
H	-2.18471700	2.94508500	0.01839700
H	-0.90289500	4.17171900	0.04746800
H	-0.49914500	2.48570400	-0.32751700

**TS-d**			
Single-point energy (Eh)		-205.6008411	
Gibbs free energy (Eh)		-205.6101419	
**Atom**	**X**	**Y**	**Z**
N	-0.30060600	-0.06638000	0.00000000
O	-1.18025100	0.73771100	0.00000000
O	0.97466500	0.27041900	0.00000000
H	0.50619300	-0.94174900	0.00000000

10 References

[1] a) S. Saha, S. Maity, B. Samanta, R. Ghosh, K. Bhattacharyya, B. Mondal, "Photo-induced nitroxyl anion/HNO release from a nitrosyl complex of Mn(ii)–porphyrinate" *Chemical Communications* **61**, (2025): 2353–2356; b) S. Fujii, T. Yoshimura, "A new trend in iron–dithiocarbamate complexes: as an endogenous NO trapping agent" *Coordination Chemistry Reviews* **198**, (2000): 89–99.

[2] S. Stoll, A. Schweiger, "EasySpin, a comprehensive software package for spectral simulation and analysis in EPR" *Journal of Magnetic Resonance* **178**, (2006): 42–55.

[3] F. Neese, "Software Update: The ORCA Program System—Version 6.0" *WIREs Computational Molecular Science* **15**, (2025): e70019.

[4] J. W. Furness, A. D. Kaplan, J. Ning, J. P. Perdew, J. Sun, "Accurate and Numerically Efficient r2SCAN Meta-Generalized Gradient Approximation" *The Journal of Physical Chemistry Letters* **11**, (2020): 8208–8215.

[5] S. Grimme, A. Hansen, S. Ehlert, J.-M. Mewes, "r2SCAN-3c: A “Swiss army knife” composite electronic-structure method" *The Journal of Chemical Physics* **154**, (2021): 064103.

[6] E. Caldeweyher, S. Ehlert, A. Hansen, H. Neugebauer, S. Spicher, C. Bannwarth, S. Grimme, "A generally applicable atomic-charge dependent London dispersion correction" *The Journal of Chemical Physics* **150**, (2019): 154122.

[7] H. Kruse, S. Grimme, "A geometrical correction for the inter- and intra-molecular basis set superposition error in Hartree-Fock and density functional theory calculations for large systems" *The Journal of Chemical Physics* **136**, (2012): 154101.

[8] V. Barone, M. Cossi, "Quantum Calculation of Molecular Energies and Energy Gradients in Solution by a Conductor Solvent Model" *The Journal of Physical Chemistry A* **102**, (1998): 1995–2001.

[9] M. Ju, J. Kim, J. Shin, "EPR spectroscopy: A versatile tool for exploring transition metal complexes in organometallic and bioinorganic chemistry" *Bulletin of the Korean Chemical Society* **45**, (2024): 835–862.

[10] P. Gray, A. D. Yoffe, "The Reactivity And Structure Of Nitrogen Dioxide" *Chemcal Reviews* **55**, (1955): 1069–1154.

[11] R. Que, W. Ding, S. Sha, "A Simple, Facile Demonstration of Copper and Nitric Acid Reaction" *Journal of Chemical Education* **99**, (2022): 2762–2765.

[12] T. Yoshimura, Y. Kotake, "Spin Trapping of Nitric Oxide with the Iron-Dithiocarbamate Complex: Chemistry and Biology" *Antioxidants & Redox Signaling* **6**, (2004): 639–647.

[13] V. Hosseininasab, I. M. DiMucci, P. Ghosh, J. A. Bertke, S. Chandrasekharan, C. J. Titus, D. Nordlund, J. H. Freed, K. M. Lancaster, T. H. Warren, "Lewis acid-assisted reduction of nitrite to nitric and nitrous oxides via the elusive nitrite radical dianion" *Nature Chemistry* **14**, (2022): 1265–1269.

[14] K. A. Rosadiuk, D. S. Bohle, "Anhydrous Dinitrogen Trioxide Solutions for Brønsted Acid Free Nitrous Acid Chemistry" *European Journal of Inorganic Chemistry* **2017**, 2017: 5461–5465.

[15] S. G. Kukolich, "The structure of the nitric oxide dimer" *Journal of the American Chemical Society* **104**, (1982): 4715–4716.

[16] a) M. Mehta, J. M. Goicoechea, "Nitrenium Salts in Lewis Acid Catalysis" *Angewandte Chemie International Edition* **59**, (2019): 2715–2719; b) A. Baschieri, R. Amorati, L. Valgimigli, L. Sambri, "1-Methyl-1,4-cyclohexadiene as a Traceless Reducing Agent for the Synthesis of Catechols and Hydroquinones" *The Journal of Organic Chemistry* **84**, (2019): 13655–13664.
